# Supplementary material for: Human mobility networks reveal increased segregation in large cities
Source: Nature. 2023 Nov 29;624(7992):586–92. doi: 10.1038/s41586-023-06757-3 (PMC10733138; doi:10.1038/s41586-023-06757-3)
Supplement: Supplementary file 1 — Supplementary Figs. 1–66 and Supplementary Tables 1–24. [file 41586_2023_6757_MOESM1_ESM.pdf]

nature portfolio

https://doi.org/10.1038/s41586-023-06757-3

Supplementary information

Human mobility networks reveal increased segregation in large cities

In the format provided by the authors and unedited

Supplementary Information

Descriptive statistics. We include high-level descriptive statistics of exposure network, users, and MSAs in Supplementary Table S1 and Supplementary Figure S1. For detailed descriptive statistics of the exposure network see:

- Supplementary Table S8 Distribution of number of exposures
- Supplementary Table S9 Distribution of number of exposures (per active day)
- Supplementary Table S10 Distribution of tie strength
- Supplementary Table S17 Distribution of number of distinct exposures
- Supplementary Table S18 Distribution of number of distinct exposures (per active day)
- Supplementary Figure S28 Average number of exposures over time
- Supplementary Figure S27 Number of active individuals over time
- Supplementary Figure S30 Total exposures over time

For descriptive statistics of robustness checks over time, distance, and length of exposure thresholds, see Supplementary Tables S19 and S20, and Supplementary Figures S38-S61.

For descriptive statistics of POIs (count, average SES, average exposure segregation, and average number of exposures) see Supplementary Tables S2-S5.

Robustness Checks. For a high-level overview of all robustness checks, see Supplementary Table S6. For details of each robustness check, including nationwide correlations between exposure segregation, population size, and bridging index, see:

- Supplementary Figure S2 Weighting exposures by repetition
- Supplementary Figure S3 Varying definitions of socioeconomic status
- Supplementary Figure S4 Excluding exposures within roads, with residents of the same home, and in non-work/leisure contexts
- Supplementary Figure S5 Varying minimum distance between pings
- Supplementary Figure S6 Varying minimum time between pings
- Supplementary Figures S7-S8 Varying minimum tie strength
- Supplementary Figure S9 Controlling for background density
- Supplementary Figure S10 Filtering for exposure location
- Supplementary Figure S34 Racial segregation and economic segregation within race groups

- Supplementary Figure S62 Varying minimum stationary nights

Null models. For null models of alternative homophily mechanisms, which do not explain segregation in large cities, see Supplementary Figures S11 and S12.

Bridging Index. For an explanation of why bridging index predicts exposure segregation, see S13. Supplementary Figure S14 shows that the hub bridging finding is robust to definition of SES diversity, and Supplementary Figure S15 shows null results for other alternatives to the hub bridging finding. Supplementary Figures S16-S18 show illustrative examples of hubs. Supplementary Figure S65 explores Montgomery, AL, a city with a low bridging index.

Comparisons to conventional segregation measures. Supplementary Figures S19 and S20 compare exposure segregation to conventional residential segregation (neighborhood sorting index). Supplementary Figure S64 shows that using home Census tract as a proxy for SES, as is common in prior work, is inaccurate. Supplementary Figure S66 compares (racial) exposure segregation to experienced isolation from Athey et al. [1]

Associations with downstream outcomes. Supplementary Figure S24 shows that exposure segregation predicts political polarization. Finally, Supplementary Figures S32 and Supplementary Tables S23-S24 show that our exposure network predicts friendship formation, even when controlling for distance.

Robustness to noise. We show that our network size is sufficient to draw statistical comparisons between large and small cities, and that our findings are robust to noise via bootstrapped confidence intervals (Supplementary Figure S25) and by downsampling the network and reproducing our findings (Supplementary Figure S26).

Types of exposures by tie strength. We show how different venues vary by exposure reptition, length, and distance in Supplementary Tables S14, S15, and S16. Supplementary Figure S23 shows that exposure segregation predicts upward economic mobility, regardless of threshold on tie strength.

Race. Supplementary Figure S35 shows how POIs segregation varies by racial and economic segregation. Supplementary Figure S34 compares economic segregation to racial segregation, and splits economic segregation by race group.

Temporal heterogeneity in segregation. Supplementary Figure S21 shows that exposure segregation varies by time, which is further illustrated by examples in Supplementary Figures S63.

Miscellaneous. For additional anlayses, see:

- Supplementary Figure S22 POI differentiation in large cities is robust to POI category
- Supplementary Table S21 Exposure Segregation and related variables (i.e. # exposures, mean SES, neighborhood sorting index, Gini index, population size, and bridging index for all 382 MSAs

- Supplementary Figure S7 Population density finding robustness
- Supplementary Figure S31 Findings generalize to rural counties
- Supplementary Figure S66 shows how using our time-sensitive and high-resolution definition of exposure is necessary to reject the cosmopolitan mixing hypothesis—compared to prior work's (time-insensitive) definitions of exposure which yields null results

Table of Contents

List of Supplementary Figures

pg. #

1. Descriptive statistics of exposures……………………………………………….…….8
2. Robustness of primary study findings to weighting of repeated exposures……………..12
3. Robustness of primary study findings to definition of socioeconomic status…………...13
4. Robustness of primary study findings to exclusion of exposures within roads, exclusion exposures with residents of the same home, and exclusion of non-work/leisure exposures……………………………………………………………………………14
5. Robustness of primary study findings to minimum distance required between two GPS pings for individuals to be considered crossing paths…………………………...15
6. Robustness of primary study findings to minimum time elapsed between two pings to constitute an exposure………………………………………………………………16
7. Robustness of primary study findings to minimum tie strength required to constitute an exposure…………………………………………………………………….....17
8. (continued from Supplementary Figure S7) Robustness of primary study findings to minimum tie strength required to constitute an exposure. ………………………………18
9. Robustness of primary study findings to background density of exposures……………..19
10. Robustness of primary study findings to including only exposures in which both individuals, one individual, or neither individuals were located within their home tract at the time of exposure…………………………….……………………………………..20
11. Alternative homophily mechanisms do not explain segregation in large cities……….....21
12. Baseline Homophily null model results are robust to varying null model specifications……………………………………………………………………….....22
13. Understanding why the bridging index explains exposure segregation……………….....23
14. Robustness of the bridging index to definition of SES diversity……………………..….24
15. We consider alternative processes through which built environment may mitigate exposure segregation………………………………………………………..………25
16. Examples of exposure hubs in coastal cities………………………………..………26
17. Example of a major hubs in Fayetteville, NC…………………………….….……..27
18. Examples two hubs in Montgomery, AL…………………………………..……….28
19. Our model with simulated uniform within-tract crossings is equivalent to the conventional neighborhood sorting index. ………………………………….……..29
20. Our segregation measure versus a conventional residential segregation measure, neighborhood sorting index……………………………………………….……..29
21. Segregation decomposed by time.………………………………………….……30
22. Across many activities, POIs in large cities are more differentiated and consequently more segregated………………………………………………………………31-32
23. We measure the external validity of alternative measures in which the strictness of our exposure definition is varied…………………………………………..………35
24. Exposure segregation predicts political polarization outcomes……………..………...36

25. Precision of exposure segregation estimates……………………………….………….37
26. Robustness of primary findings to network size. ………………………..………38
27. Active individuals over time. ……………………………………………..………..40
28. Average number of exposures over time..…………………………………..………..41
29. Individuals with data coverage over time……………………………..………46
30. Exposures over time across all individuals residing in the 382 MSAs………..………47
31. Counties grouped by extent of county urbanization……………………………..……48
32. Exposure network connections strongly correlate to friendship formation even within fine-grained geographical areas………………………………………………..……..49
33. We measure the external validity of alternative measures in which the strictness of our exposure definition is varied…………………………………………………..……51
34. Racial exposure segregation, and economic exposure segregation broken down by race group……………………………………………………………………………..……52
35. Comparing economic and racial exposure segregation by POI………………..……...53
36. Average number of distinct exposures over time……………………………..……56
37. Distinct exposures over time across all individuals residing in the 382 MSAs………....57
38. Active individuals over time (exposure distance threshold: 25 meters)……………..…..59
39. Active individuals over time (exposure distance threshold: 10 meters)……………..…..60
40. Active individuals over time (exposure time threshold: 2 minutes)…………..………61
41. Active individuals over time (exposure time threshold: 60 seconds)………………..……..62
42. Active individuals over time (exposure length threshold: 2 consecutive exposures over five minute intervals) ……………………………………………………………..……..63
43. Active individuals over time (exposure length threshold: 3 consecutive exposures over five minute intervals) ……………………………………………………………..……..64
44. Active individuals over time (distance threshold: 25 meters, time threshold: 2 minutes, length threshold: 2 consecutive exposures of five minute intervals)…………………..…...65
45. Active individuals over time (distance threshold: 10 meters, time threshold: 60 seconds, length threshold: 3 consecutive exposures of five minute intervals)…………………..…...66
46. Average number of distinct exposures over time (distance threshold: 25 meters)…...….67
47. Average number of distinct exposures over time (distance threshold: 10 meters)………68
48. Average number of distinct exposures over time (time threshold: 2 minutes)…………..69
49. Average number of distinct exposures over time (time threshold: 60 seconds)……..…..70
50. Average number of distinct exposures over time (exposure length threshold: 2 consecutive exposures over five minute intervals) ………………………..……….71
51. Average number of distinct exposures over time (exposure length threshold: 3 consecutive exposures over five minute intervals) …………………………..……72
52. Average number of distinct exposures over time (distance threshold: 25 meters, time threshold: 2 minutes, length threshold: 2 consecutive exposures of five minute intervals)……………………..……………………………….…………73
53. Average number of distinct exposures over time (distance threshold: 10 meters, time threshold: 60 seconds, length threshold: 3 consecutive exposures of five minute intervals) ………………………………………………………………………..……….74
54. Number of distinct exposures (distance threshold: 25 meters) ……………..………...75
55. Number of distinct exposures (distance threshold: 10 meters) ……………..………...76
56. Number of distinct exposures (time threshold: 2 minutes) …………………..……….77
57. Number of distinct exposures (time threshold: 60 seconds) …………………..……...78

58. Number of distinct exposures (exposure length threshold: 2 consecutive exposures over five minute intervals) ……………………………………………………………..………..79
59. Number of distinct exposures (exposure length threshold: 3 consecutive exposures over five minute intervals) ………………………………………………………………..……..80
60. Number of distinct exposures (distance threshold: 25 meters, time threshold: 2 minutes, length threshold: 2 consecutive exposures of five minute intervals)………………..…...86
61. Number of distinct exposures (distance threshold: 10 meters, time threshold: 60 seconds, length threshold: 3 consecutive exposures of five minute intervals) …………………....87
62. Varying minimum stationary nights……………………………………………………..…89
63. Temporal heterogeneity in exposure participants at the same location: illustrative examples……………………………………………………………………………....90
64. Error from using census block group (CBG) income as a proxy for SES………..……...91
65. Montgomery, AL analysis analogous to Figure 3c-d…………………………...……..93
66. Our time-sensitive and high-resolution measure of exposure enables us to reject the cosmopolitan mixing hypothesis………………………………………………………94

List of Supplementary Tables

1. Combined descriptive statistics for all individuals residing in 382 Metropolitan Statistical Areas (MSAs) ……………………………………………………………………….....8
2. POI descriptive statistics (# of POIs in each MSA) for each of the fine-grained POI categories in Figure 2c……………………………………………………………………9
3. POI descriptive statistics (average POI socioeconomic status in an MSA) for each of the fine-grained POI categories in Figure 2c……………………………………………….....9
4. POI descriptive statistics (exposure segregation within-category) for each of the fine-grained POI categories in Figure 2c…………………………………………………...10
5. POI descriptive statistics (number of exposures occurring inside POI category) for each of the fine-grained POI categories in Figure 2c……………………………………......10
6. Robustness checks overview……………………………………………………….11
7. Population density is significantly associated with exposure segregation, after controlling key covariates………………………………………………………………………......33
8. Distribution of number of exposures for all individuals residing in 382 Metropolitan Statistical Areas (MSAs) ……………………………………………………….……..34
9. Distribution of average number of exposures (per active day) for all individuals residing in 382 Metropolitan Statistical Areas (MSAs) ………………………………………..…39
10. Distribution of tie strength (# of exposures) for all pairs of individuals residing in 382 Metropolitan Statistical Areas (MSAs) …………………………………………..……...42
11. Distribution of total pings for all included individuals residing in 382 Metropolitan Statistical Areas (MSAs) …………………………………………………………..…….43
12. Distribution of unique days of ping data coverage for all included individuals residing in 382 Metropolitan Statistical Areas (MSAs) ……………………………………………..…44
13. Distribution of interevent times (seconds elapsed between pings) for all included individuals residing in 382 Metropolitan Statistical Areas (MSAs) …………….……...45
14. Exposure repetition by setting………..………………………………………….………48
15. Exposure distance by setting……………………………………………..………50
16. Exposure length by setting…………………………………………………..……...50

17. Distribution of number of distinct exposures for all individuals residing in 382 Metropolitan Statistical Areas (MSAs) ……………………………………………..……...54
18. Distribution of average number of distinct exposures (per active day) for all individuals residing in 382 Metropolitan Statistical Areas (MSAs) ………………………..……..55
19. Distribution of number of distinct exposures per person (by time, distance, and length threshold) ……………………………………………………………………….……58
20. Distribution of average number of distinct exposures (per active day) per person across all days of activity (by time, distance, and length threshold) ……………………..……..58
21. Exposure Segregation and related variables (i.e. # exposures, mean SES, neighborhood sorting index, Gini index, population size, and bridging index) by MSA…………..…...81
22. POI density is significantly associated with exposure segregation, after controlling for key covariates………………………………………………………………………..…...88
23. Our exposure network strongly predicts friendship formation (between counties) ………………………………………………………………………………..……..91
24. Our exposure network strongly predicts friendship formation (between zip codes) ……………………………………………………………………………………....92

	Accurate pings	Unique days	Distinct user pairs	Exposures	Accurate pings	Distinct user pairs	Exposures
count	8,609,406				382		
mean	3,273	35	184	363	73,757,695	2,577,322	4,845,144
std	16,507	20	374	1,073	163,848,305	8,872,464	16,838,938
min	11	2	1	1	2,196,084	27,326	53,350
10%	570	13	8	17	8,398,875	140,251	313,803
50%	1,471	30	76	141	22,054,930	504,525	1,031,691
90%	5,857	63	436	785	175,295,175	4,573,152	8,954,800
max	4,755,081	95	42,323	193,193	1,605,070,032	94,140,015	215,183,409

Supplementary Table S1: Combined descriptive statistics for all individuals residing in 382 Metropolitan Statistical Areas (MSAs). 8,609,406 individuals reside in a Metropolitan Statistical Area (90% of the overall 9,567,559 individuals in our study). The remaining 958,153 users live outside of MSAs, influencing the exposure segregation of an MSA by coming into contact with MSA residents. Descriptive statistics are grouped by individual (left) and MSA (right). At least one of two users in each exposure pair must live in an MSA to be included in this table.

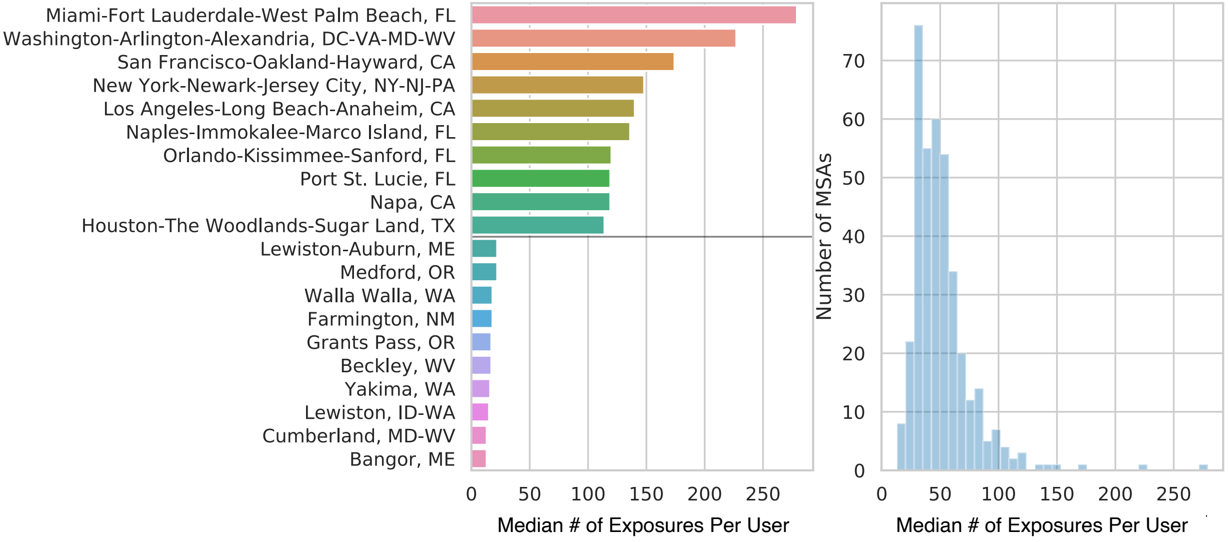

Supplementary Figure S1: Descriptive statistics of exposures.
(a) Ten Metropolitan Statistical Areas (MSAs) with the highest and lowest median number of exposures per user.
(b) Overall distribution of median number of exposures per user over MSAs.

POI Type	# POIs (25%)	# POIs (50%)	# POIs (75%)	# POIs (max)	# POIs (mean)	# POIs (min)	# POIs (std)
Full-Service Restaurants	75.5	160.0	424.0	24,689.0	609.8	12.0	1,820.05
Snack Bars	18.0	40.0	110.0	6,266.0	169.76	1.0	511.17
Limited-Service Restaurants	33.0	60.0	145.5	4,847.0	192.14	5.0	434.78
Stadiums	1.0	2.0	4.0	43.0	3.67	1.0	4.32
Performing Arts Centers	1.0	2.0	4.0	28.0	3.25	1.0	3.41
Fitness/Recreation Centers	10.0	25.0	72.0	4,877.0	126.6	1.0	414.26
Historical Sites	1.0	2.0	7.0	206.0	9.16	1.0	21.65
Theme Parks	1.0	3.0	6.0	158.0	7.64	1.0	16.77
Bars/Drinking Places	2.0	5.0	13.0	447.0	19.52	1.0	45.91
Parks	3.0	6.0	17.0	793.0	28.69	1.0	80.44
Religious Organizations	7.0	16.0	41.25	2,644.0	63.28	1.0	196.97
Bowling Centers	2.0	4.0	8.0	204.0	9.77	1.0	20.52
Museums	1.0	3.0	6.0	137.0	6.78	1.0	13.99
Casinos	1.0	3.0	7.0	188.0	8.05	1.0	17.22
Independent Artists	1.0	2.0	5.0	130.0	7.55	1.0	17.42
Other Amusement/Recreation	1.0	2.0	7.0	525.0	10.17	1.0	36.13
Golf Courses and Country Clubs	2.0	3.0	7.0	101.0	8.07	1.0	13.77

Supplementary Table S2: POI descriptive statistics (# of POIs in each MSA) for each of the fine-grained POI categories in Figure 2c.

POI Type	POI SES (25%)	POI SES (50%)	POI SES (75%)	POI SES (max)	POI SES (mean)	POI SES (min)	POI SES (std)
Full-Service Restaurants	1,210.96	1,395.0	1,674.27	3,628.06	1,493.04	763.0	430.99
Snack Bars	1,229.73	1,412.35	1,684.69	3,621.34	1,513.61	788.12	433.86
Limited-Service Restaurants	1,174.84	1,351.64	1,587.4	3,501.19	1,440.15	771.34	410.95
Stadiums	1,310.0	1,500.0	1,775.0	3,585.25	1,593.21	795.0	424.77
Performing Arts Centers	1,395.0	1,583.1	1,832.4	3,632.78	1,659.56	875.0	431.06
Fitness/Recreation Centers	1,230.03	1,431.79	1,703.94	3,749.05	1,528.73	700.0	453.4
Historical Sites	1,325.0	1,527.94	1,793.75	3,618.58	1,627.62	757.5	452.96
Theme Parks	1,300.0	1,498.75	1,750.0	3,900.0	1,612.58	700.0	501.79
Bars/Drinking Places	1,220.02	1,420.25	1,676.4	3,656.17	1,505.86	750.0	440.08
Parks	1,279.82	1,470.15	1,748.12	3,748.11	1,562.62	725.0	454.13
Religious Organizations	1,269.27	1,459.86	1,677.08	3,670.38	1,529.02	754.0	428.42
Bowling Centers	1,180.08	1,368.75	1,621.15	3,504.36	1,457.96	725.0	434.56
Museums	1,275.0	1,490.83	1,775.36	3,606.66	1,585.92	800.0	474.37
Casinos	1,200.0	1,400.0	1,655.54	3,606.17	1,503.88	725.0	469.68
Independent Artists	1,374.38	1,611.5	1,904.6	3,691.68	1,725.42	850.0	528.33
Other Amusement/Recreation	1,266.0	1,450.0	1,700.74	4,053.39	1,549.13	758.0	462.03
Golf Courses and Country Clubs	1,399.06	1,648.4	1,964.19	4,248.5	1,765.92	900.0	542.13

Supplementary Table S3: POI descriptive statistics (average POI socioeconomic status in an MSA) for each of the fine-grained POI categories in Figure 2c. POI socioeconomic status is operationalized as the median visitor SES of the POI.

POI Type	Exposure Segregation: (25%)	(50%)	(75%)	(max)	(mean)	(min)	(std)
Full-Service Restaurants	0.22	0.27	0.32	0.48	0.27	0.08	0.07
Snack Bars	0.2	0.25	0.31	0.5	0.25	0.01	0.08
Limited-Service Restaurants	0.24	0.29	0.34	0.47	0.29	0.04	0.08
Stadiums	0.14	0.17	0.22	0.36	0.18	0.02	0.06
Performing Arts Centers	0.14	0.16	0.19	0.27	0.17	0.05	0.05
Fitness/Recreation Centers	0.2	0.26	0.31	0.47	0.25	0.03	0.08
Historical Sites	0.15	0.2	0.27	0.43	0.21	0.0	0.09
Theme Parks	0.16	0.2	0.25	0.42	0.2	0.02	0.08
Bars/Drinking Places	0.18	0.23	0.3	0.42	0.23	0.06	0.08
Parks	0.19	0.26	0.33	0.47	0.26	0.05	0.09
Religious Organizations	0.24	0.32	0.38	0.55	0.31	0.05	0.1
Bowling Centers	0.16	0.21	0.26	0.44	0.22	0.03	0.08
Museums	0.18	0.22	0.28	0.45	0.24	0.06	0.08
Casinos	0.2	0.26	0.32	0.47	0.26	0.02	0.09
Independent Artists	0.13	0.2	0.27	0.39	0.21	0.02	0.09
Other Amusement/Recreation	0.18	0.25	0.31	0.71	0.25	0.02	0.12
Golf Courses and Country Clubs	0.33	0.41	0.5	0.62	0.4	0.2	0.11

Supplementary Table S4: POI descriptive statistics (exposure segregation within-category) for each of the fine-grained POI categories in Figure 2c. Exposure segregation is calculated for each POI category by filtering for only exposures which occurred inside of the POI category, before estimating exposure segregation (Methods 'Estimating exposure segregation').

POI Type	# Exposures (25%)	# Exposures (50%)	# Exposures (75%)	# Exposures (max)	# Exposures (mean)	# Exposures (min)	# Exposures (std)
Full-Service Restaurants	23,060.75	54,219.5	156,645.0	19,540,673.0	398,304.04	6,112.0	1,634,147.68
Snack Bars	15,582.0	38,954.0	120,225.0	14,128,466.0	291,523.01	5,233.0	1,205,873.07
Limited-Service Restaurants	16,485.5	38,444.0	106,515.0	10,453,353.0	227,378.5	4,122.0	878,243.0
Stadiums	53,077.5	96,487.5	336,479.75	8,942,618.0	348,920.85	17,024.0	988,719.72
Performing Arts Centers	66,712.0	120,256.0	384,770.0	6,972,326.0	403,378.22	27,589.0	932,589.88
Fitness/Recreation Centers	11,165.25	21,541.5	62,380.5	5,630,299.0	158,025.53	3,740.0	573,788.93
Historical Sites	18,351.5	47,793.0	98,385.5	6,362,665.0	187,978.09	5,147.0	684,470.65
Theme Parks	34,989.0	61,553.0	135,744.0	1,883,136.0	157,622.73	14,460.0	290,329.93
Bars/Drinking Places	11,592.5	21,401.0	63,929.0	1,266,235.0	84,752.14	4,553.0	181,978.58
Parks	10,050.75	22,301.0	61,492.75	1,520,092.0	88,789.84	5,383.0	193,888.94
Religious Organizations	6,014.0	13,002.0	35,157.25	2,206,316.0	60,683.34	2,739.0	212,948.46
Bowling Centers	16,423.5	26,517.0	65,563.5	1,030,970.0	92,515.06	5,874.0	174,876.21
Museums	14,807.5	27,802.0	66,729.75	681,994.0	87,797.02	4,310.0	146,495.57
Casinos	13,844.0	23,109.0	60,222.0	826,676.0	68,012.52	7,474.0	124,063.14
Independent Artists	8,795.0	23,789.5	56,736.75	1,106,402.0	87,662.95	3,951.0	198,394.53
Other Amusement/Recreation	6,923.0	14,349.0	42,793.0	365,436.0	38,640.52	2,929.0	56,964.16
Golf Courses and Country Clubs	4,765.75	7,836.5	15,555.0	58,348.0	13,047.42	2,636.0	13,348.76

Supplementary Table S5: POI descriptive statistics (number of exposures occurring inside POI category) for each of the fine-grained POI categories in Figure 2c.

Feature	Pearson Corr. w/ Primary	Spearman Corr. w/ Primary	Median	Mean	% Pairs	% People
Primary Measure	—	—	0.35	0.35	100.00	100.00
Primary Measure (+ Up-weight Multiple Exposures)	0.89	0.91	0.46	0.45	100.00	100.00
SES Definition: Rent Zestimate Percentile	0.88	0.89	0.42	0.42	100.00	100.00
SES Definition: Within-MSA Rent Zestimate Percentile	0.81	0.83	0.54	0.53	100.00	100.00
SES Definition: Census Median Household Income	0.75	0.77	0.47	0.46	100.00	100.00
SES Definition: Educational Attainment (% College or Higher)	0.70	0.71	0.52	0.52	100.00	100.00
Exclude Pri/Sec Roads	0.99	0.99	0.37	0.37	74.30	99.87
Exclude Roads	0.98	0.98	0.37	0.37	38.66	98.56
Stationary Individuals (2 pings $<$ 10 meters in 1-10 min)	0.86	0.87	0.44	0.43	5.39	79.46
Exclude Same-home exposures	0.98	0.98	0.34	0.34	99.71	99.78
Work/Leisure (Neither in Home Tract)	0.93	0.93	0.31	0.31	86.26	95.55
Visitation (One Person in Home Tract)	0.91	0.91	0.26	0.26	12.63	93.99
Both in Home Tract	0.62	0.63	0.73	0.72	2.32	82.35
Leisure (inside POI)	0.85	0.84	0.28	0.29	16.15	76.32
Minimum Distance Between Pings: $<$ 25 meters	0.97	0.97	0.34	0.34	49.53	97.71
Minimum Distance Between Pings: $<$ 10 meters	0.95	0.94	0.33	0.33	20.93	92.28
Minimum Time Between Pings: $<$ 2 minutes	0.97	0.97	0.34	0.34	53.59	98.92
Minimum Time Between Pings: $<$ 60 seconds	0.97	0.97	0.35	0.35	33.67	97.83
Minimum Tie Strength: 2 consecutive exposures	0.94	0.95	0.35	0.35	18.25	94.80
Minimum Tie Strength: 3 consecutive exposures	0.83	0.83	0.37	0.37	3.06	65.46
Minimum Tie Strength: 2 unique days of exposure	0.88	0.90	0.47	0.46	7.46	83.56
Minimum Tie Strength: 3 unique days of exposure	0.73	0.77	0.56	0.54	2.32	62.81
Dist. $<$ 25 meters, Time $<$ 2 min., $>=$ 2 consec. exposures	0.92	0.93	0.36	0.35	8.80	86.64
Dist. $<$ 25 meters, Time $<$ 2 min., $>=$ 2 unique days	0.87	0.89	0.46	0.44	3.84	70.57
Dist. $<$ 10 meters, Time $<$ 60 sec., $>=$3 consec. exposures	0.78	0.79	0.39	0.38	0.94	38.16
Dist. $<$10 meters, Time $<$ 60 sec., $>=$ 3 unique days	0.68	0.68	0.52	0.51	0.58	28.92
Downweight Simultaneous Exposures	0.97	0.97	0.34	0.34	99.99	99.99
Exclude Simultaneous Exposures	0.94	0.95	0.46	0.46	22.87	99.61
Tie Strength: 1 Exposure	0.97	0.98	0.31	0.32	74.24	98.66
Tie Strength: 2 Exposures	0.95	0.96	0.33	0.33	16.14	93.20
Tie Strength: 3 Exposures	0.89	0.90	0.36	0.36	4.09	74.67
Tie Strength: 4 Exposures	0.85	0.86	0.37	0.36	1.82	58.51
Tie Strength: 5+ Exposure	0.83	0.85	0.45	0.45	3.42	70.62
Minimum Stationary Nights: 6 nights	0.99	0.99	0.35	0.35	77.45	83.91
Minimum Stationary Nights: 9 nights	0.99	0.99	0.35	0.36	68.64	73.42
Minimum Stationary Nights: 12 nights	0.98	0.98	0.35	0.36	59.16	63.66
Racial Segregation (% Non-White)	0.59	0.60	0.46	0.46	100.0	100.0
Economic Segregation (White Overall)	0.94	0.95	0.34	0.34	55.97	63.83
Economic Segregation (White Within-group)	0.93	0.94	0.34	0.34	39.84	63.69
Economic Segregation (Non-White Overall)	0.55	0.52	0.28	0.28	43.76	35.50
Economic Segregation (Non-White Within-group)	0.47	0.44	0.28	0.30	27.50	35.31

Supplementary Table S6: Robustness checks overview. We find that our definition of exposure segregation is robust to varying many parameters: weighting of repeated exposures between the same users, definition of socioeconomic status, inclusion/exclusion of roads and same-home exposures, filtering location of exposure, minimum distance, minimum time, and minimum tie strength (as well as the intersection of distance, time, and tie strength). The above variants all are strongly correlated to our primary measure (all have Spearman Corr. $>=$ 0.75). We also find that our primary findings that (1) large, dense cities are more segregated and (2) hub locations accessible to diverse individuals may mitigate segregation are robust across all definitions of Exposure Segregation (Supplementary Figures S2-S10, S34, S62). Note that we exclude same-home exposures in robustness checks that vary minimum time, distance, or require repeated exposures, to ensure that results are not influenced by exposures with members of the same household (these exposures ordinarily have minimum influence on Exposure Segregation, as shown by the robustness check which excludes same-home exposures and results in virtually identical metric (Spearman Corr. 0.98); however, the influence of same-home exposures is higher after more conservative filters are applied to the definition of exposures, such as requiring a minimum tie strength of 3 consecutive exposure).

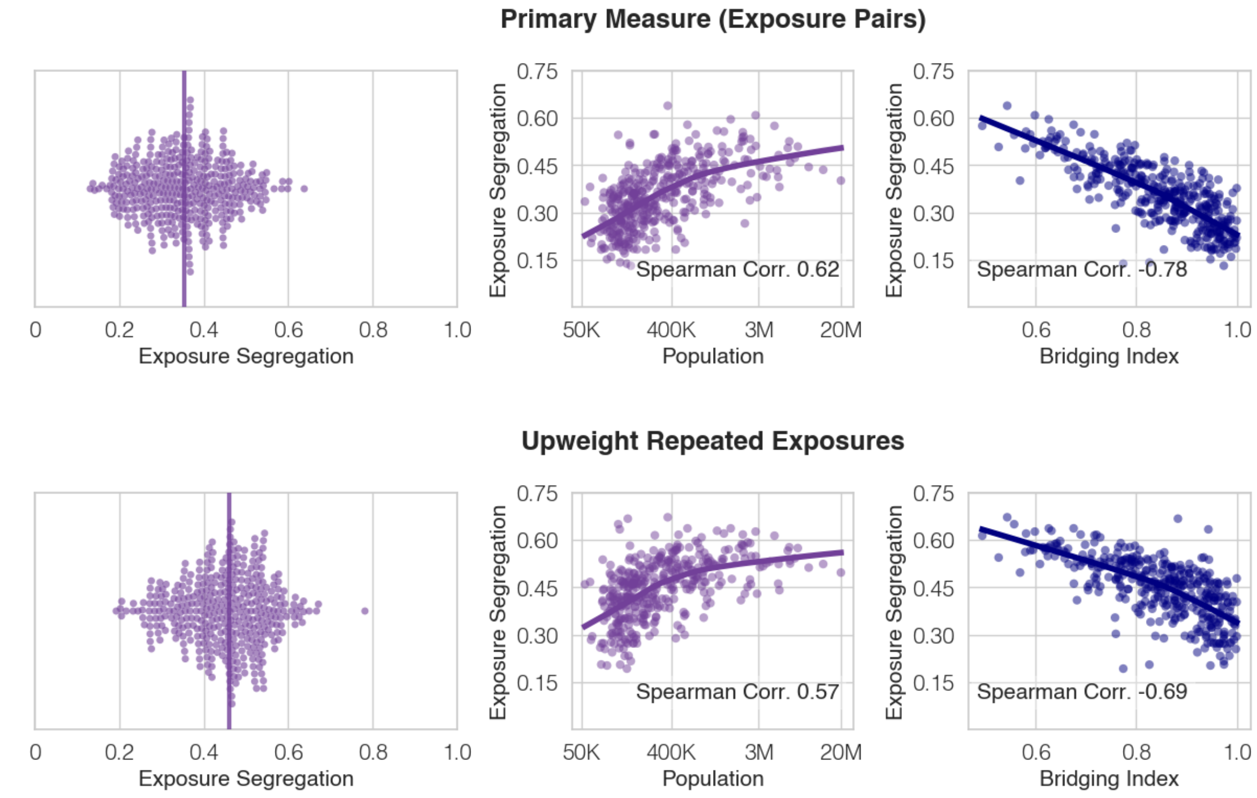

Supplementary Figure S2: Robustness of primary study findings to weighting of repeated exposures. We find that our primary study findings that, (1) large, dense cities are more segregated and (2) hub locations accessible to diverse individuals may mitigate segregation, are robust to the choice of whether to upweight repeated exposures in our exposure network. We compare the results of:

Primary Measure: Exposures are defined as pairs of users who have ever crossed paths within the study observation window (three months of 2017). We de-duplicate repeated exposures, as frequency of pings varies across smartphone users, to reduce bias from users with a higher frequency of pings. For instance, if an individual A with an individual B (ES $1000) two times and individual C (ES $2000) one, we compute the mean SES of individual A's network as $1500.

Upweight Repeated Exposures: Repeated exposures are unweighted when calculating the mean SES of an individual's exposure network. For instance, if an individual A with an individual B (ES $1000) two times and individual C (ES $2000) once, we compute the mean SES of individual A's network as $1333.

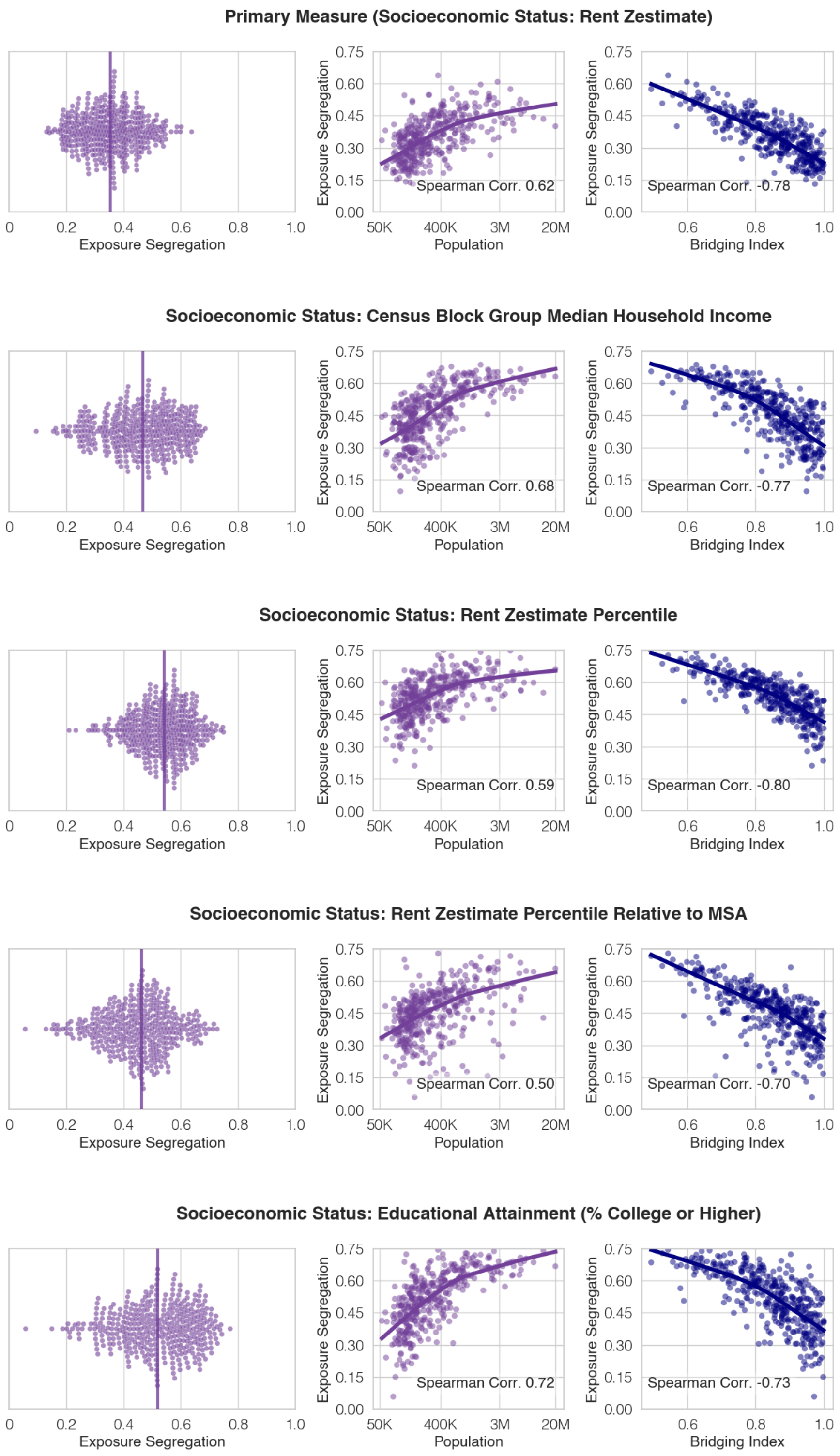

Supplementary Figure S3: Robustness of primary study findings to definition of socioeconomic status. We find that our primary study findings that, (1) large, dense cities are more segregated and (2) hub locations accessible to diverse individuals may mitigate segregation, are robust to the definition of socioeconomic status. We compare the results of:

Primary Measure: Our primary measure leverages estimated monthly rent value (Zillow Rent Zestimate).

Census Block Group (CBG) Median Household Income: We define the SES of an individual is the median household income in the CBG in which they reside.

Rent Zestimate Percentile: We normalize Rent Zestimate values across all individuals.

Primary measure Relative to MSA: We normalize Rent Zestimate values across all individuals within an MSA, independent of other MSAs, to account for differences in cost of living across cities.

Educational Attainment: We define the SES of an individual as their educational attainment (% with college education or higher).

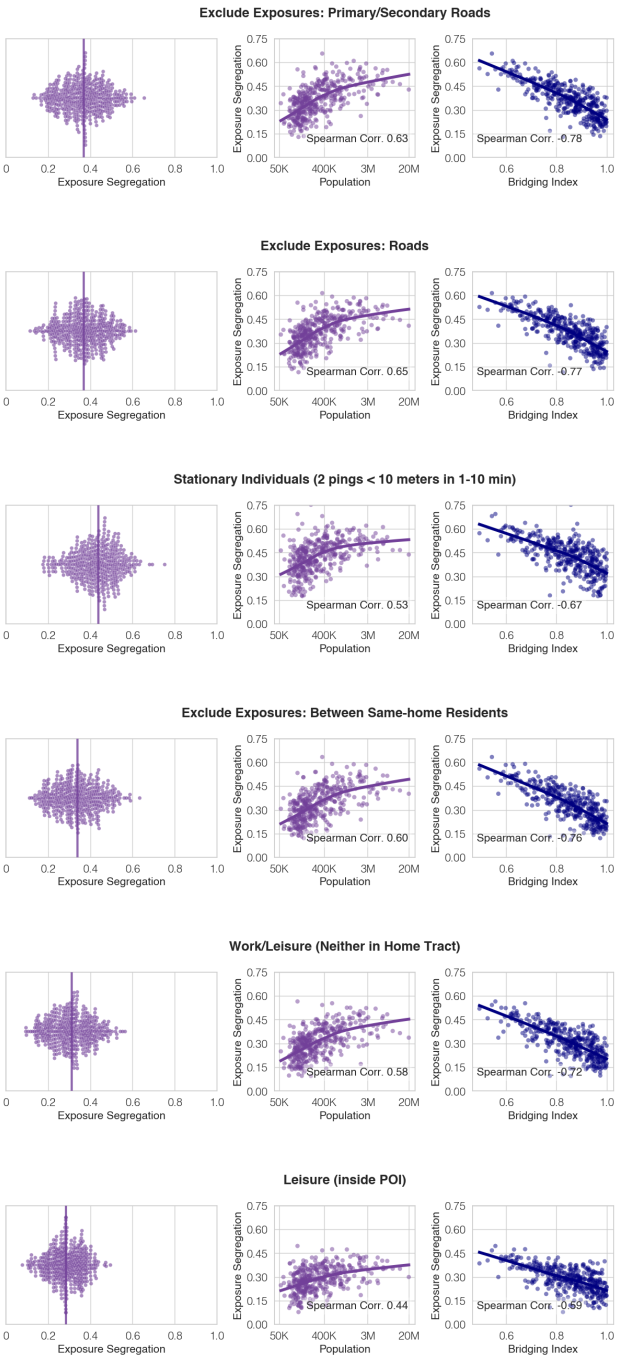

Supplementary Figure S4: Robustness of primary study findings to exclusion of exposures within roads, exclusion exposures with residents of the same home, and exclusion of non-work/leisure exposures. We find that our primary study findings that, (1) large, dense cities are more segregated and (2) hub locations accessible to diverse individuals may mitigate segregation, are robust to filtering for a subset of exposures. We compare the results of:
Primary Measure: Our primary measure includes all exposures, aiming to give a complete account of an individual's exposure network including path crossings on roads as well as those they share a home with.
Excluding (primary/secondary) roads: We filter to exclude exposures occurring on all roads, or only primary/secondary roads.
Only stationary pings: We filter to include only exposures occurring when individuals are stationary (i.e. have two pings within 1-10 minutes and $<$ 10 meters apart).
Same home residents: We filter to include only exposures occurring between two people residing in different homes.
Work/Leisure: We filter to include only exposures likely to take place in the context of work or leisure, by excluding exposures which occurred when either individuals were located within their home tracts.
Leisure: We filter for leisure exposures by including only exposures ocurring inside of the POIs categorized as related to leisure (Figure 2c).

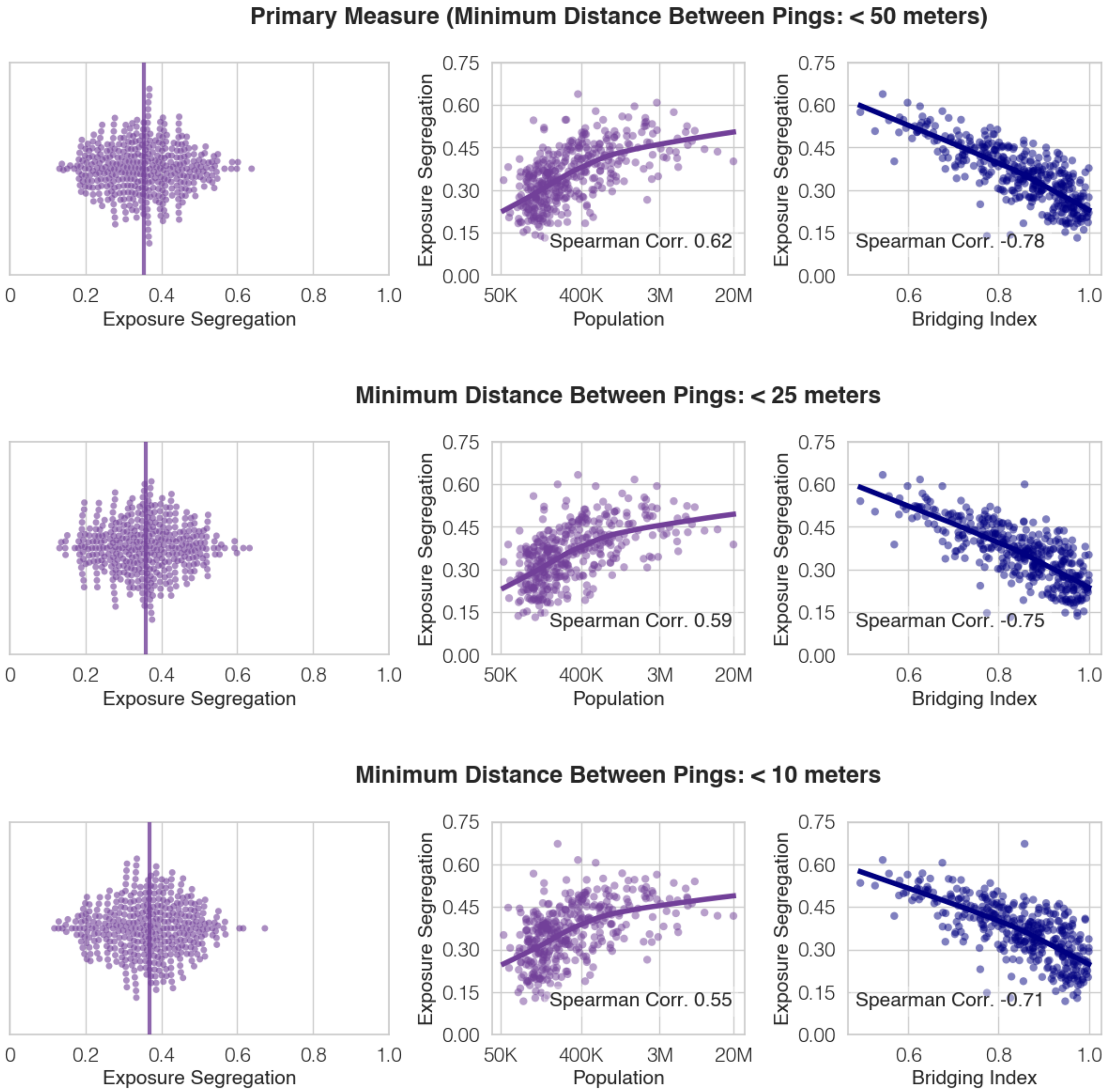

Supplementary Figure S5: Robustness of primary study findings to minimum distance required between two GPS pings for individuals to be considered crossing paths. We find that our primary study findings that, (1) large, dense cities are more segregated and (2) hub locations accessible to diverse individuals may mitigate segregation, are robust to the time threshold used in our definition of exposure:

Primary Measure: Our primary measure uses a threshold of 50 meters, based on prior literature which shows that even distant exposure to diverse individuals is predictive of long-term behaviors[2].

Alternative measures: We alternatively consider more conservative thresholds of 25 meters and 10 meters, with 10 meters being the lowest threshold due to limitations of GPS ping accuracy[3,4].

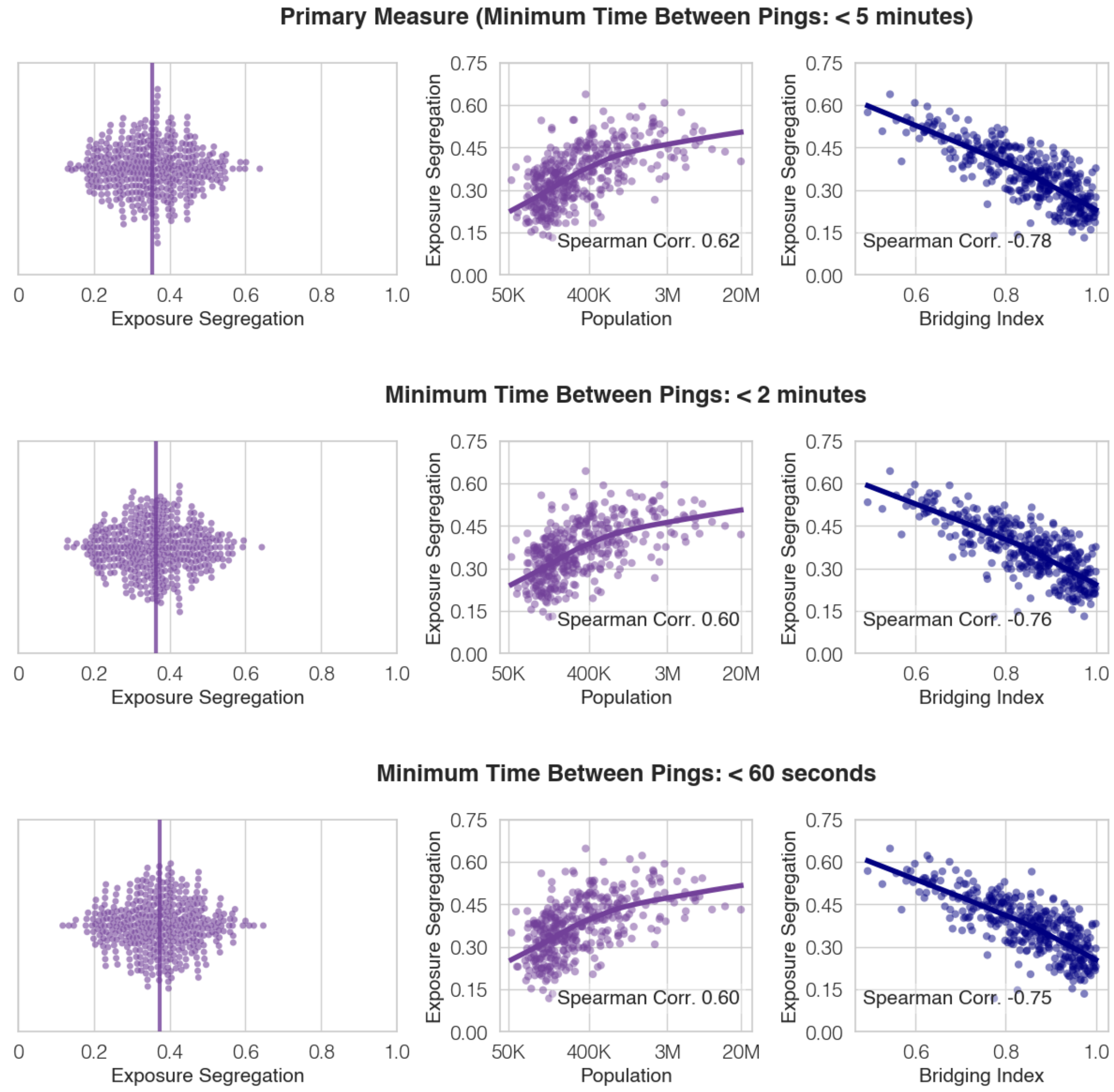

Supplementary Figure S6: Robustness of primary study findings to minimum time elapsed between two pings to constitute an exposure. We find that our primary study findings that, (1) large, dense cities are more segregated and (2) hub locations accessible to diverse individuals may mitigate segregation, are robust to the time threshold used in our definition of exposure:

Primary Measure: Our primary measure uses a threshold of 5 minutes, to be inclusive of users with sparse pings (e.g., for a subset of users, we only have 1 ping per day, while for others we have 100+ pings per day) while maintaining a reasonable confidence that an exposure may have occurred.

Alternative measures: We alternatively consider more conservative thresholds of 2 minutes and 1 minute.

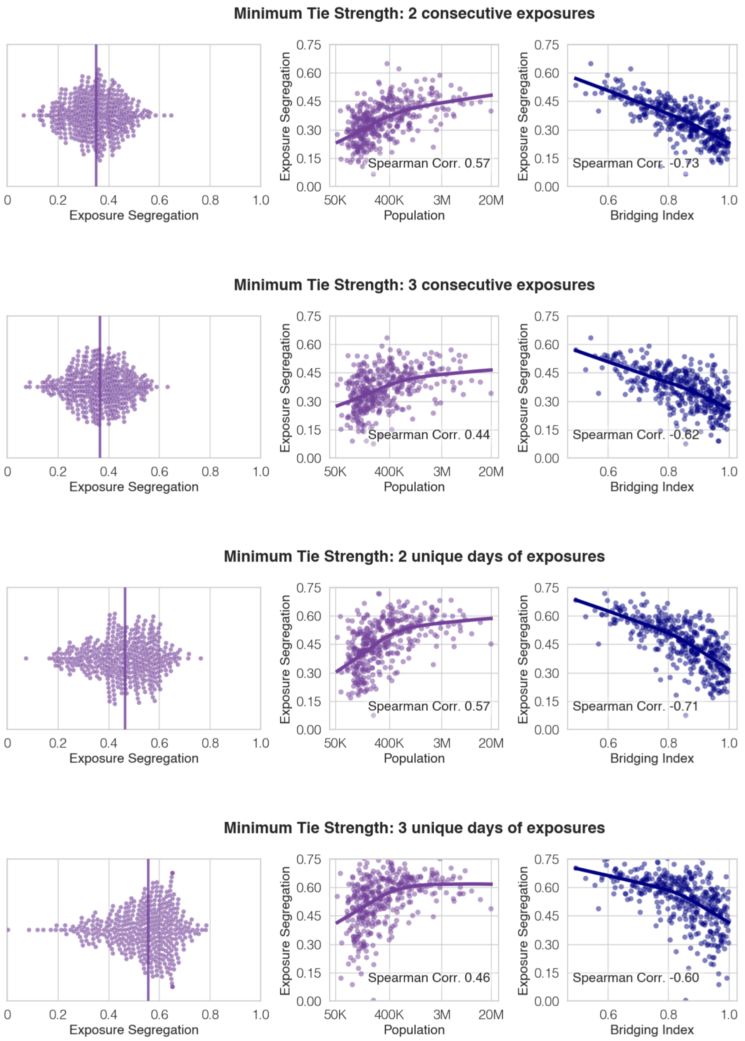

Supplementary Figure S7: Robustness of primary study findings to minimum tie strength required to constitute an exposure. We find that our primary study findings that, (1) large, dense cities are more segregated and (2) hub locations accessible to diverse individuals may mitigate segregation, are robust regardless of the minimum tie strength threshold between two individuals to be constitute an exposure:

Primary Measure: Our primary measure only requires a single pair of pings between users to constitute an exposure, to be inclusive of users with sparse pings (e.g., for a subset of users, we only have 1 ping per day, while for others we have 100+ pings per day).

Alternative measures: We alternatively consider more conservative thresholds of 2 or 3 consecutive exposures, as well as 2 or 3 exposures across unique days. Requiring consecutive exposures increases the likelihood that individuals actually came into contact together; exposures across unique days increases the likelihood that exposures are not merely path crossings, but social exposures between individuals who are familiar with each other. **(continued on next page)**

Distance: < 25 meters, Time: < 2 minutes, Minimum Tie Strength: 2 consecutive exposures

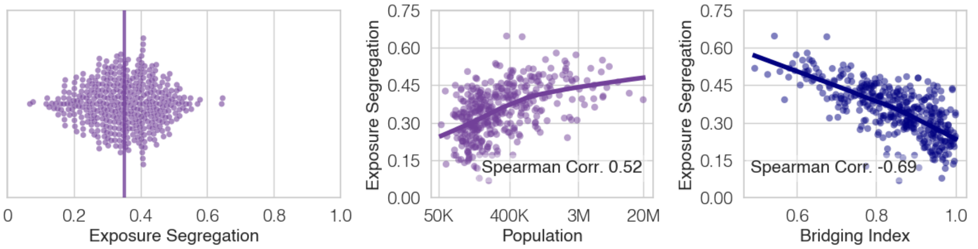

Distance: < 25 meters, Time: < 2 minutes, Minimum Tie Strength: 2 unique days of exposures

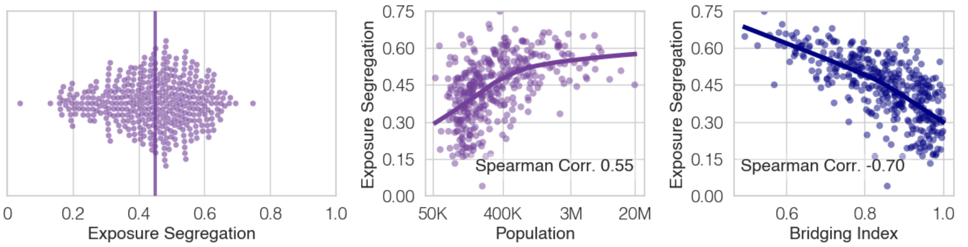

Distance: < 10 meters, Time: < 60 seconds, Minimum Tie Strength: 3 consecutive exposures

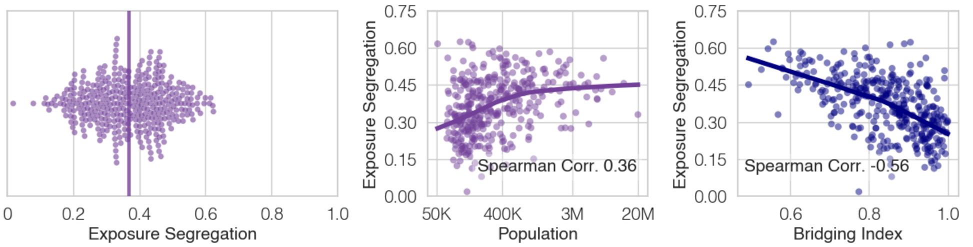

Distance: < 10 meters, Time: < 60 seconds, Minimum Tie Strength: 3 unique days of exposures

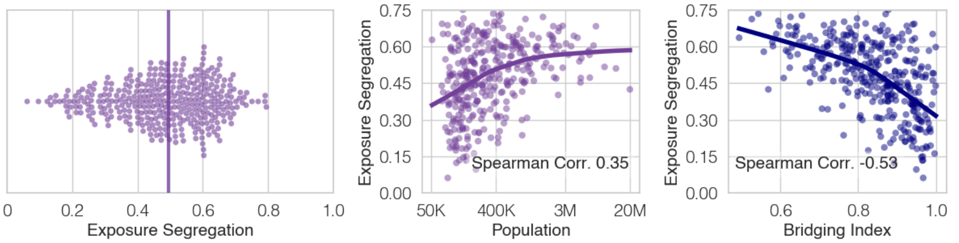

Supplementary Figure S8: (continued from previous page). We find that our primary study findings that, (1) large, dense cities are more segregated and (2) hub locations accessible to diverse individuals may mitigate segregation, are *robust to the combination of the minimum time, minimum distance, and minimum tie strength threshold parameters*. To account for exposures between threshold parameters, we also consider combinations of parameter variants. For instance, the most conservative robustness check defines an exposure as two individuals being $<$ 10 meters apart within a $<$ 60 second window, and for this to have occurred either for either 3 consecutive minutes (second figure from the bottom) or across 3 unique days (bottom figure).

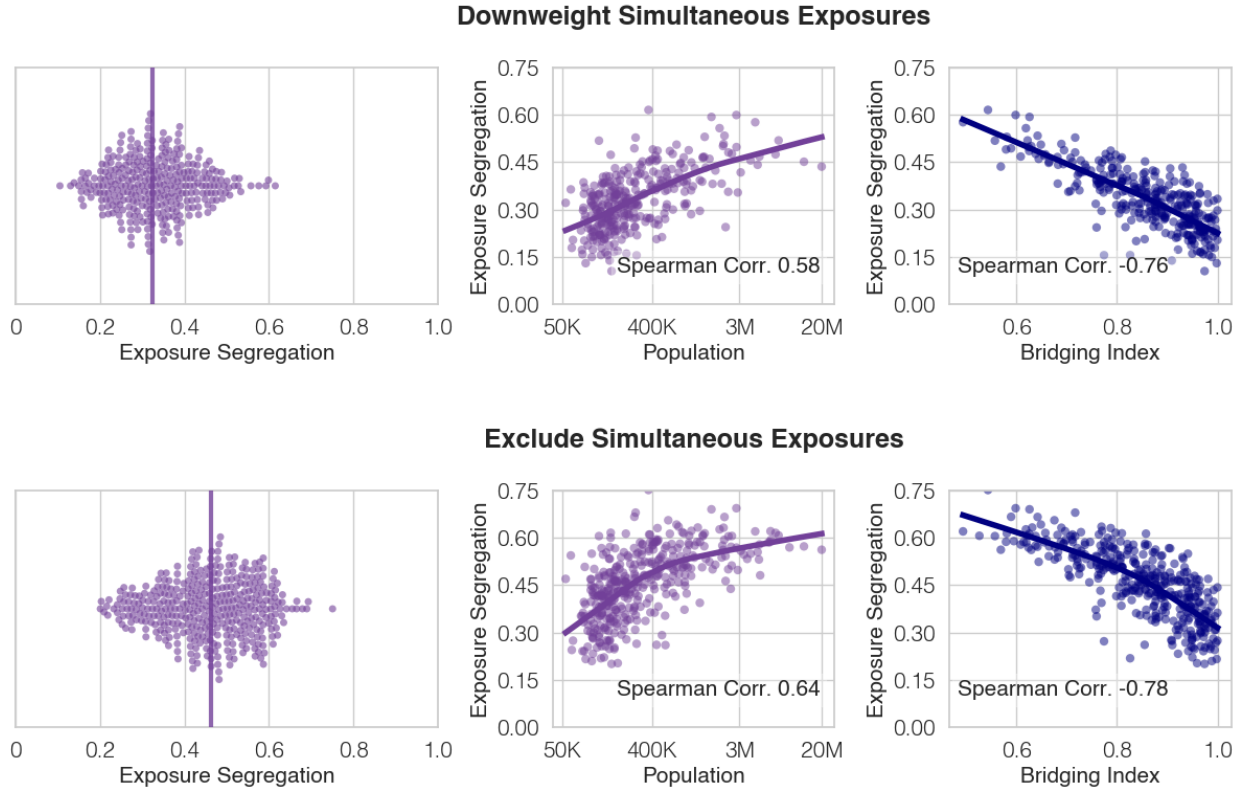

Supplementary Figure S9: Robustness of primary study findings to background density of exposures. We find that our primary study findings that, (1) large, dense cities are more segregated and (2) hub locations accessible to diverse individuals may mitigate segregation, are robust to the choice of whether to downweight or exclude simultaneous exposures in our exposure network. We conduct these robustness checks to ensure that our results are not overly influenced by background density of exposures[5], and that our results generalize to exposures in areas with low background density:

Exclude Simultaneous Exposures: For all exposures between two people (Person A and Person B), we exposures in which either person was simultaneously exposed to an additional individual (i.e. Person C was observed within 50 meters and 5 minutes of either Person A or Person B).

Downweight Simultaneous Exposures: For each exposure between Person A and Person B, we compute S the total number of additional people that Person A and Person B are simultaneously exposed to (within a 5 minute window of their pings). Exposures are then weighted by $min(25 - S, 0)$, i.e. exposure weight decreases as there are more simultaneous individuals exposed, and exposures with more than 25 people are excluded entirely

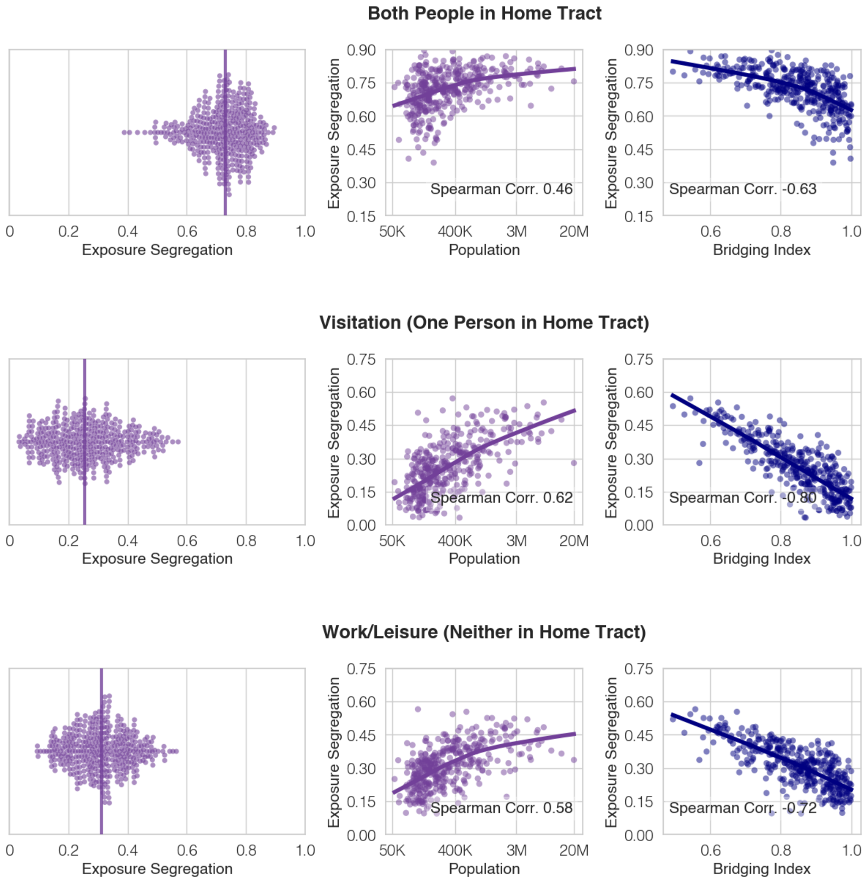

Supplementary Figure S10: Robustness of primary study findings to including only exposures in which both individuals, one individual, or neither individuals were located within their home tract at the time of exposure. We find that our primary study findings that, (1) large, dense cities are more segregated and (2) hub locations accessible to diverse individuals may mitigate segregation, are robust to filtering for a subset of exposures. We compare the results of:

Both Within Home Tract: We filter to include only exposures when both individuals are located within their home tract.

Visitation (One Within Home Tract): We filter to include only exposures which occur when one individual visits the home tract of the second individual.

Work/Leisure (Both Within Home Tract): We filter to include only exposures likely to take place in the context of work or leisure, by including only exposures which occurred when both individuals were located within their home tracts.

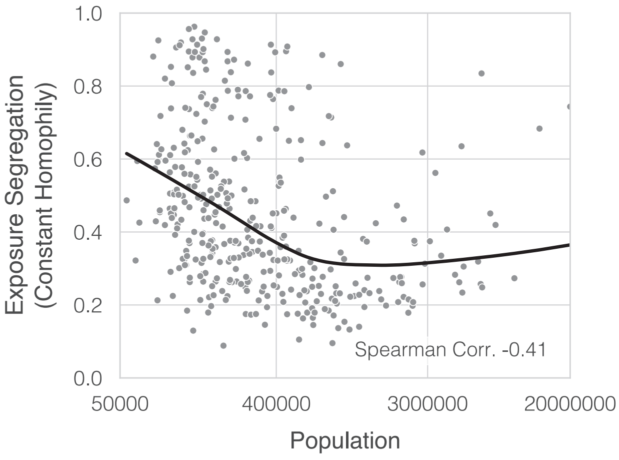

(a)

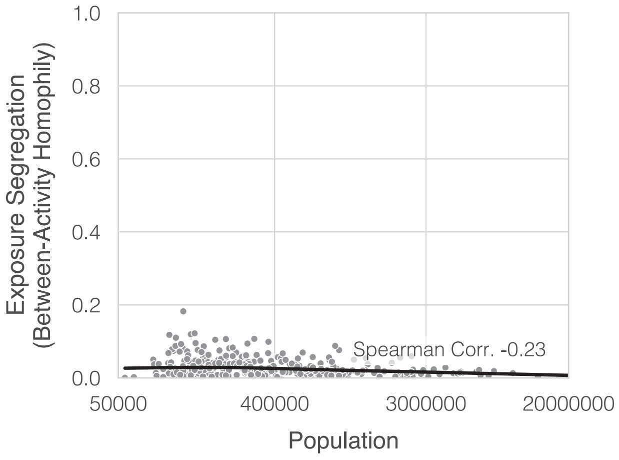

(b)

Supplementary Figure S11: Alternative homophily mechanisms do not explain segregation in large cities. We consider the possibility of two alternative hypotheses which may explain the trend towards high segregation in large cities
(a) *Constant Homophily*: i.e. individuals have the same proclivity for crossing paths with individuals of similar SES regardless of if they live in large or small cities, and it is instead change in distribution of socioeconomic status that drives segregation in large cities (e.g. in large cities there may be a greater supply of people in the same economic class available to cross paths with). We test this hypothesis via a null network model, in which which we preserve network nodes (individuals and their SES values) but randomize edges[6,7]. We randomly assign exposures between pairs of people, weighting the likelihood of exposure between people of similar SES higher according to a constant homophily function. Specifically, the probability of exposure ($p_{i,j}$) between two individuals of SES_i and SES_j is weighted by their similarity in SES, defined as the complement of the normalized Euclidean distance in SES: $p_{i,j} \propto Similarity(SES_i, SES_j) = 1 - \frac{|SES_i - SES_j|}{max(SES) - min(SES)}$. We choose 75 exposures per person such that the mean number of exposures per person is 150, which corresponds to Dunbar's number[8]. We find that under this null model, there is no positive association between exposure segregation and population size; in fact, larger cities are less segregated on average, as there is an increase in supply of diverse individuals in socioeconomic status in larger cities. These findings are also robust to a variety of null model specifications (Supplementary Figure S12).
(b) *Between Activity Homophily*: i.e. it is not the differentiation of individual venues that drives segregation, but rather that in large cities individuals choose different categories of activities which results in segregation (e.g. in small cities, there are less country clubs so everybody visits restaurants to socialize, whereas in large cities high-SES individuals segregate by spending a higher proportion of time in exclusive venues such as country clubs). We test this hypothesis via a configuration model [6,7], a prominent null network model in which node degree is preserved. Specifically, by applying a configuration model to reconfigure network edges for each leisure category separately, we preserve network nodes (individuals and their SES values) as well as the number of exposures they had in each category of POI (node degree), but randomize the specific venue in which each exposure occurred. For instance, if an individual crosses paths with 5 people inside of restaurants and 100 people inside of a fitness center, they will be randomly assigned to cross paths with 5 people from all of those who visited restaurants, and 100 people from all of those who visited fitness centers. This null model preserves between-activity homophily which results from activity choices (e.g. whether to visit a country club or restaurant), but erases within-activity homophily (e.g. individuals who visit any restaurant are equally likely to cross paths). We find that under this null model, there is no positive association between exposure segregation and population size; in fact, there is minimal segregation across all cities as variation between activity categories is insufficient to retain segregation. This is further supported by Supplementary Table S3, which shows relatively small differences in SES between participants in different categories of leisure activity (e.g. the lowest SES activity, limited service restaurants has a median visitor SES of $1,352, the highest SES activity, golf courses and country clubs has a median visitor SES of $1,648.4).

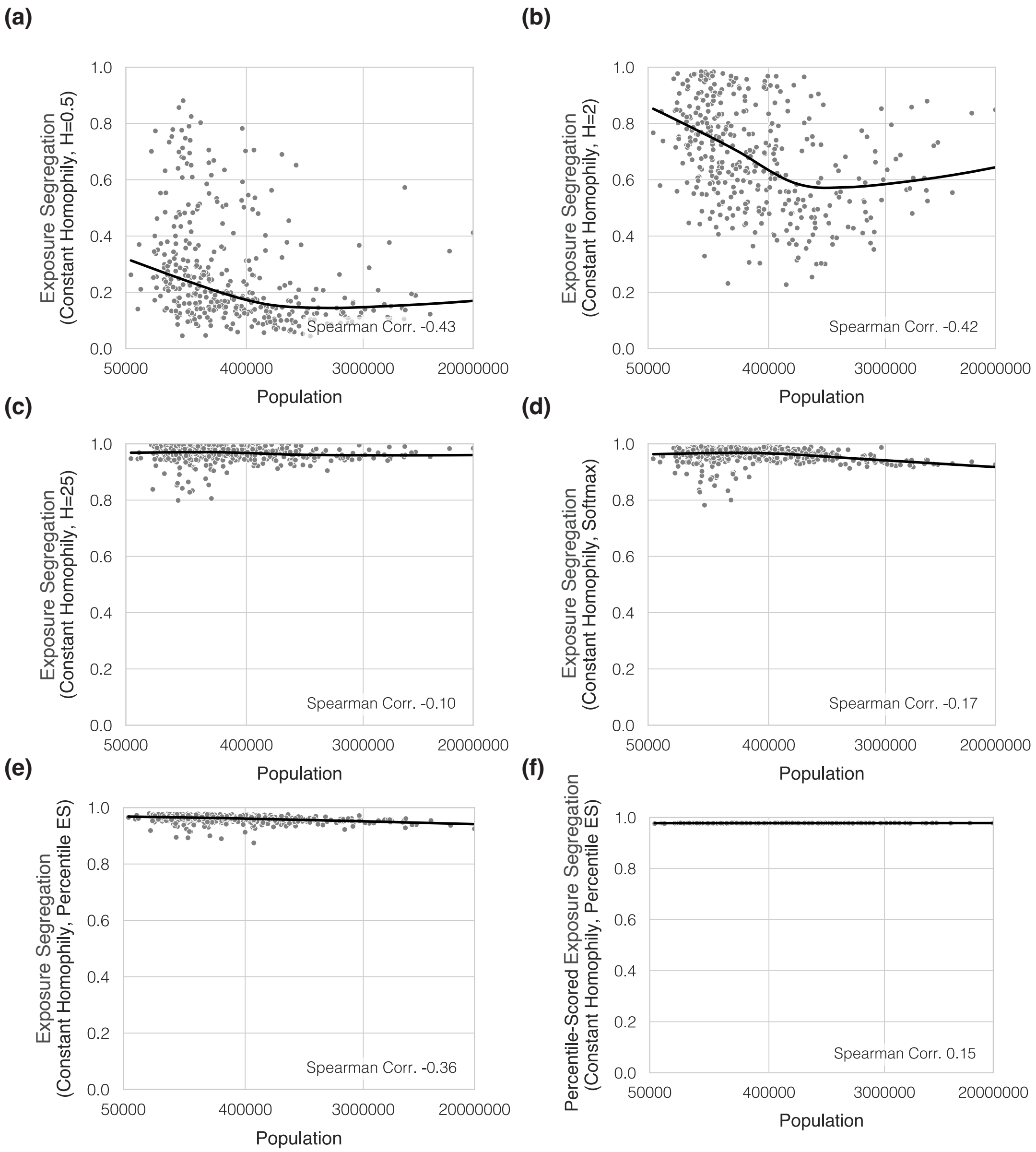

Supplementary Figure S12: Baseline Homophily null model results are robust to varying null model specifications. We re-run the analysis in Supplementary Figure S11a under a variety of null model specifications, and find that *in all cases there is no evidence to suggest that the Constant Homophily hypothesis explains the high segregation observed in large cities*. **(a-c)** We first consider varying the extent of homophily by adding a constant parameter H to the homophilous weighting of edges, to exponentially increase/decrease the extent of homophily in our null model for the probability of individuals i and j crossing paths: $p_{i,j} \propto Similarity(ES_i, ES_j)^H$. We find that regardless of if we **(a)** decrease homophily (H=0.5) or **(b-c)** increase homophily mildly (H=2) or strongly (H=25), there is no positive association between population size and segregation in our simulations. In fact, larger cities are less segregated on average, as there is an increase in supply of diverse individuals in socioeconomic status in larger cities. We also consider alternative null model specifications such as **(d)** a softmax homophily function $p_{i,j} \propto \frac{e^{Similarity(ES_i,ES_j)}}{\sum_{k=1}^{N} e^{Similarity(ES_i,ES_k)}}$, **(e)** applying the original null model to percentile-scored values socioeconomic status **(f)** applying the original null model to percentile-scored values socioeconomic status, and calculating Exposure Segregation using percentile-scored values socioeconomic status. This suggests that the high segregation in large cities is due to a change in resident behavior, facilitated by the built environment of large cities, and not an artifact the socioeconomic status distribution in large cities.

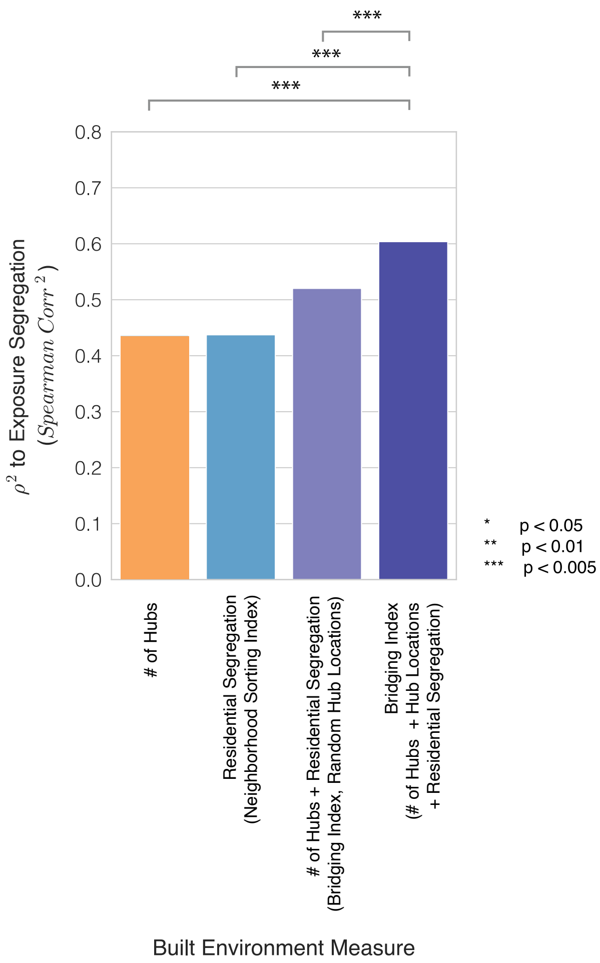

Supplementary Figure S13: Understanding why the bridging index explains exposure segregation. We show via an ablation study that hub locations, in addition to number of hubs and residential segregation, contributes to the explanatory power of the bridging index. As illustrated in Extended Data Figure 8, the bridging index captures three factors of built environment: (1) locations of hubs (2) number of hubs and (3) residential segregation. In this analysis, we aim to disentangle how these three factors contribute to the ability of the bridging index to explain exposure segregation (as measured by ρ^2, the squared Spearman correlation with exposure segregation). We find that number of hubs (orange, $\rho^2 = 0.436$) and residential segregation (blue, $\rho^2 = 0.437$) are each correlated with exposure segregation. To measure the combined explanatory power of these two factors within the bridging index, independent of hub locations, we conduct an ablation study in which we calculate bridging index for each MSA, using the actual home location data and number of hubs for each MSA, but randomize hub locations (light purple, $\rho^2 = 0.523$). For each MSA, we estimate this value over 1000 random trials. We find that calculating the bridging index using randomized hub locations is a significantly weaker predictor ($p<0.001$; Two-sided Steiger's's Z-test; Methods 'Hypothesis testing') compared to bridging index values computed using actual hub locations (dark purple, $\rho^2 = 0.604$). This demonstrates that hub locations contribute to the explanatory power of the bridging index, i.e. it explains exposure segregation because it captures the extent to which the locations of hubs in different cities facilitate the exposure of diverse individuals.

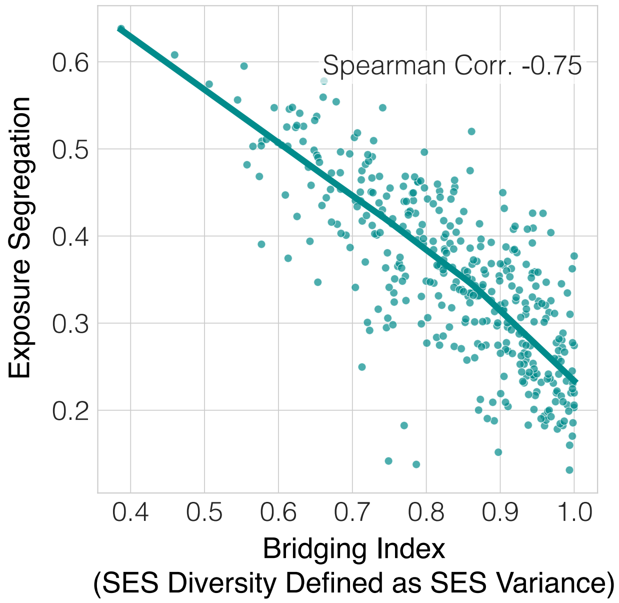

Supplementary Figure S14: Robustness of the bridging index to definition of SES diversity. We calculate a version of bridging index which uses variance in to operationalize economic diversity: $Bridging\ Index = \frac{\sum_{i=1}^{K} |\mathcal{C}_i| \cdot Var(\mathcal{C}_i)}{|\mathcal{V}_{MSA}| \cdot Var(\mathcal{V}_{MSA})}$. This variant of the bridging index explains exposure segregation comparably (Spearman Corr. -0.75 vs. -0.78, both N=382, both p$< 10^{-4}$; Two-sided Student's t-tests; Methods 'Hypothesis testing') to our primary measure of the bridging index which uses Gini index to operationalize economic diversity. Thus, we find that the ability of the bridging index to explain exposure segregation is robust to the definition of SES diversity.

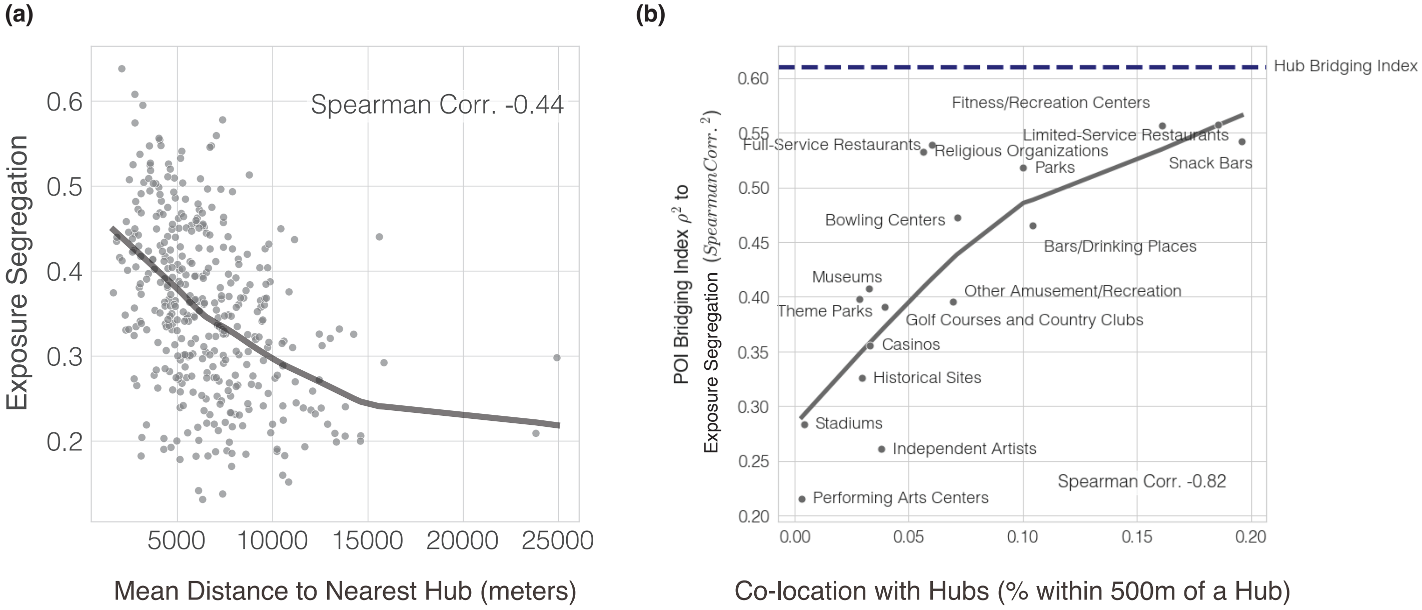

Supplementary Figure S15: We consider alternative processes through which built environment may mitigate exposure segregation. **(a)** Following the inverse relationship between POI localization and segregation established in Extended Data Figure 3a, we consider whether the de-localization of hubs alone can provide an alternative to the bridging index. We compute mean distance to nearest hub for each MSA, which is the same measure in Extended Data Figure 3a but calculated for hubs. We find that while hub localization is inversely correlated with exposure segregation (Spearman Corr -0.44, N=382, p$< 10^{-4}$; Two-sided Student's t-test; Methods 'Hypothesis testing'), this correlation is significantly less ($p < 10^{-4}$; Two-sided Steiger's's Z-test; Methods 'Hypothesis testing') than the correlation between bridging index and exposure segregation (Spearman Corr -0.78, N=382, p$< 10^{-4}$; Two-sided Student's t-test; Methods 'Hypothesis testing'). This suggests that hub bridging, as quantified by the bridging index may be a more promising direction to investigate as a potential mitigator of segregation. **(b)** We also consider whether fine-grained POIs may function as bridges between diverse individuals. For each of the fine-grained leisure POI categories in Figure 2c, we calculate a bridging index across all MSAs (using the same procedure to calculate the bridging index as shown in Extended Data Figure 7, except using fine-grained POI locations instead of hub locations). For instance, to calculate the bridging index for restaurants, we cluster all homes by the nearest restaurant location, and then calculate: $(Restaurant)\, Bridging\, Index = \frac{\sum_{i=1}^{K} |\mathcal{R}_i| \cdot Gini\ Index(\mathcal{R}_i)}{|\mathcal{V}_{MSA}| \cdot Gini\ Index(\mathcal{V}_{MSA})}$. After calculating the bridging index for all fine-grained POI categories and for each of the 382 MSAs, we then measure the correlation between each bridging index and exposure segregation across all MSAs (as measured by ρ^2, the squared Spearman correlation). We find that the bridging index for hubs provides a stronger correlation ($\rho^2 = 0.604$, horizontal line), than all other bridging indices which are plotted as points on the scatter-plot in **(b)**. Further, we find that POI categories which are often located inside or near hubs (co-location, X-axis) have bridging indices which are stronger predictors of exposure segregation (e.g. for fitness/recreation centers, snack bars etc.). The high correlation between (Spearman Correlation -0.82, N=17, $p < 0.001$; Two-sided Student's t-test; Methods 'Hypothesis testing') between co-location of POIs and bridging index predictive ability demonstrates asymptotic convergence between all other predictive bridging index metrics and our primary bridging index measure. This further suggests that bridging of hubs should be the primary metric of interest for mitigators of segregation, because other bridging indexes computed for fine-grained POI locations are at best proxies for the bridging index which leverages higher-level hub locations. Supplementary Figures S16-S18 illustrate the frequent co-location between hubs and other fine-grained POIs.

a) Town & County Village, Palo Alto, California

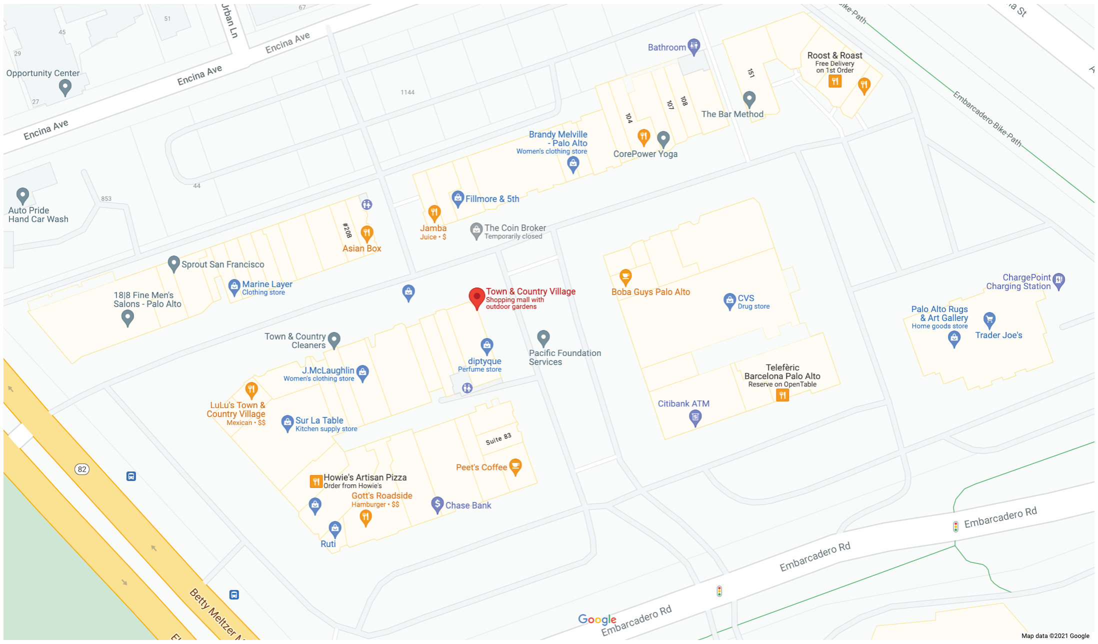

b) Faneuil Hall Marketplace, Boston, MA

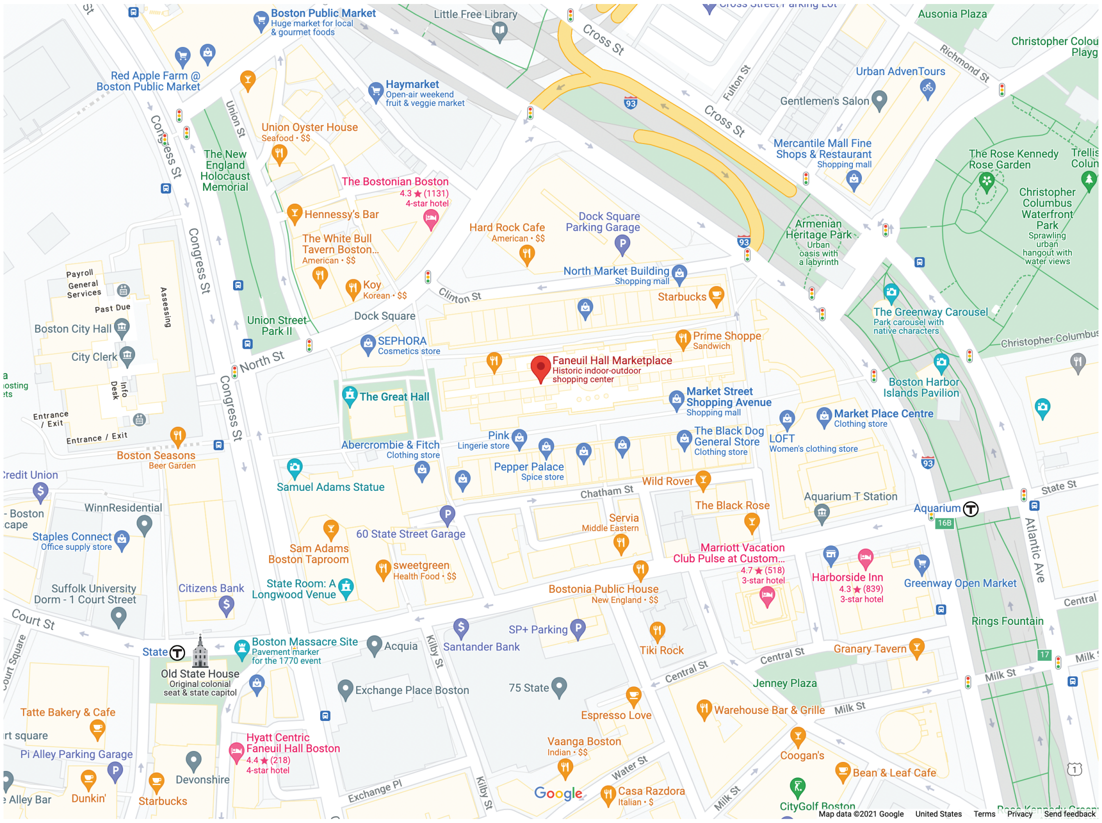

Supplementary Figure S16: Examples of hubs in coastal cities of (a) San Francisco Bay Area and (b) Boston, MA. Hubs frequently contain a diverse assortment of POIs including restaurants, fitness centers/gyms, grocery stores, etc. and are also frequently hubs around which other POIs are located nearby.

a) Cross Creek Mall and Surrounding Area, Fayetteville, NC

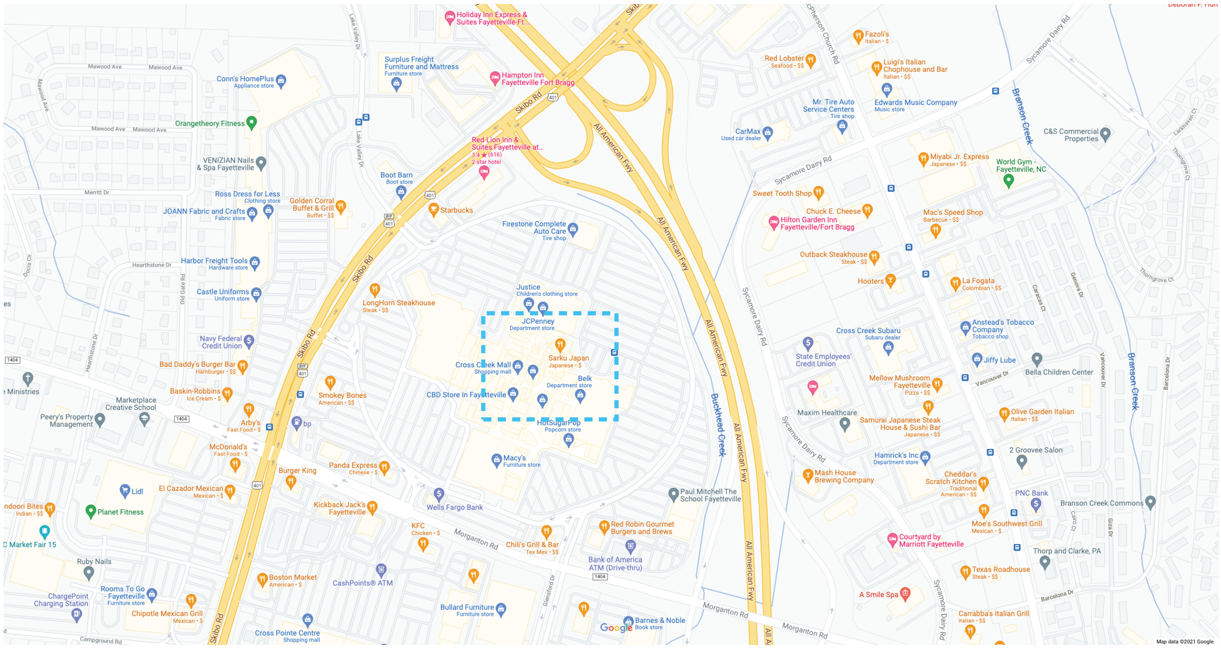

b) [Zoomed In] Cross Creek Mall, Fayetteville, NC

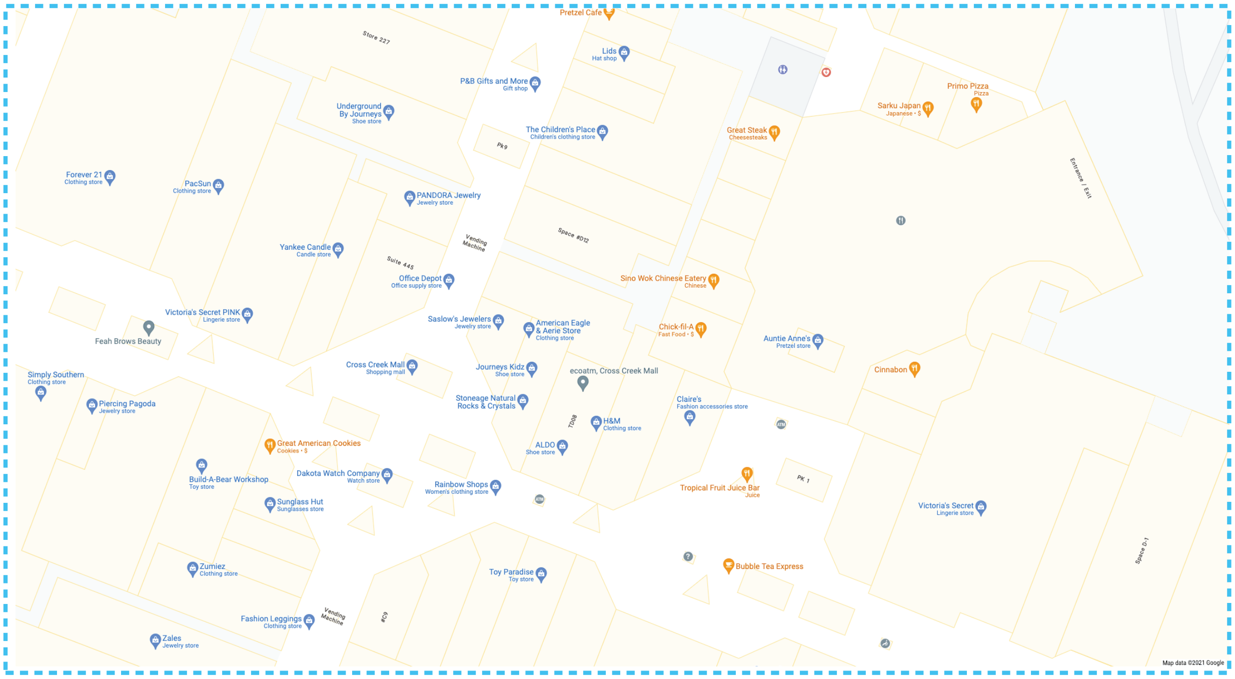

Supplementary Figure S17: Example of a major hubs in Fayetteville, NC (a) zoomed-out view of hub and surrounding co-located POIs (b) zoomed-in view of the hub core and businesses contained inside. We find that in Fayetteville, a city with a high bridging index, large hubs contain a variety of POIs which cater to diverse individuals of both high and low-ES.

a) The Shoppes at Eastchase, Montgomery, AL

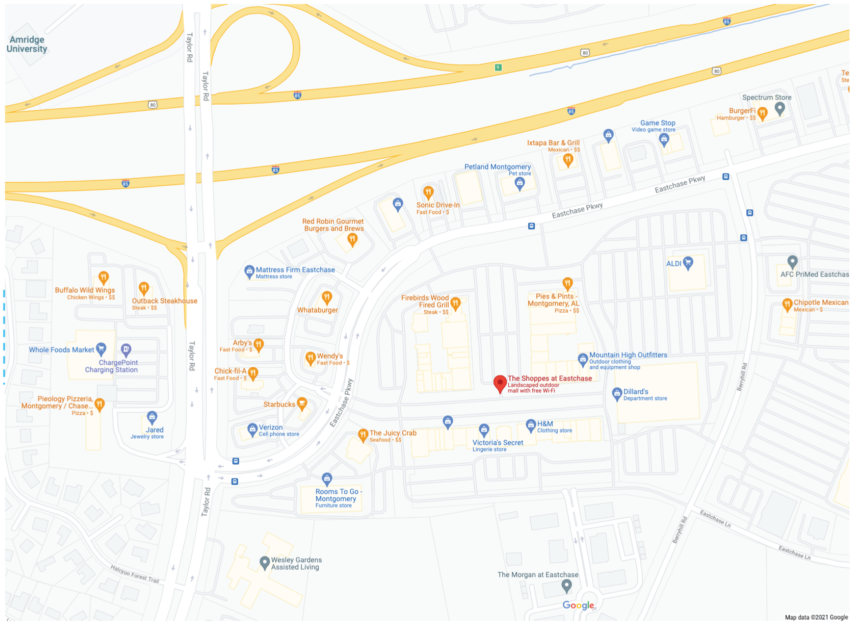

b) Midtown Plaza, Montgomery, AL

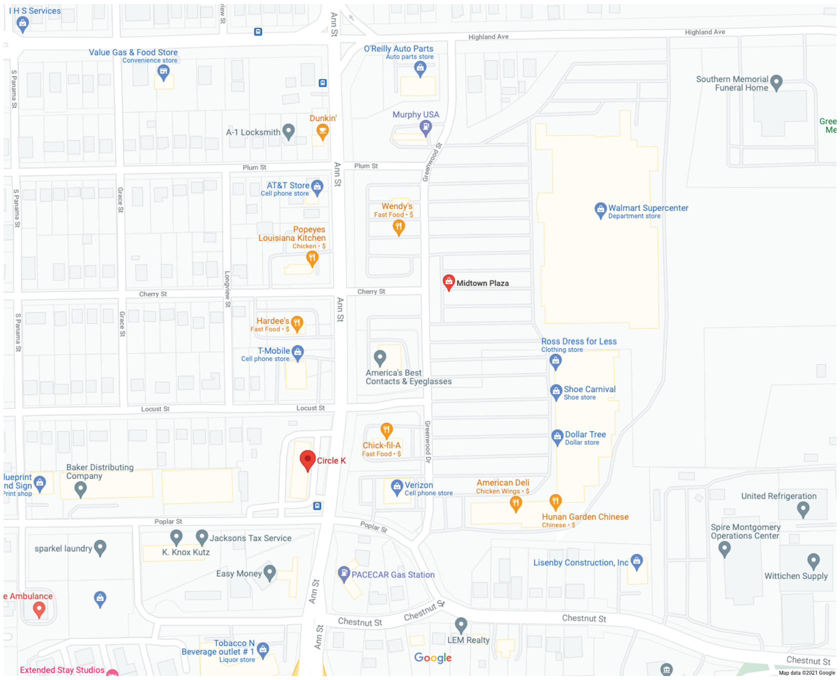

Supplementary Figure S18: Examples two hubs in Montgomery, AL which have visitors of predominantly (a) high socioeconomic status (b) low socioeconomic status. We find that in Montgomery, AL a city with a low bridging index, smaller hubs exist which contain POIs which cater to a narrow band of individuals in a specific economic stratum. For instance, we find that the nearby grocery store **(a)** is a Whole Foods Market in the high-SES hub, in contrast to the **(b)** Walmart Supercenter in the low-SES hub.

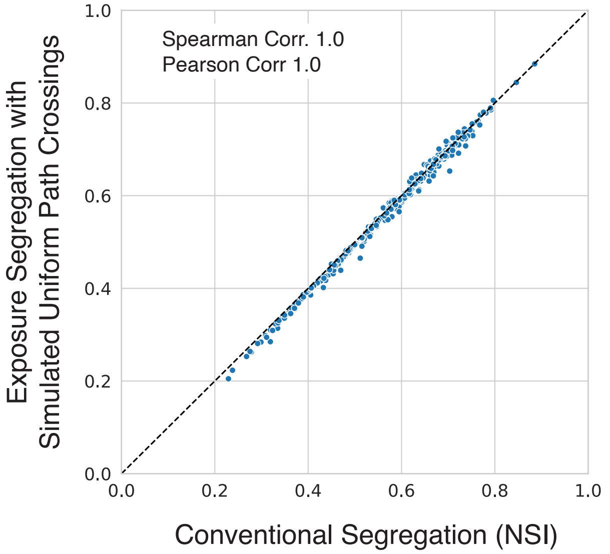

Supplementary Figure S19: Our model with simulated uniform within-tract crossings is equivalent to the conventional neighborhood sorting index. Each point is a Metropolitan Statistical Area (MSA). The y-axis shows the exposure segregation estimate from the mixed model with a simulated path crossing between every person in a tract (in our dataset). The x-axis shows the correlation between a person's SES and the average SES of people in their tract, which is the neighborhood sorting index. As these measures are equivalent, Spearman Corr = 1.0 and Pearson Corr. = 1.0.

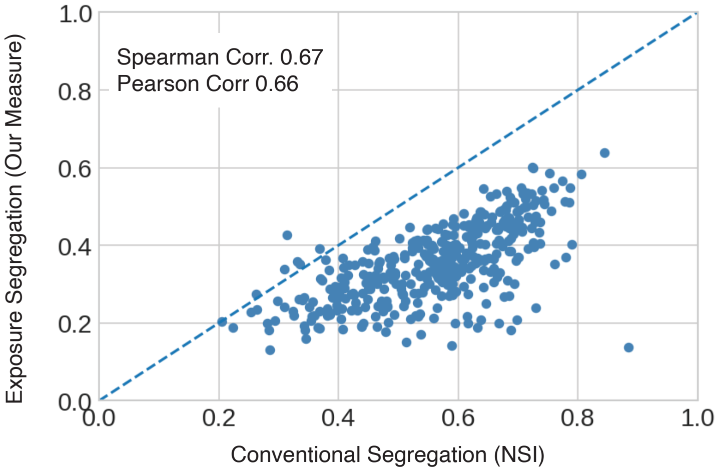

Supplementary Figure S20: Our segregation measure versus a conventional residential segregation measure, neighborhood sorting index. Each point is a Metropolitan Statistical Area (MSA). Regardless of whether we compare the numerical segregation values (Pearson Correlation 0.67) or the MSA ranking (Spearman Correlation 0.66), only moderate correlation indicates that our measure is different in kind from residential segregation as measured conventionally by the neighborhood sorting index.

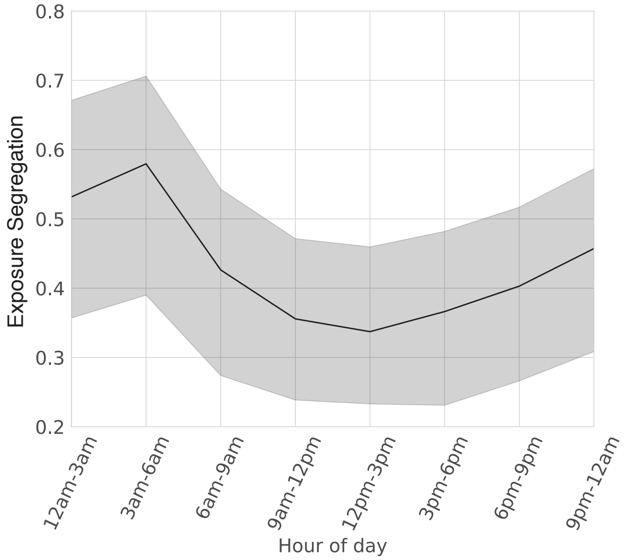

Supplementary Figure S21: Segregation decomposed by time. Our fine-grained exposure network allows us to decompose our overall exposure segregation into estimates of segregation during different hours of the day (Methods 'Decomposing segregation by time'), by filtering for exposures that occurred within a specific hour. Y axis shows median exposure segregation across all N=382 MSAs; shaded bands indicate 10th and 90th percentiles. In Supplementary Figure S21, we partition estimates of segregation by 3 hour windows to illustrate how segregation varies throughout the day (see Supplementary Information). We observe that segregation increases by 61% between the afternoon and early morning hours. Segregation is lowest during commute and work hours, indicating higher levels of exposure with people of different SES while at work or otherwise away from home. Segregation is higher during nighttime hours. This is driven by individuals returning to their home neighborhoods, which are more homogeneous in socioeconomic status, as we well as mechanically a result of SES being is defined by rent value, such that people who live in the same household will have the same SES (and thus will be highly segregated).

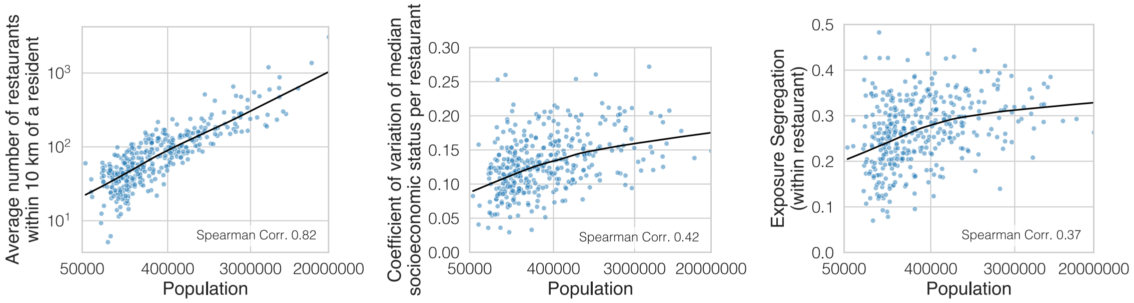

(a) Full-Service Restaurants

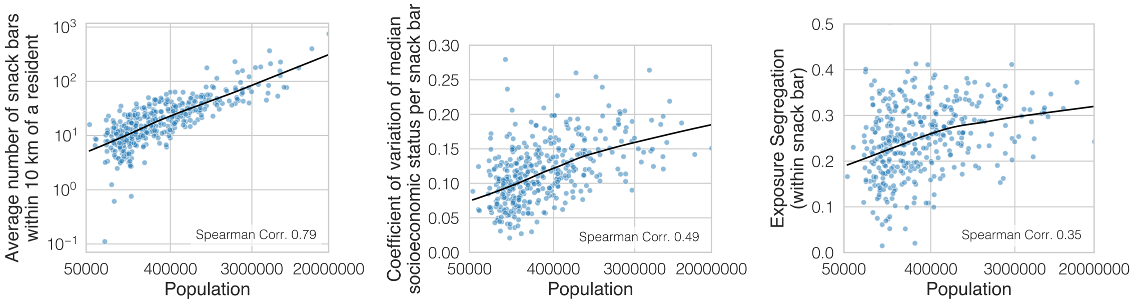

(b) Snack Bars

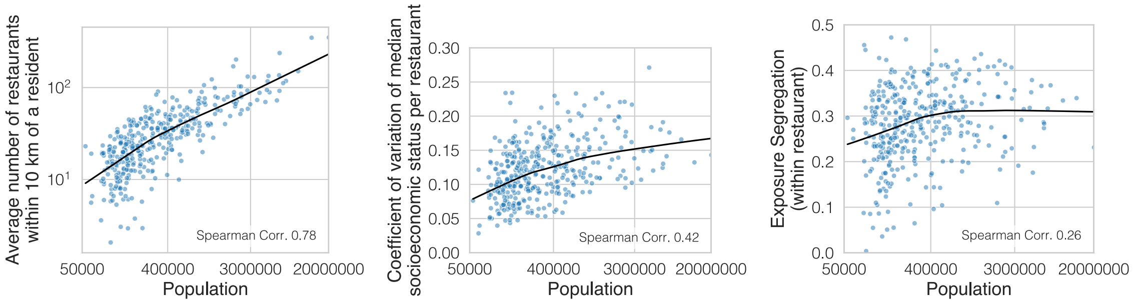

(c) Limited-Service Restaurants

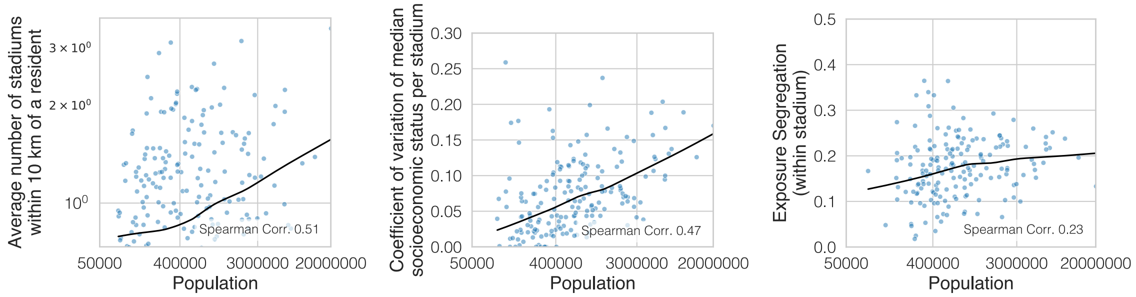

(d) Stadiums

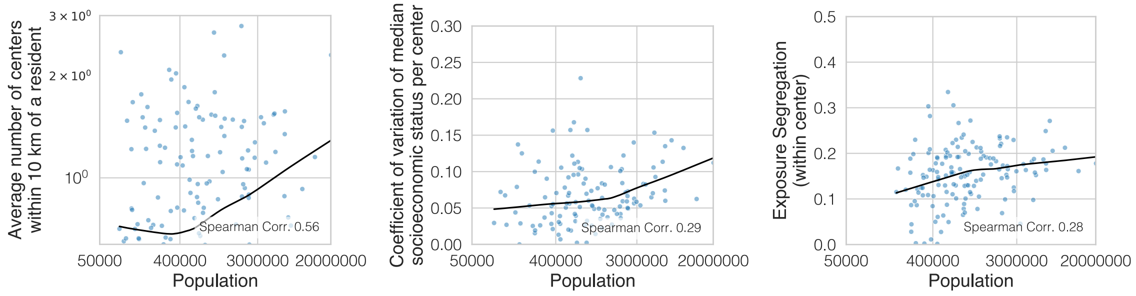

(e) Performing Arts Centers

Supplementary Figure S22: Across many activities, POIs in large cities are more differentiated and consequently more segregated. This figure shows that the trend towards more options, increased differentiation, and consequently higher segregation is consistent across many prominent POI categories. Here we find similar results for the 5 most frequently visited fine-grained Safegraph place features. The analyses for full-service correspond to Figures 2c-e, and we additionally show the same trend for snack bars, limited-service restaurants, stadiums, and performing arts centers (ranked 2-5 after full-service restaurants in terms of most frequently visited POIs among Safegraph places). Across the board, large, densely populated metropolitan areas are associated with increased options and economic differentiation of POIs, which may facilitate higher self-segregation.

	Dependent variable:				Exposure Segregation	
	(1)	(2)	(3)	(4)	(5)	(6)
Intercept	0.355***	0.355***	0.355***	0.356***	0.355***	0.355***
	(0.005)	(0.004)	(0.004)	(0.004)	(0.003)	(0.003)
Population Density	0.039***		0.024***	0.022***	0.017***	0.017***
	(0.005)		(0.004)	(0.004)	(0.004)	(0.004)
Gini Index (Estimated Rent)		0.064***	0.058***	0.059***	0.049***	0.050***
		(0.004)	(0.004)	(0.004)	(0.003)	(0.003)
Political Alignment (% Democrat in 2016 Election)				0.009*		0.006
				(0.005)		(0.004)
Racial Demographics (% non-Hispanic White)				-0.005		0.003
				(0.004)		(0.003)
Mean SES (Estimated Rent)				-0.009*		-0.003
				(0.005)		(0.004)
Walkability (Walkscore)					0.002	0.001
					(0.003)	(0.004)
Commutability (% Commute to Work)					-0.012***	-0.013***
					(0.004)	(0.004)
Conventional Segregation (Neighborhood Sorting Index)					0.048***	0.047***
					(0.003)	(0.003)
Observations	382	382	382	376	382	376
R^2	0.151	0.419	0.475	0.490	0.682	0.680
Adjusted R^2	0.149	0.417	0.472	0.483	0.678	0.673

*p<0.1; **p<0.05; ***p<0.01

Supplementary Table S7: Population density is significantly associated with exposure segregation, after controlling for MSA income inequality (Gini index), political alignment (% Democrat in 2016 election), racial demographics (% non-Hispanic White), mean SES, walkability (Walkscore[9]), commutability (% of residents commuting to work), and residential segregation (neighborhood sorting index). This table is from an analogous regression to the regression shown in Extended Data Table 1, using population density instead of population size (we look at each separately due to co-linearity between population size and density). Here we show the coefficients (after normalizing via z-scoring to have mean 0 and variance 1) from the primary specifications estimating the effect of population density on exposure segregation across all MSAs. Standard errors are displayed in parentheses beneath each regression coefficient (*p < 0.1; **p < 0.05; ***p < 0.01; Two-sided Student's t-test; Methods 'Hypothesis testing'). Columns (1-5) are models specified with different subsets of covariates; Column 6 shows model specification with all covariates. Differences between sample size in models is due to missing data for several covariates in a small number of MSAs (Walkscores were not available for all MSAs).

Percentile	# of Exposures	Percentile	# of Exposures
0.0	1.0	51.0	146.0
1.0	2.0	52.0	152.0
2.0	4.0	53.0	157.0
3.0	5.0	54.0	163.0
4.0	7.0	55.0	168.0
5.0	8.0	56.0	174.0
6.0	10.0	57.0	181.0
7.0	12.0	58.0	187.0
8.0	14.0	59.0	194.0
9.0	16.0	60.0	201.0
10.0	17.0	61.0	208.0
11.0	19.0	62.0	215.0
12.0	21.0	63.0	223.0
13.0	23.0	64.0	231.0
14.0	25.0	65.0	240.0
15.0	28.0	66.0	249.0
16.0	30.0	67.0	258.0
17.0	32.0	68.0	268.0
18.0	34.0	69.0	278.0
19.0	36.0	70.0	289.0
20.0	39.0	71.0	300.0
21.0	41.0	72.0	312.0
22.0	44.0	73.0	325.0
23.0	46.0	74.0	338.0
24.0	49.0	75.0	353.0
25.0	51.0	76.0	368.0
26.0	54.0	77.0	384.0
27.0	57.0	78.0	402.0
28.0	59.0	79.0	420.0
29.0	62.0	80.0	440.0
30.0	65.0	81.0	462.0
31.0	68.0	82.0	485.0
32.0	71.0	83.0	511.0
33.0	74.0	84.0	539.0
34.0	77.0	85.0	569.0
35.0	81.0	86.0	603.0
36.0	84.0	87.0	640.0
37.0	88.0	88.0	683.0
38.0	91.0	89.0	731.0
39.0	95.0	90.0	786.0
40.0	98.0	91.0	851.0
41.0	102.0	92.0	927.0
42.0	106.0	93.0	1018.0
43.0	110.0	94.0	1133.0
44.0	114.0	95.0	1280.0
45.0	118.0	96.0	1482.0
46.0	123.0	97.0	1781.0
47.0	127.0	98.0	2291.0
48.0	132.0	99.0	3480.0
49.0	136.0	100.0	194243.0
50.0	141.0		

Supplementary Table S8: Distribution of number of exposures for all individuals residing in 382 Metropolitan Statistical Areas (MSAs). The median individual had 141 exposures overall. 8,609,406 individuals reside in a Metropolitan Statistical Area (90% of the overall 9,567,559 individuals in our study). The remaining 958,153 users live outside of MSAs, influencing the exposure segregation of an MSA by coming into contact with MSA residents.

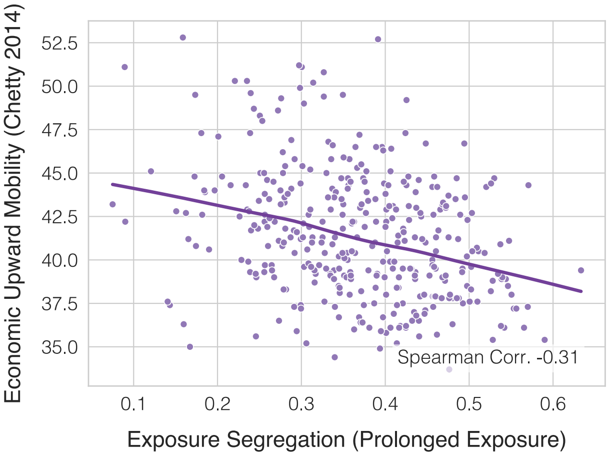

(a)

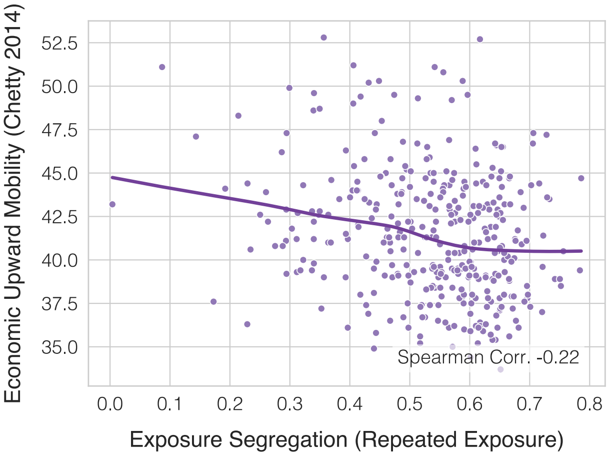

(b)

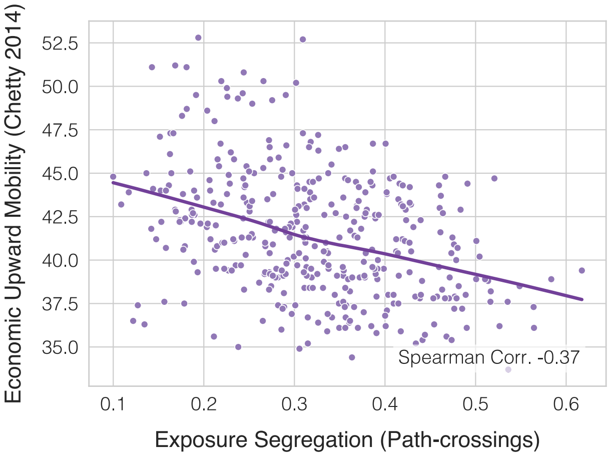

(c)

Supplementary Figure S23: We measure the external validity of alternative measures in which the strictness of our exposure definition is varied. We compute exposure segregation using only prolonged exposure of 3+ consecutive intervals of exposure on the same day, repeated exposure of 3+ consecutive intervals of exposure on different days, and path crossings (i.e. pairs of users that had only one instance of being within proximity of each other). We find all measures of exposure segregation correlate to (absolute) upward economic mobility (Spearman Correlation -0.31, $N = 365$, $p < 10^{-4}$; Two-sided Student's t-test; Methods 'Hypothesis testing'), (Spearman Correlation -0.22, $N = 364$, $p < 10^{-4}$; Two-sided Student's t-test; Methods 'Hypothesis testing'), and (Spearman Correlation -0.37, $N = 382$, $p < 10^{-4}$; Two-sided Student's t-test; Methods 'Hypothesis testing') respectively. Associations are significant even for the weakest definition of exposure (path-crossings), which may reflect the strength of weak ties in shaping upward economic mobility[10,11].

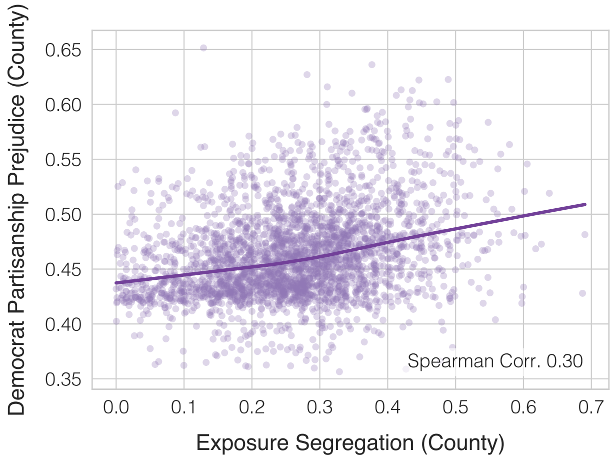

(a)

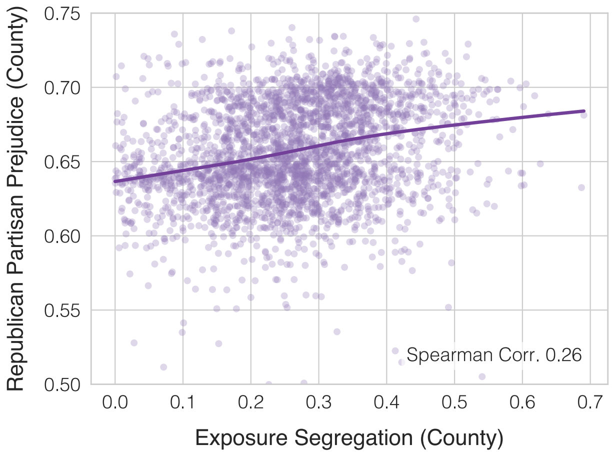

(b)

Supplementary Figure S24: Exposure segregation predicts political polarization outcomes. We measure the external validity of our definition of exposure segregation, by linking our measure to outcomes from a large-scale survey of political polarization[12]. We find that county-level exposure segregation correlates to political prejudice among both (a) Democrats (Spearman Correlation 0.30, $N = 2828$, $p < 10^{-4}$; Two-sided Student's t-test; Methods 'Hypothesis testing') and (b) Republicans (Spearman Correlation 0.26, $N = 2828$, $p < 10^{-4}$; Two-sided Student's t-test; Methods 'Hypothesis testing'). These findings suggest that exposure to diverse others may lead to increased tolerance of inter-group differences, following following prior work[2].

.

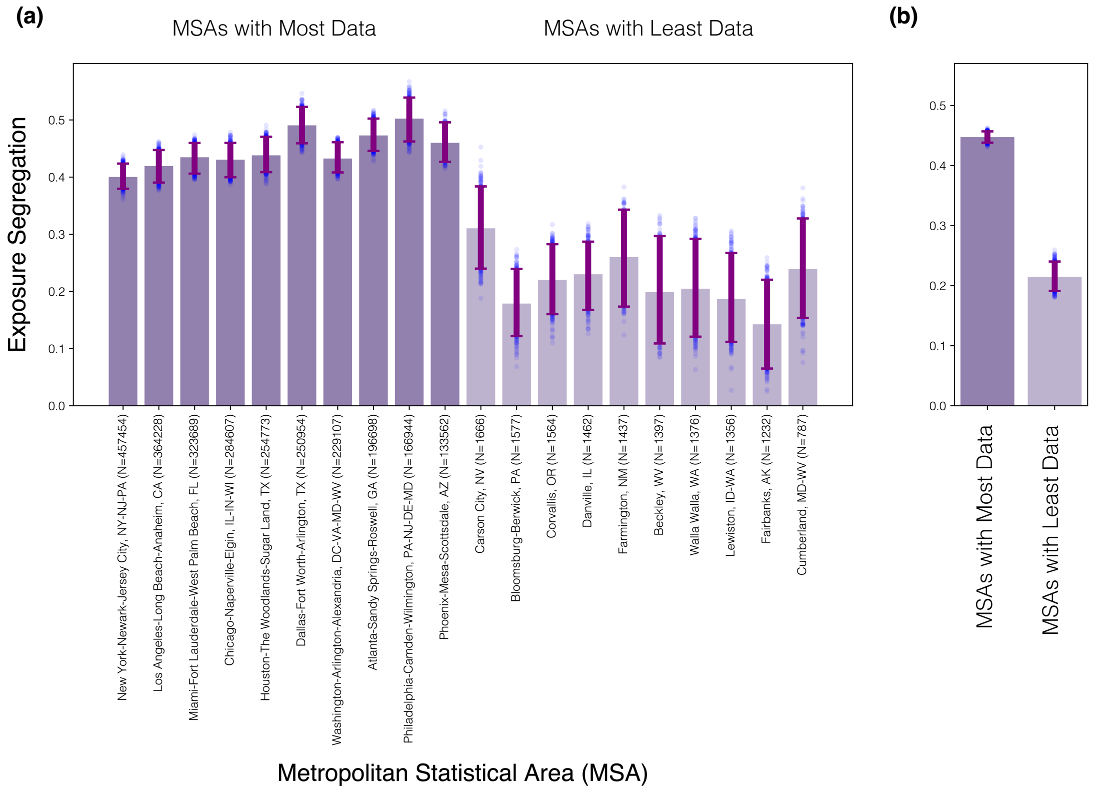

Supplementary Figure S25: Precision of exposure segregation estimates. We quantify precision of our exposure segregation estimates by computing 95% bootstrap confidence intervals. Nonparametric bootstrap confidence intervals indicate resampling nodes in the network with replacement and recomputing exposure segregation in each MSA using our mixed model (with N=1000 replications). Blue dots indicate individual replications outside of the 95% confidence interval. **(a)** To illustrate the precision of our estimates, we show the 10 MSAs with the most individuals in our dataset compared to the 10 MSAs with the least individuals in our dataset. Bar height corresponds to exposure segregation estimates on the full dataset. Nearly all pairs of small and large MSAs have non-overlapping confidence intervals. Even in smallest cities with little data, we can estimate exposure segregation with sufficient confidence to be able to compare to larger cities, finding that larger cities are more segregated. **(b)** We compare the mean exposure segregation of large and small MSAs and find that the mean of the top 10 is higher across all 1000 replicates ($p < 0.001$; Two-sided bootstraps; Methods 'Hypothesis testing').

.

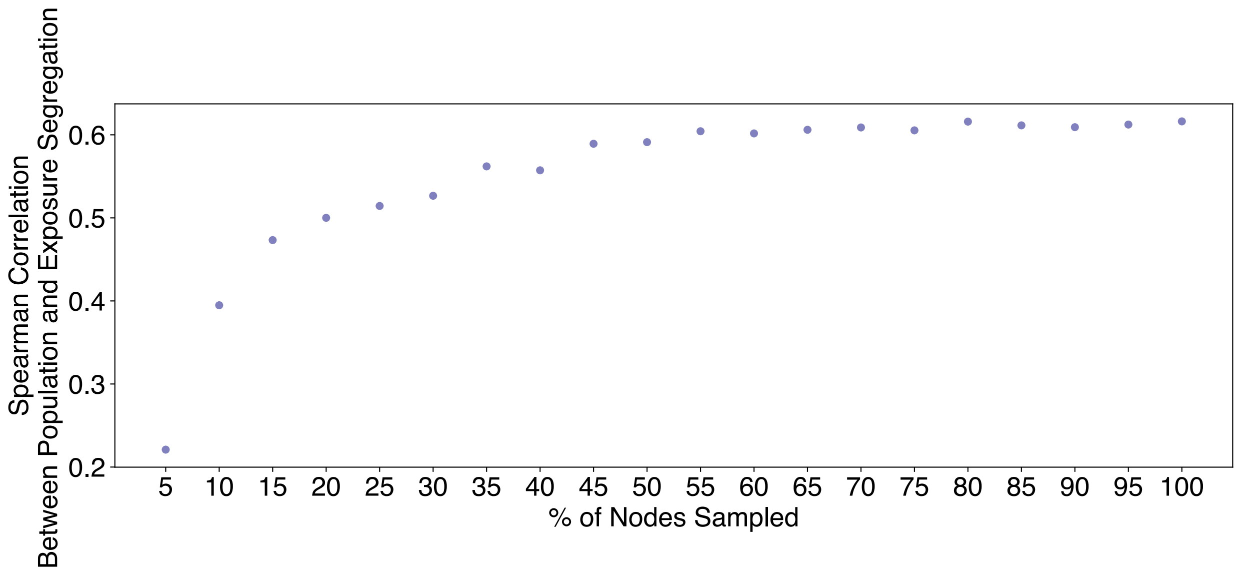

(a)

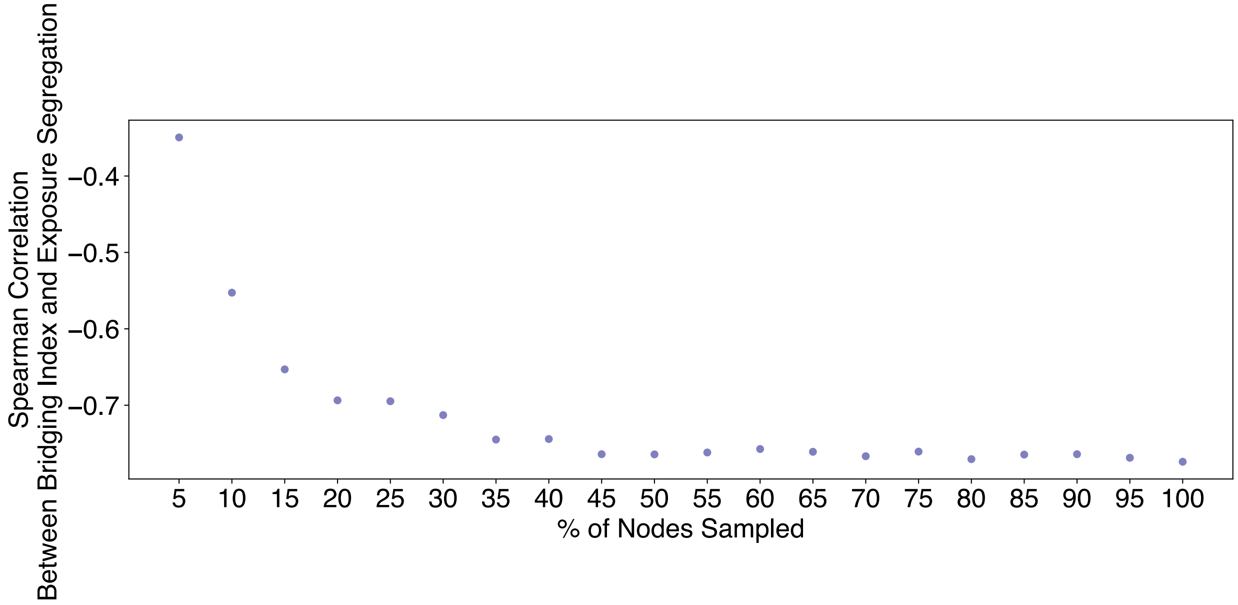

(b)

Supplementary Figure S26: Robustness of primary findings to network size. We further assess whether our key findings remain robust to smaller and sparser networks than the one we have amassed. To this end we downsample the nodes in the network and recompute our key findings using only the downsampled network. Statistically significant correlations ($p < 0.0001$; Two-sided bootstrap; Methods 'Hypothesis testing') across all samples, as well as minimal variation in Spearman Corr. (0.60 to 0.62 for population size, and -0.75 to -0.78 for bridging index) after over 50% of the network is sampled reveals that we have abundant data to support our claims. Diminishing returns are to be expected from amassing further network data.

Percentile	# of Exposures	Percentile	# of Exposures
0.0	1.00	51.0	7.10
1.0	1.00	52.0	7.27
2.0	1.29	53.0	7.44
3.0	1.50	54.0	7.62
4.0	1.60	55.0	7.81
5.0	1.73	56.0	8.00
6.0	1.86	57.0	8.20
7.0	2.00	58.0	8.40
8.0	2.00	59.0	8.62
9.0	2.17	60.0	8.83
10.0	2.27	61.0	9.06
11.0	2.38	62.0	9.30
12.0	2.50	63.0	9.54
13.0	2.57	64.0	9.80
14.0	2.67	65.0	10.06
15.0	2.77	66.0	10.33
16.0	2.88	67.0	10.62
17.0	3.00	68.0	10.93
18.0	3.05	69.0	11.25
19.0	3.17	70.0	11.57
20.0	3.26	71.0	11.93
21.0	3.36	72.0	12.29
22.0	3.47	73.0	12.68
23.0	3.57	74.0	13.09
24.0	3.67	75.0	13.52
25.0	3.77	76.0	14.00
26.0	3.88	77.0	14.49
27.0	4.00	78.0	15.00
28.0	4.08	79.0	15.58
29.0	4.19	80.0	16.19
30.0	4.30	81.0	16.83
31.0	4.41	82.0	17.55
32.0	4.52	83.0	18.32
33.0	4.64	84.0	19.17
34.0	4.75	85.0	20.09
35.0	4.87	86.0	21.12
36.0	5.00	87.0	22.27
37.0	5.11	88.0	23.57
38.0	5.23	89.0	25.04
39.0	5.36	90.0	26.74
40.0	5.50	91.0	28.71
41.0	5.62	92.0	31.06
42.0	5.75	93.0	33.92
43.0	5.89	94.0	37.48
44.0	6.00	95.0	42.10
45.0	6.17	96.0	48.46
46.0	6.31	97.0	57.88
47.0	6.46	98.0	74.00
48.0	6.62	99.0	112.19
49.0	6.77	100.0	5351.00
50.0	6.94		

Supplementary Table S9: Distribution of average number of exposures (per active day) for all individuals residing in 382 Metropolitan Statistical Areas (MSAs). The median individual had 6.94 exposures on the average day of activity. 8,609,406 individuals reside in a Metropolitan Statistical Area (90% of the overall 9,567,559 individuals in our study). The remaining 958,153 users live outside of MSAs, influencing the exposure segregation of an MSA by coming into contact with MSA residents. Activity is defined as one or more exposures occurring on a given day. For details on activity over time, see Supplementary Figure S27.

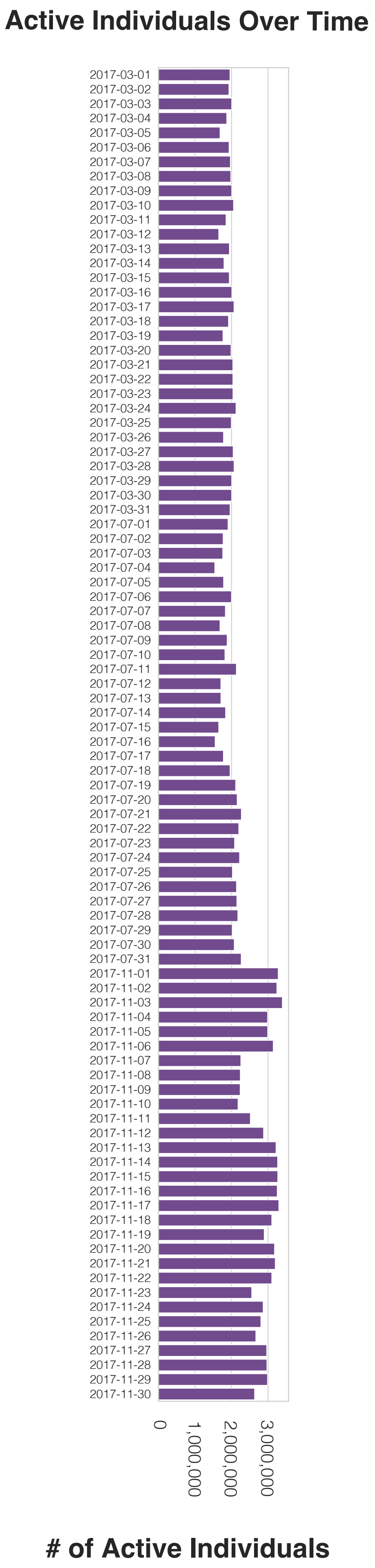

Supplementary Figure S27: Active individuals over time. Number of active individuals (i.e. nodes in the network) over the study observation period. Activity is defined as one or more exposures occurring on a given day.

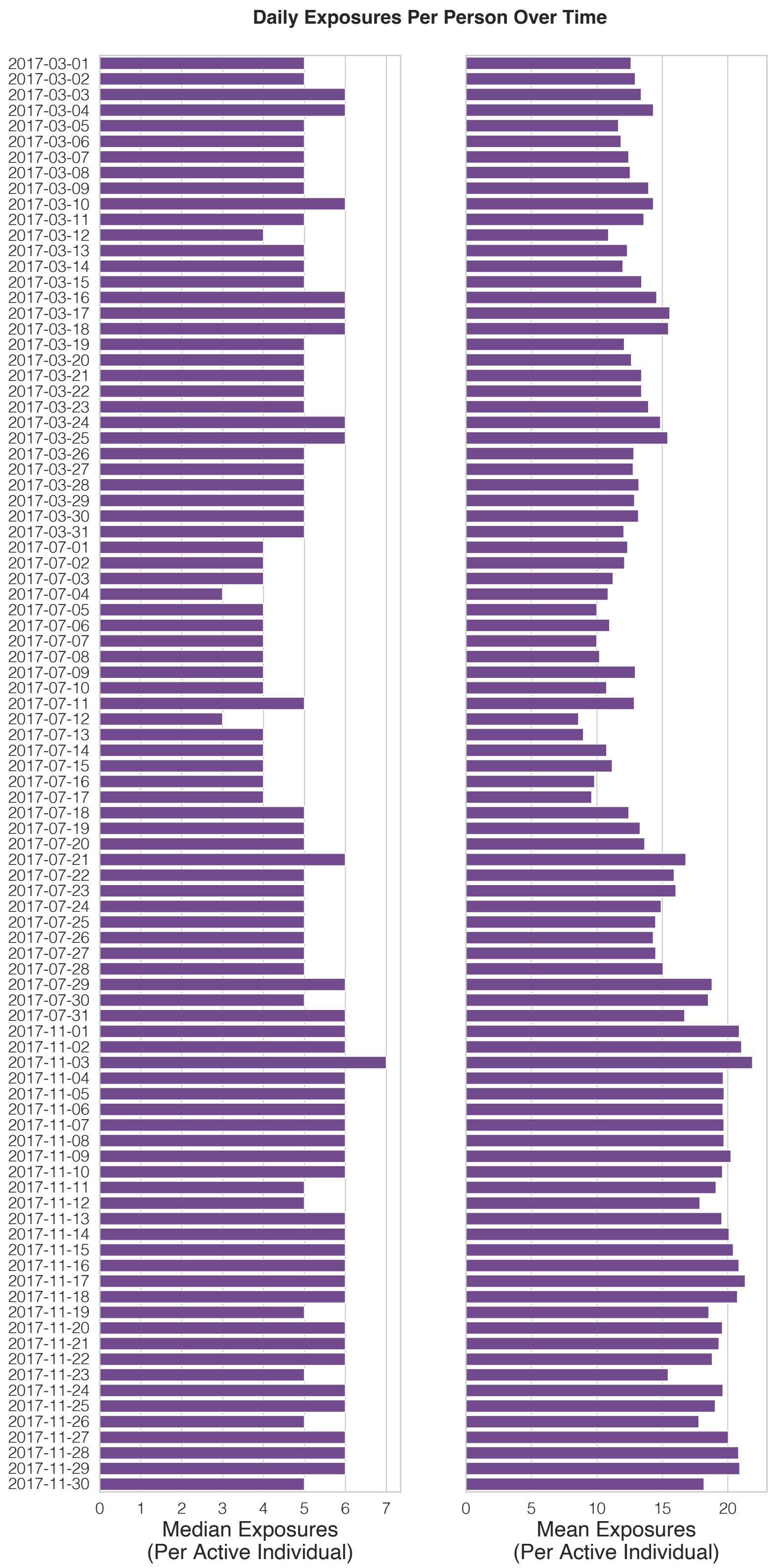

Supplementary Figure S28: Average number of exposures over time. Mean/median exposures per active individuals (i.e. nodes in the network) over the study observation period. Activity is defined as one or more exposures occurring on a given day.

Percentile	Tie Strength	Percentile	Tie Strength
0.0	1	51.0	1
1.0	1	52.0	1
2.0	1	53.0	1
3.0	1	54.0	1
4.0	1	55.0	1
5.0	1	56.0	1
6.0	1	57.0	1
7.0	1	58.0	1
8.0	1	59.0	1
9.0	1	60.0	1
10.0	1	61.0	1
11.0	1	62.0	1
12.0	1	63.0	1
13.0	1	64.0	1
14.0	1	65.0	1
15.0	1	66.0	1
16.0	1	67.0	1
17.0	1	68.0	1
18.0	1	69.0	1
19.0	1	70.0	1
20.0	1	71.0	1
21.0	1	72.0	1
22.0	1	73.0	1
23.0	1	74.0	1
24.0	1	75.0	2
25.0	1	76.0	2
26.0	1	77.0	2
27.0	1	78.0	2
28.0	1	79.0	2
29.0	1	80.0	2
30.0	1	81.0	2
31.0	1	82.0	2
32.0	1	83.0	2
33.0	1	84.0	2
34.0	1	85.0	2
35.0	1	86.0	2
36.0	1	87.0	2
37.0	1	88.0	2
38.0	1	89.0	2
39.0	1	90.0	2
40.0	1	91.0	3
41.0	1	92.0	3
42.0	1	93.0	3
43.0	1	94.0	3
44.0	1	95.0	4
45.0	1	96.0	4
46.0	1	97.0	5
47.0	1	98.0	7
48.0	1	99.0	11
49.0	1	100.0	11644
50.0	1		

Supplementary Table S10: Distribution of tie strength (# of exposures) for all pairs of individuals residing in 382 Metropolitan Statistical Areas (MSAs).

Percentile	Accurate Pings	Raw Pings	Percentile	Accurate Pings	Raw Pings
0	11	500	51	1,507	1,668
1	370	513	52	1,544	1,706
2	424	526	53	1,582	1,745
3	458	541	54	1,621	1,786
4	483	555	55	1,661	1,827
5	501	571	56	1,702	1,869
6	514	586	57	1,745	1,913
7	528	602	58	1,789	1,958
8	542	618	59	1,834	2,004
9	556	634	60	1,880	2,052
10	570	651	61	1,927	2,101
11	585	668	62	1,976	2,152
12	600	686	63	2,027	2,204
13	616	703	64	2,080	2,259
14	631	721	65	2,134	2,315
15	647	739	66	2,190	2,374
16	664	757	67	2,249	2,434
17	680	776	68	2,310	2,498
18	697	795	69	2,373	2,565
19	714	814	70	2,440	2,634
20	731	833	71	2,509	2,708
21	749	852	72	2,582	2,785
22	767	872	73	2,659	2,867
23	785	892	74	2,740	2,955
24	803	913	75	2,827	3,048
25	822	933	76	2,919	3,148
26	842	955	77	3,019	3,256
27	861	976	78	3,125	3,372
28	881	998	79	3,241	3,498
29	901	1,021	80	3,367	3,636
30	922	1,043	81	3,504	3,788
31	944	1,067	82	3,656	3,954
32	966	1,090	83	3,824	4,139
33	988	1,114	84	4,011	4,344
34	1,011	1,139	85	4,220	4,576
35	1,034	1,164	86	4,460	4,837
36	1,058	1,190	87	4,733	5,137
37	1,083	1,216	88	5,051	5,479
38	1,108	1,243	89	5,420	5,873
39	1,134	1,271	90	5,857	6,327
40	1,160	1,300	91	6,370	6,862
41	1,187	1,329	92	6,987	7,492
42	1,215	1,360	93	7,735	8,258
43	1,244	1,391	94	8,669	9,211
44	1,274	1,423	95	9,885	10,444
45	1,304	1,456	96	11,543	12,116
46	1,336	1,489	97	14,011	14,602
47	1,369	1,523	98	18,150	18,735
48	1,402	1,558	99	27,407	27,938
49	1,436	1,594	100	4,755,081	4,777,213
50	1,471	1,630			

Supplementary Table S11: Distribution of total pings for all included individuals residing in 382 Metropolitan Statistical Areas (MSAs). The median individual has 1,471 accurate pings. Accurate pings are those with $<$ 100 meters error.

Percentile	Unique Days	Percentile	Unique Days
0	2	51	30
1	5	52	31
2	7	53	31
3	8	54	31
4	9	55	31
5	9	56	31
6	10	57	31
7	11	58	32
8	11	59	32
9	12	60	32
10	13	61	32
11	13	62	32
12	14	63	33
13	15	64	34
14	15	65	34
15	16	66	35
16	16	67	37
17	17	68	38
18	17	69	39
19	18	70	40
20	18	71	41
21	19	72	43
22	19	73	44
23	20	74	45
24	20	75	47
25	21	76	48
26	21	77	49
27	22	78	51
28	22	79	52
29	23	80	53
30	23	81	54
31	24	82	56
32	24	83	57
33	25	84	58
34	25	85	59
35	25	86	60
36	26	87	61
37	26	88	62
38	26	89	62
39	27	90	63
40	27	91	64
41	27	92	67
42	27	93	70
43	28	94	74
44	28	95	78
45	28	96	82
46	29	97	86
47	29	98	90
48	29	99	93
49	30	100	95
50	30		

Supplementary Table S12: Distribution of unique days of ping data coverage for all included individuals residing in 382 Metropolitan Statistical Areas (MSAs). The median individual has 30 days of data coverage.

Percentile	Time Elapsed Between Pings (seconds)	Percentile	Time Elapsed Between Pings (seconds)
0	1	51	6
1	1	52	7
2	1	53	8
3	1	54	9
4	1	55	9
5	1	56	11
6	1	57	14
7	1	58	18
8	1	59	24
9	1	60	31
10	1	61	40
11	1	62	50
12	1	63	60
13	1	64	65
14	1	65	73
15	1	66	83
16	1	67	96
17	2	68	112
18	2	69	124
19	2	70	138
20	2	71	154
21	2	72	172
22	2	73	182
23	3	74	190
24	3	75	203
25	3	76	221
26	3	77	241
27	4	78	261
28	4	79	288
29	4	80	300
30	4	81	304
31	5	82	322
32	5	83	357
33	5	84	402
34	5	85	473
35	5	86	561
36	5	87	603
37	5	88	640
38	5	89	742
39	5	90	897
40	5	91	1,081
41	5	92	1,355
42	5	93	1,679
43	5	94	1,888
44	5	95	2,418
45	5	96	3,482
46	5	97	4,732
47	5	98	8,185
48	5	99	21,454
49	6	100	23,053,986
50	6		

Supplementary Table S13: Distribution of interevent times (seconds elapsed between pings) for all included individuals residing in 382 Metropolitan Statistical Areas (MSAs). The median interevent time is 6 seconds, and 96% of intervent times are $<$ 60 minutes. Distribution is estimated using a random sample of 1% of users, corresponding to 326,039,078 pings

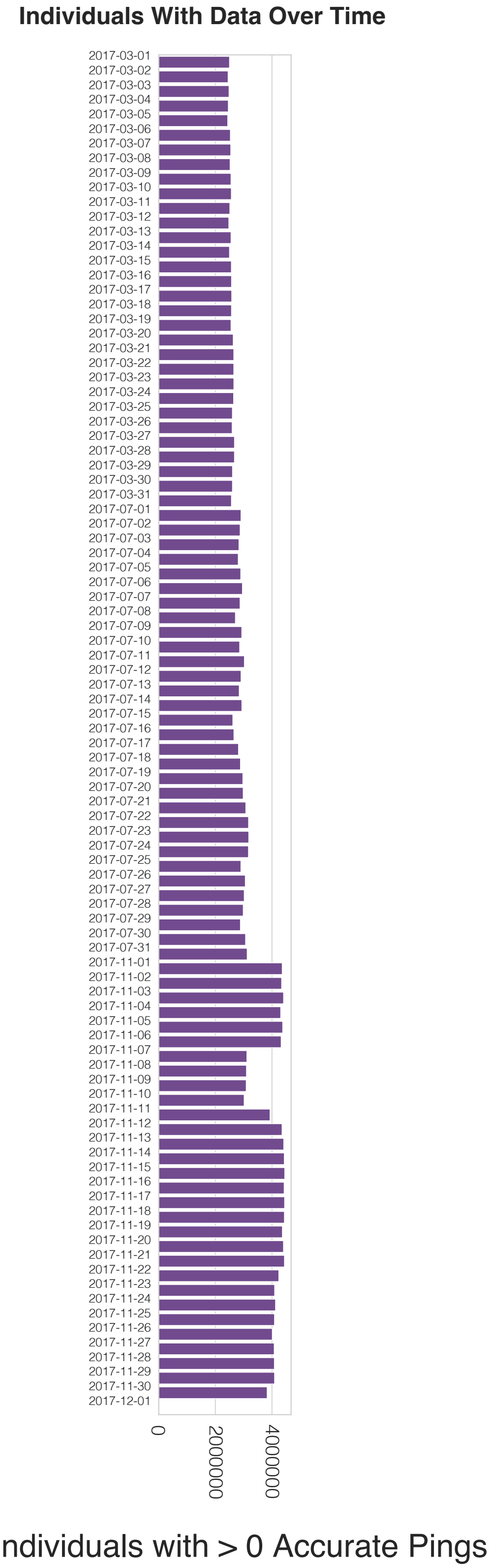

Supplementary Figure S29: Individuals with data coverage over time. Number of individuals with data (i.e. > 0 accurate pings) over the study observation period. Accurate pings are those with < 100 meters error.

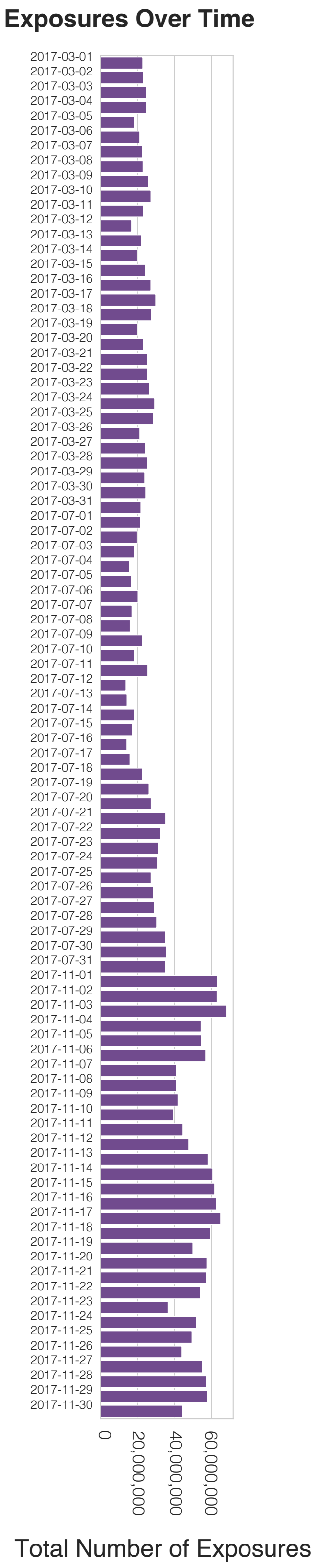

Supplementary Figure S30: Exposures over time across all individuals residing in the 382 MSAs.

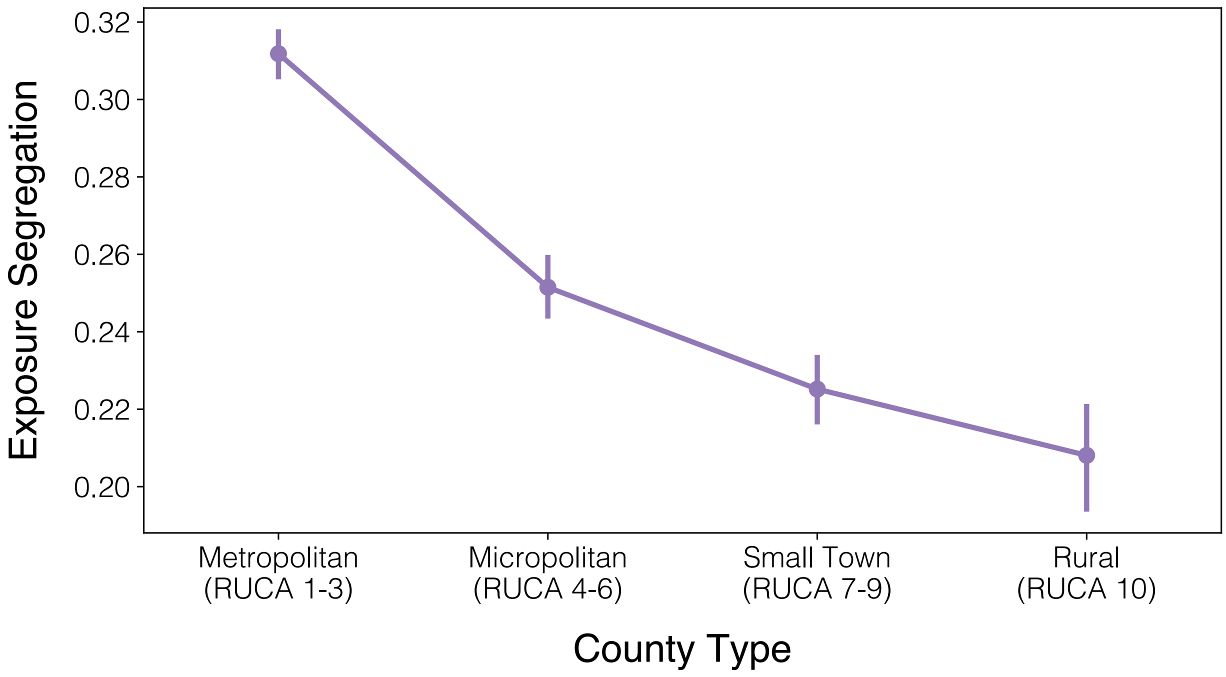

Supplementary Figure S31: Counties grouped by extent of county urbanization. Urbanized counties are more segregated than rural counties. To operationalize county urbanization, we use the median rural urban continuum (RUCA code) of the tracts in a county.[13]. Y axis corresponds to mean values across counties of a category; error bars correspond to 95% bootstrap confidence intervals (Methods 'Hypothesis testing'). N = 1093, 710, 651, 375 across metropolitan, micropolitan, small town, and rural counties respectively.

Mean # of Unique Days of Exposure	POI Type
1.027096	Performing Arts Centers
1.029548	Stadiums
1.036697	Theme Parks
1.044692	Bowling Centers
1.050582	Other Amusement/Recreation
1.054688	Bars/Drinking Places
1.068597	Museums
1.068676	Historical Sites
1.073173	Independent Artists
1.087597	Casinos
1.089572	Limited-Service Restaurants
1.092399	Parks
1.097890	Snack Bars
1.102125	Full-Service Restaurants
1.117464	Fitness/Recreation Centers
1.147761	Golf Courses and Country Clubs
1.153269	Religious Organizations

Supplementary Table S14: Exposure repetition by setting. For each leisure POI category, we compute the mean number of unique days of exposure over all pairs of individuals in the exposure network. POIs associated with most repeated exposures (religious organizations, golf courses and country clubs, and fitness centers) are all venues with relatively static membership structures (e.g. religious affiliation, annual gym membership) and in which frequent attendance is a norm (e.g. visiting church every Sunday, weekly workout). By contrast, the POIs with least repetition (performing arts centers and stadiums) are those which are typically attended only special occasions and typically without a commitments (e.g. buying a single ticket to see a sports game).

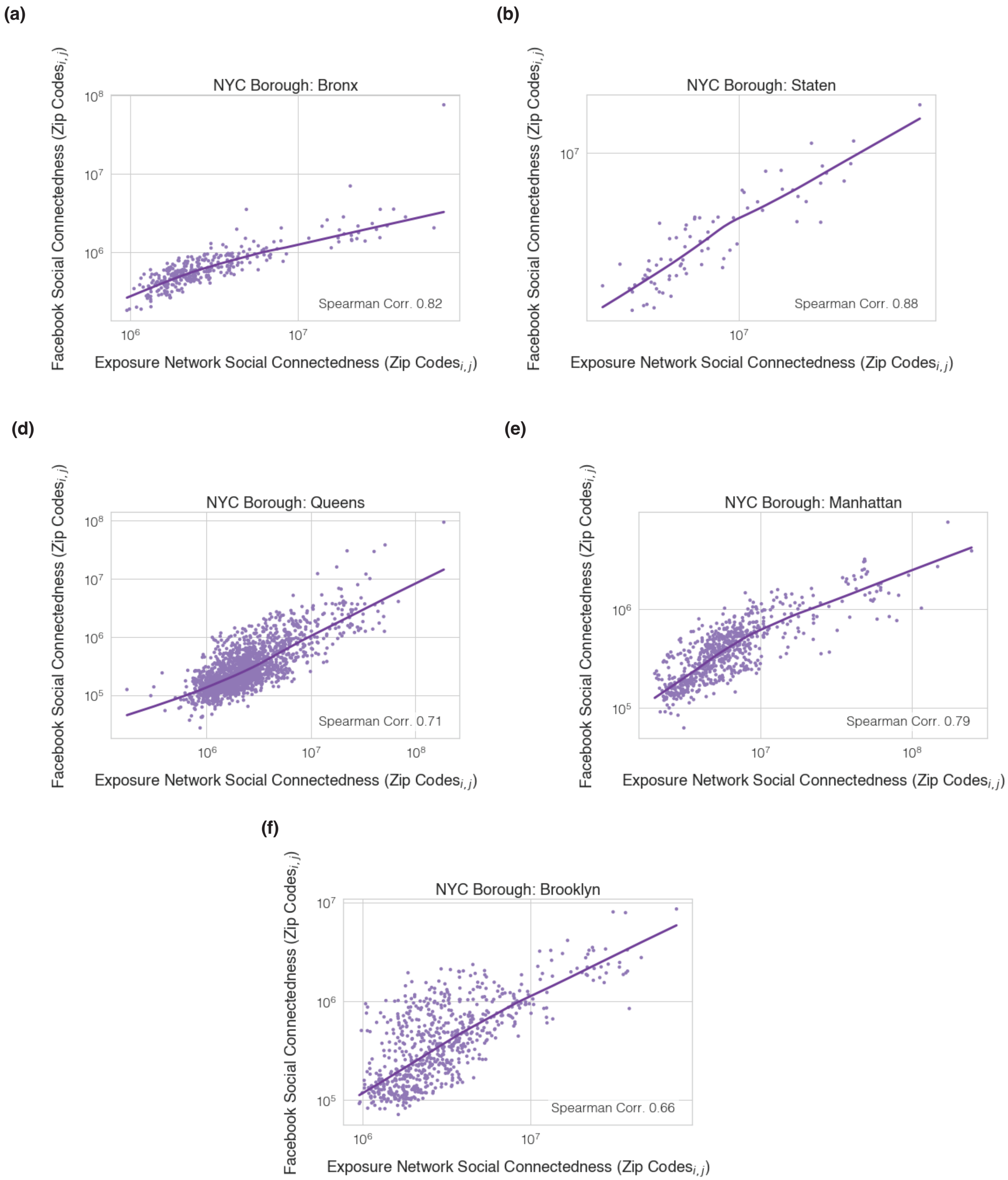

Supplementary Figure S32: Exposure network connections strongly correlate to friendship formation even within fine-grained geographical areas. We reproduce the the Facebook Social Connectedness Index[14] at zip code-level (Extended Data Figure 2) for each of the five boroughs of New York City, and find strong correlations to online friendships in all five boroughs (Spearman Correlations 0.66-0.88, all $p < 10^{-4}$; Two-sided Student's t-tests; Methods 'Hypothesis testing'). Strong correlations suggest that exposure segregation is likely related to segregation of friendships and other strong social ties.

Mean Exposure Distance (meters)	POI Type
14.148335	Museums
20.783428	Historical Sites
23.343675	Golf Courses and Country Clubs
24.539538	Religious Organizations
24.954691	Stadiums
25.063481	Performing Arts Centers
25.168213	Bowling Centers
25.765411	Full-Service Restaurants
25.845487	Parks
25.973838	Other Amusement/Recreation
26.080283	Bars/Drinking Places
26.102137	Fitness/Recreation Centers
26.214838	Theme Parks
26.305469	Limited-Service Restaurants
26.423661	Snack Bars
26.775229	Independent Artists
27.048224	Casinos

Supplementary Table S15: Exposure distance by setting. For each leisure POI category, we compute the mean distance during exposure over all pairs of individuals in the exposure network. POIs associated with furthest exposures (casinos, independent artists) are those in which mobile phone usage is typically restricted (e.g. many casinos do not allow mobile phone usage to ensure fair play), which is likely to lead to sparse GPS pings. By contrast, the POIs with least distance (museums, historical sites) are those which phones may be actively used to enhance the experience (e.g. to take photos, look up information, or use a virtually guided tour).

Mean Length (# of consecutive five minute intervals)	POI Type
1.617740	Museums
1.620565	Theme Parks
1.693283	Other Amusement/Recreation
1.748765	Bars/Drinking Places
1.776146	Independent Artists
1.782905	Limited-Service Restaurants
1.840704	Full-Service Restaurants
1.851464	Snack Bars
1.871852	Casinos
1.882052	Fitness/Recreation Centers
1.910205	Historical Sites
2.039442	Parks
2.061254	Religious Organizations
2.082550	Golf Courses and Country Clubs
2.209579	Bowling Centers
2.436712	Stadiums
2.464210	Performing Arts Centers

Supplementary Table S16: Exposure length by setting. For each leisure POI category, we compute the mean length during exposure over all pairs of individuals in the exposure network (in number of consecutive 5 minute intervals). POIs associated with longest exposures (performing arts centers, stadiums) are those in which attendance is typically prolonged and mobility is restricted (e.g. sitting in the same seat for multiple hours to watch a game). By contrast, the POIs with shortest exposure (museums, theme parks) are those which mobility is a necessary part of the experience (e.g. walking to different exhibits or attractions).

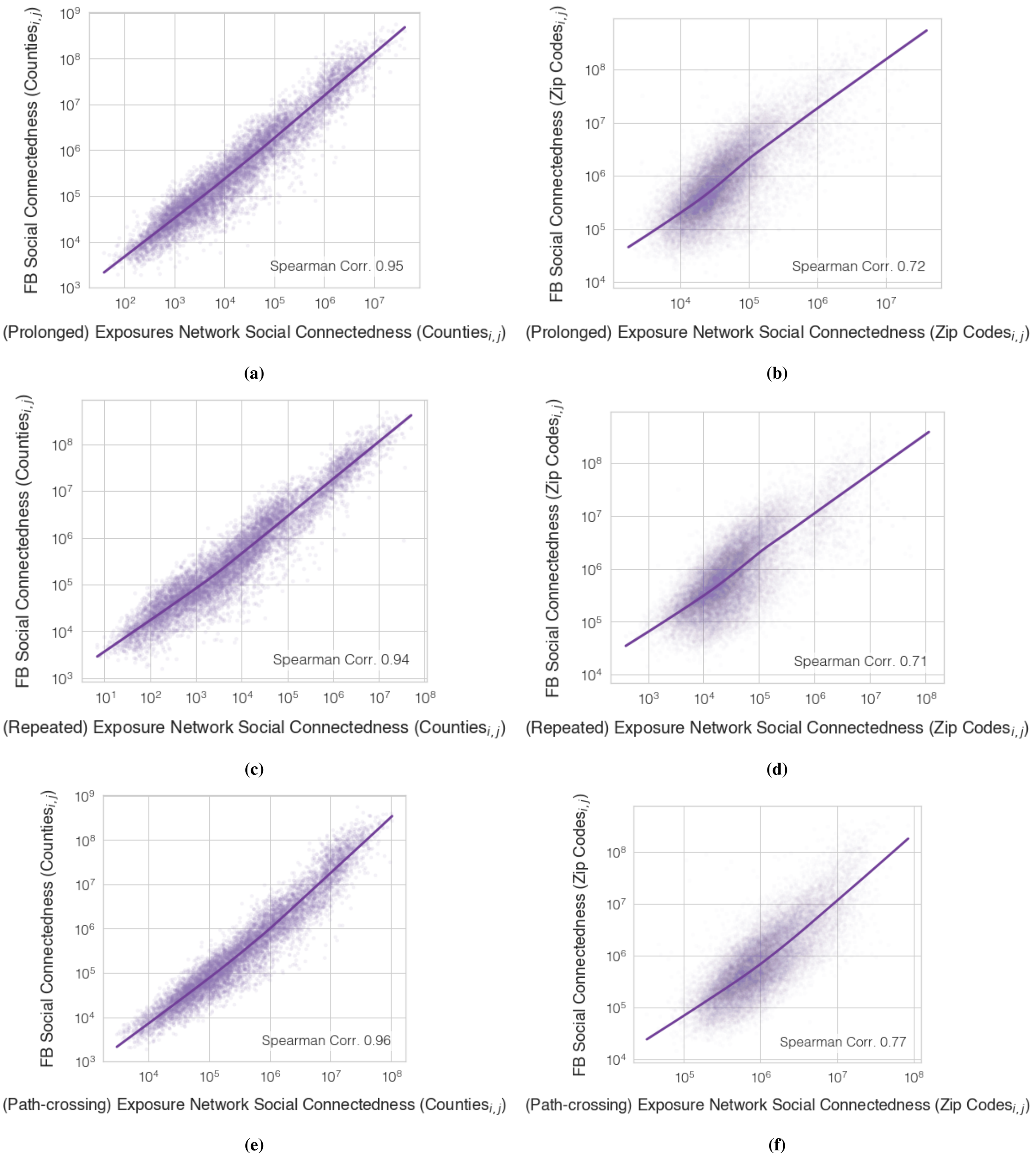

Supplementary Figure S33: We measure the external validity of alternative measures in which the strictness of our exposure definition is varied. We filter the exposure network to include (a-b) only prolonged exposure of 3+ consecutive intervals of exposure on the same day, (c-d) repeated exposure of 3+ consecutive intervals of exposure on different days, and (e-f) path crossings (i.e. pairs of users that had only one instance of being within proximity of each other). We find that social connectedness across all three definitions of exposure correlates strongly to social connectedness measured by online friendship linked (detailed in Extended Data Figure 2)

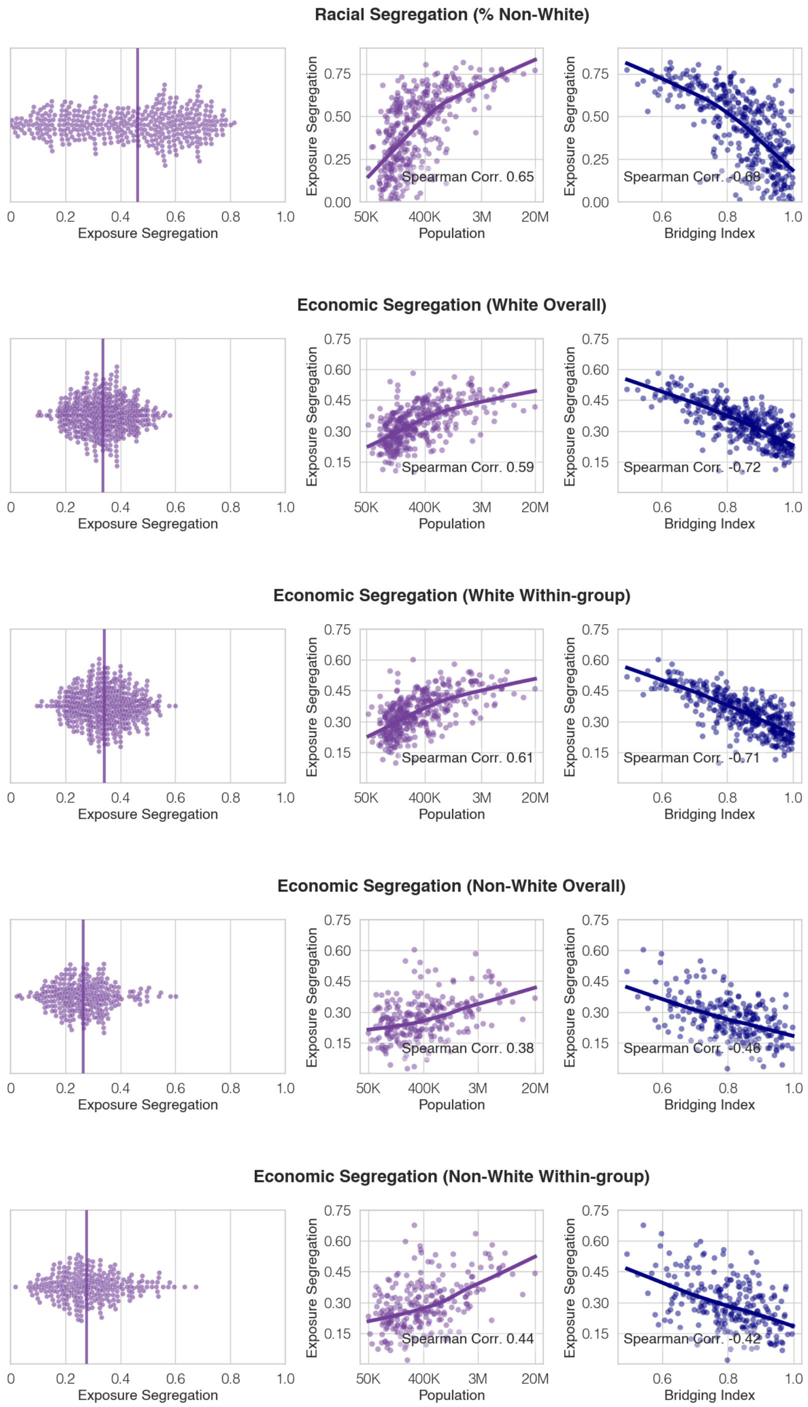

Supplementary Figure S34: Racial exposure segregation, and economic exposure segregation broken down by race group. We find that our primary study findings that, (1) large, dense cities are more segregated and (2) hub locations accessible to diverse individuals may mitigate segregation, are robust to replacing socioeconomic status with race (using % non-White in home neighborhoods), and to isolated exposures of individuals residing in predominantly White and non-White neighborhoods. Neighborhood is operationalized as census block group. Overall, racial exposure segregation (top row) is 31% higher in the median MSA compared to the economic exposure segregation in the median MSA. Moreover, there is much higher variance in racial exposure segregation: the standard deviation of racial exposure segregation is 114% higher than the standard deviation of racial exposure segregation. Additionally, economic exposure segregation is 27% higher for White individuals. However, for both groups, economic segregation is similar when comparing within-race exposures to overall exposures.

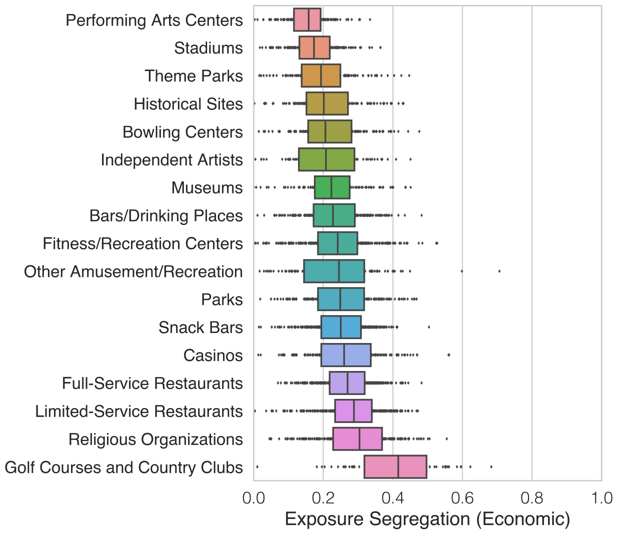

(a)

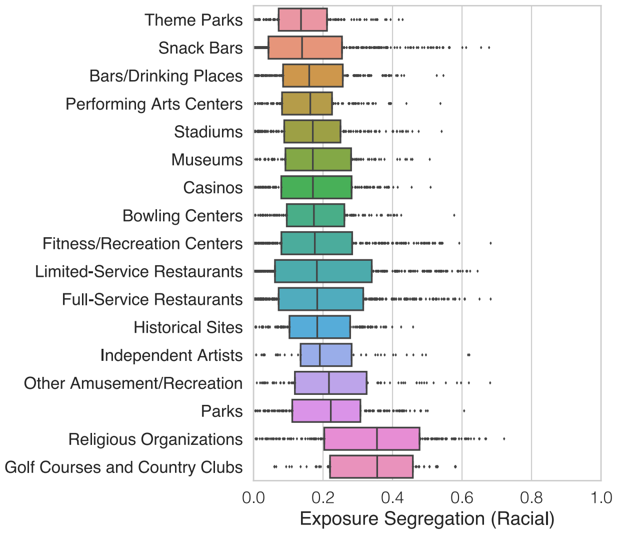

(b)

Supplementary Figure S35: Comparing economic and racial exposure segregation by POI. Each point represents segregation in one of N=382 MSAs using only exposure pairs occurring in a given location type. Centre indicates median across MSAs. Boxes indicate the interquartile range. Whiskers extend from the box to the furthest data point within 1.5 times the IQR. Dots indicate outliers that extend beyond 1.5 times the IQR. We observe significant differences between how POIs are segregated by race vs. economic status. For instance, full-service restaurants are 47% more segregated by economic status than by race. At the same time, golf courses and country clubs, as well as religious organizations, are the most segregated across both socioeconomic status and race. The least racially segregated POI category is theme parks, whereas the least economically segregated POI category is performing arts centers.

Percentile	# Distinct Exposures	Percentile	# Distinct Exposures
0.0	1.0	51.0	79.0
1.0	1.0	52.0	81.0
2.0	2.0	53.0	85.0
3.0	3.0	54.0	88.0
4.0	3.0	55.0	91.0
5.0	4.0	56.0	94.0
6.0	5.0	57.0	98.0
7.0	6.0	58.0	102.0
8.0	7.0	59.0	106.0
9.0	8.0	60.0	109.0
10.0	8.0	61.0	114.0
11.0	9.0	62.0	118.0
12.0	10.0	63.0	122.0
13.0	11.0	64.0	127.0
14.0	12.0	65.0	132.0
15.0	13.0	66.0	137.0
16.0	14.0	67.0	142.0
17.0	15.0	68.0	148.0
18.0	17.0	69.0	154.0
19.0	18.0	70.0	160.0
20.0	19.0	71.0	167.0
21.0	20.0	72.0	173.0
22.0	21.0	73.0	181.0
23.0	23.0	74.0	188.0
24.0	24.0	75.0	196.0
25.0	25.0	76.0	205.0
26.0	27.0	77.0	214.0
27.0	28.0	78.0	224.0
28.0	30.0	79.0	234.0
29.0	31.0	80.0	245.0
30.0	33.0	81.0	257.0
31.0	34.0	82.0	271.0
32.0	36.0	83.0	285.0
33.0	38.0	84.0	300.0
34.0	40.0	85.0	317.0
35.0	41.0	86.0	336.0
36.0	43.0	87.0	357.0
37.0	45.0	88.0	380.0
38.0	47.0	89.0	406.0
39.0	49.0	90.0	436.0
40.0	51.0	91.0	471.0
41.0	53.0	92.0	511.0
42.0	55.0	93.0	560.0
43.0	58.0	94.0	620.0
44.0	60.0	95.0	695.0
45.0	62.0	96.0	796.0
46.0	65.0	97.0	939.0
47.0	67.0	98.0	1171.0
48.0	70.0	99.0	1655.0
49.0	73.0	100.0	42323.0
50.0	76.0		

Supplementary Table S17: Distribution of number of distinct exposures for all individuals residing in 382 Metropolitan Statistical Areas (MSAs). The median individual had exposures to 76 distinct people. 8,609,406 individuals reside in a Metropolitan Statistical Area (90% of the overall 9,567,559 individuals in our study). The remaining 958,153 users live outside of MSAs, influencing the exposure segregation of an MSA by coming into contact with MSA residents.

Percentile	# of Distinct Exposures	Percentile	# of Distinct Exposures
0.0	1.00	51.0	4.55
1.0	1.00	52.0	4.65
2.0	1.00	53.0	4.76
3.0	1.00	54.0	4.87
4.0	1.14	55.0	5.00
5.0	1.23	56.0	5.09
6.0	1.30	57.0	5.21
7.0	1.36	58.0	5.33
8.0	1.43	59.0	5.46
9.0	1.50	60.0	5.60
10.0	1.56	61.0	5.73
11.0	1.62	62.0	5.87
12.0	1.67	63.0	6.00
13.0	1.75	64.0	6.17
14.0	1.80	65.0	6.32
15.0	1.87	66.0	6.49
16.0	1.94	67.0	6.66
17.0	2.00	68.0	6.83
18.0	2.00	69.0	7.00
19.0	2.10	70.0	7.21
20.0	2.17	71.0	7.41
21.0	2.23	72.0	7.62
22.0	2.29	73.0	7.86
23.0	2.36	74.0	8.09
24.0	2.42	75.0	8.33
25.0	2.50	76.0	8.61
26.0	2.55	77.0	8.89
27.0	2.62	78.0	9.20
28.0	2.68	79.0	9.52
29.0	2.75	80.0	9.87
30.0	2.82	81.0	10.24
31.0	2.89	82.0	10.65
32.0	3.00	83.0	11.08
33.0	3.00	84.0	11.57
34.0	3.10	85.0	12.09
35.0	3.17	86.0	12.67
36.0	3.25	87.0	13.33
37.0	3.33	88.0	14.05
38.0	3.40	89.0	14.88
39.0	3.48	90.0	15.83
40.0	3.56	91.0	16.94
41.0	3.64	92.0	18.25
42.0	3.72	93.0	19.83
43.0	3.81	94.0	21.78
44.0	3.89	95.0	24.31
45.0	4.00	96.0	27.71
46.0	4.07	97.0	32.71
47.0	4.16	98.0	41.00
48.0	4.25	99.0	59.00
49.0	4.35	100.0	1740.25
50.0	4.45		

Supplementary Table S18: Distribution of average number of distinct exposures (per active day) for all individuals residing in 382 Metropolitan Statistical Areas (MSAs). The median individual had 4.45 unique exposures on the average day of activity. 8,609,406 individuals reside in a Metropolitan Statistical Area (90% of the overall 9,567,559 individuals in our study). The remaining 958,153 users live outside of MSAs, influencing the exposure segregation of an MSA by coming into contact with MSA residents. Activity is defined as one or more exposures occurring on a given day. For details on activity over time, see Supplementary Figure S27.

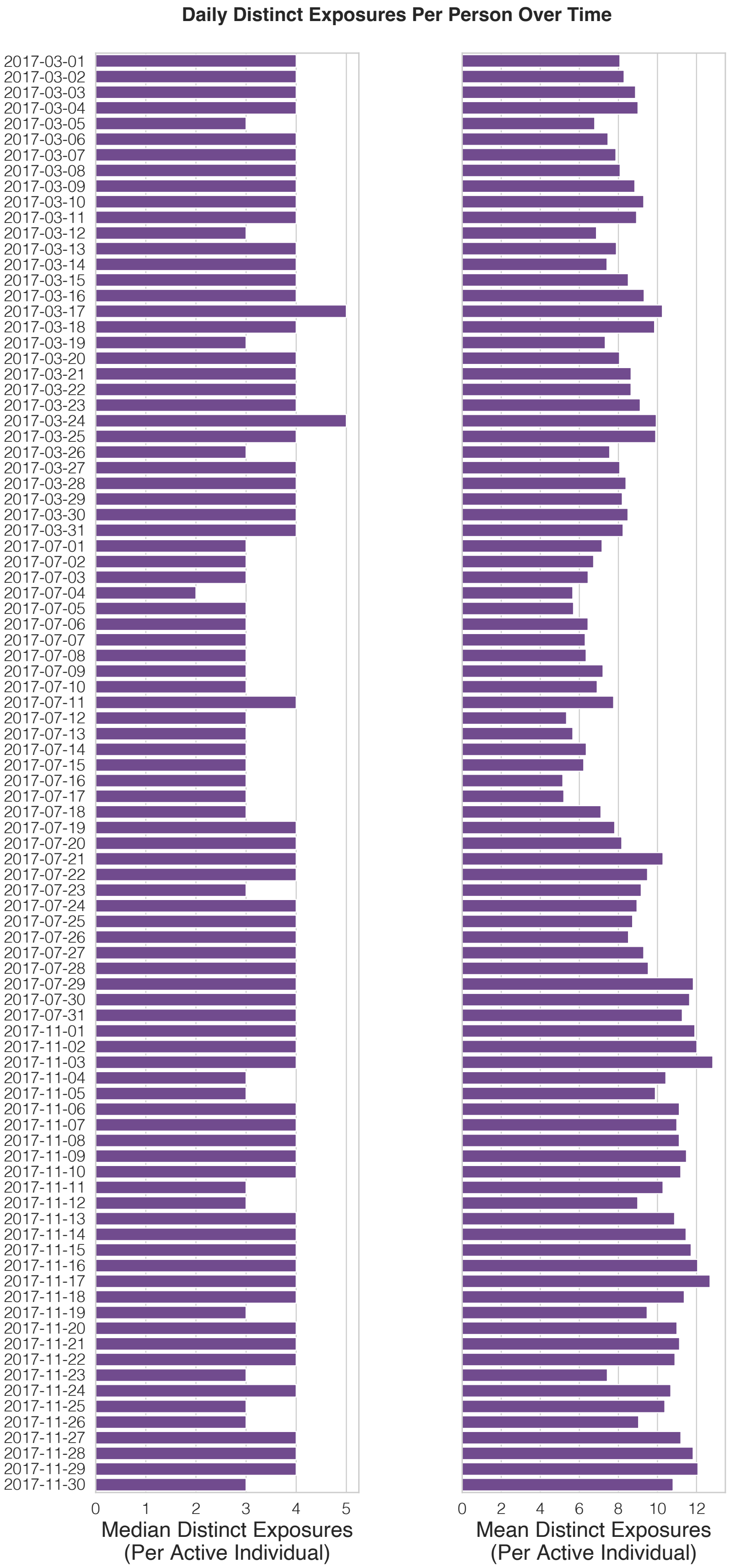

Supplementary Figure S36: Average number of distinct exposures over time. Mean/median distinct exposures per active individuals (i.e. nodes in the network) over the study observation period. Activity is defined as one or more exposures occurring on a given day.

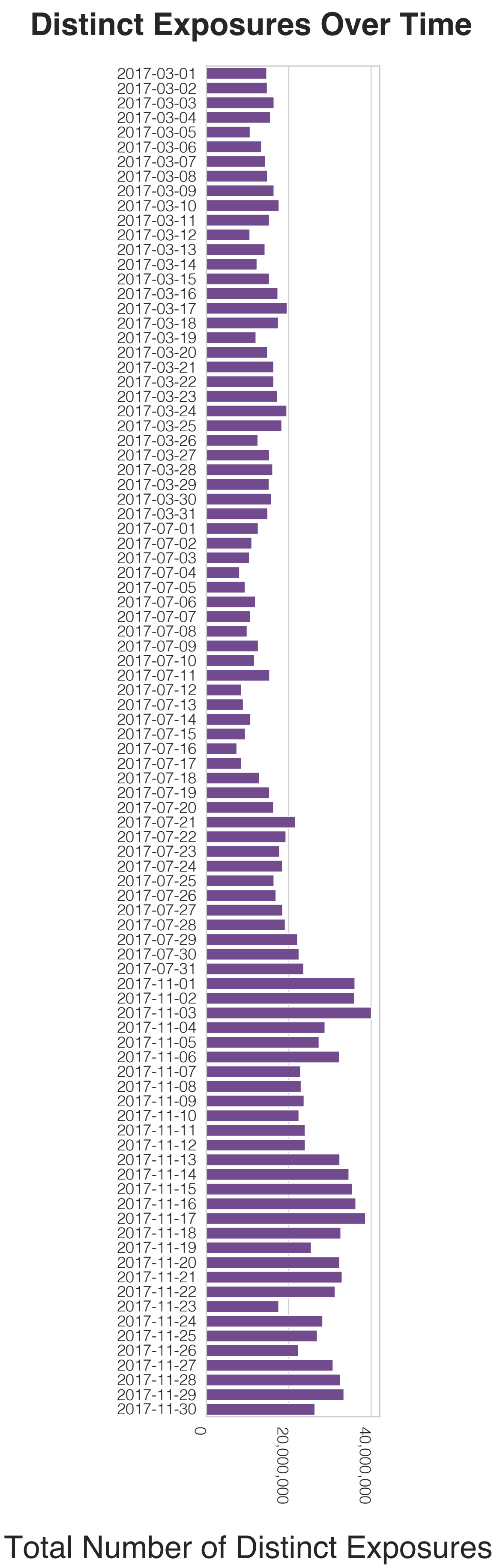

Supplementary Figure S37: Distinct exposures over time across all individuals residing in the 382 MSAs.

Measure	Mean	std	min	10%	25%	50%	75%	90%	max
Primary Measure	184.21	373.98	1.0	8.0	25.0	76.0	196.0	436.0	42323.0
Minimum Distance Between Pings: < 25m	93.23	200.13	1.0	4.0	12.0	37.0	97.0	219.0	26778.0
Minimum Distance Between Pings: < 10m	41.47	99.99	1.0	2.0	5.0	14.0	38.0	94.0	12907.0
Minimum Time Between Pings: < 2 minutes	99.92	212.03	1.0	5.0	14.0	41.0	104.0	232.0	26592.0
Minimum Time Between Pings: < 60 seconds	63.50	141.10	1.0	4.0	9.0	26.0	65.0	144.0	18912.0
Minimum Tie Strength: 2 consecutive exposures	35.51	91.98	1.0	2.0	5.0	13.0	33.0	78.0	13513.0
Minimum Tie Strength: 3 consecutive exposures	8.53	23.31	1.0	1.0	1.0	3.0	7.0	18.0	3107.0
Minimum Tie Strength: 2 unique days	16.39	54.78	1.0	1.0	2.0	5.0	13.0	33.0	15454.0
Minimum Tie Strength: 3 unique days	6.74	22.04	1.0	1.0	1.0	3.0	6.0	13.0	7685.0
Dist. < 25m, Time < 2 minutes, Length 2+ unique days	9.83	33.09	1.0	1.0	2.0	3.0	8.0	19.0	9951.0
Dist. < 25m, Time < 2 minutes, Length 2+ consecutive exposures	18.62	49.25	1.0	1.0	3.0	7.0	17.0	41.0	8447.0
Dist. < 10m, Time < 1 minutes, Length 3+ unique days	3.30	10.72	1.0	1.0	1.0	1.0	3.0	6.0	2019.0
Dist. < 10m, Time < 1 minutes, Length 3+ consecutive exposures	4.31	12.40	1.0	1.0	1.0	2.0	4.0	9.0	2893.0

Supplementary Table S19: Distribution of number of distinct exposures per person (by time, distance, and length threshold). We compute the number of distinct pairs of exposures for all residents of the 382 Metropolitan Statistical Areas (MSAs), for each robustness check which varies the time, distance, length thresholds for the definition of exposure. Summary of the per person number of distinct exposures.

Measure	Mean	std	min	10%	25%	50%	75%	90%	max
Primary Metric	7.90	13.90	1.0	1.56	2.50	4.45	8.33	15.83	1740.25
Minimum Distance Between Pings: < 25 meters	3.25	5.39	1.0	1.00	1.25	1.90	3.20	6.06	907.75
Minimum Distance Between Pings: < 10 meters	2.54	4.58	1.0	1.00	1.00	1.40	2.17	4.33	510.14
Minimum Time Between Pings: < 2 minutes	3.17	5.25	1.0	1.00	1.29	1.95	3.23	5.86	973.75
Minimum Time Between Pings: < 60 seconds	2.49	3.79	1.0	1.00	1.13	1.62	2.50	4.33	674.50
Minimum Tie Strength: 2 consecutive exposures	2.19	3.59	1.0	1.00	1.00	1.40	2.11	3.67	622.00
Minimum Tie Strength: 3 consecutive exposures	1.61	2.18	1.0	1.00	1.00	1.00	1.50	2.45	344.00
Dist. < 25 meters, Time < 2 minutes, Length 2+ consecutive exposures	1.76	2.30	1.0	1.00	1.00	1.18	1.75	2.89	1148.00
Dist. < 10 meters, Time < 60 minutes, Length 3+ consecutive exposures	1.43	2.03	1.0	1.00	1.00	1.00	1.30	2.00	342.00

Supplementary Table S20: Distribution of average number of distinct exposures (per active day) per person across all days of activity (by time, distance, and length threshold). Summary statistics are shown for for all residents of the 382 Metropolitan Statistical Areas (MSAs), for each robustness check which varies the time, distance, length thresholds for the definition of exposure. Activity is defined as one or more exposure occurring on a given day. Summary of the per person number of distinct exposures.

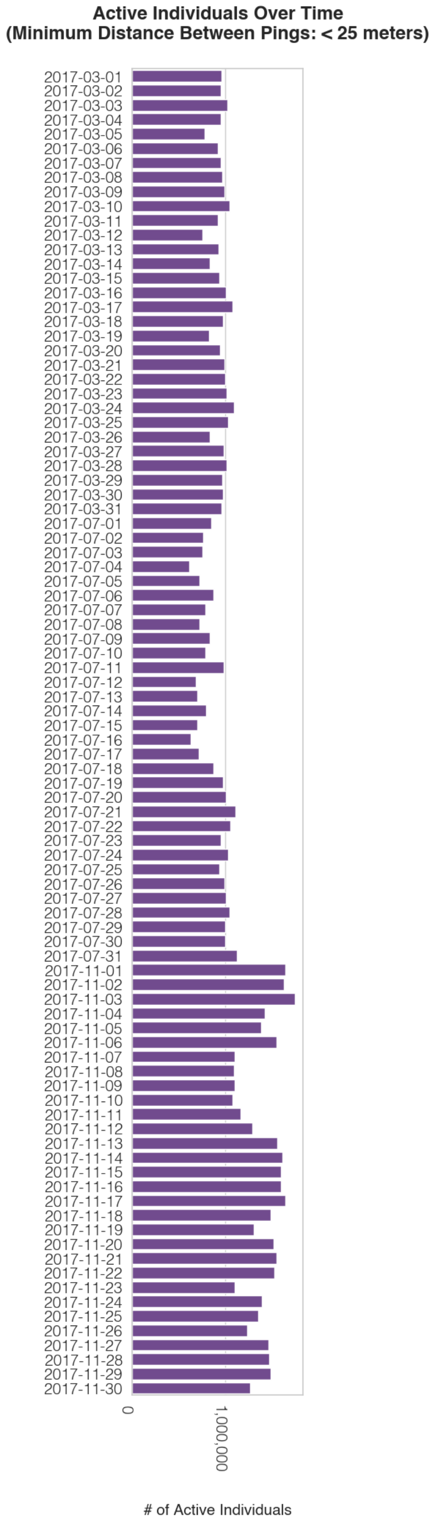

(a)

Supplementary Figure S38: Active individuals over time (exposure distance threshold: 25 meters). Number of active individuals (i.e. nodes in the network) over the study observation period. Activity is defined as one or more exposures occurring on a given day.

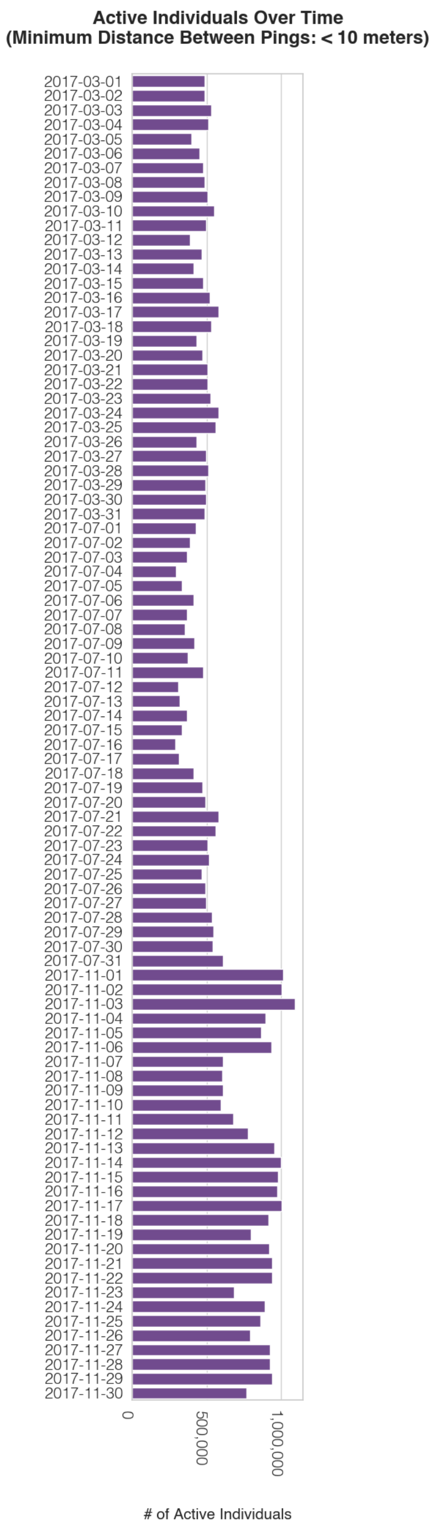

(a)

Supplementary Figure S39: Active individuals over time (exposure distance threshold: 10 meters). Number of active individuals (i.e. nodes in the network) over the study observation period. Activity is defined as one or more exposures occurring on a given day.

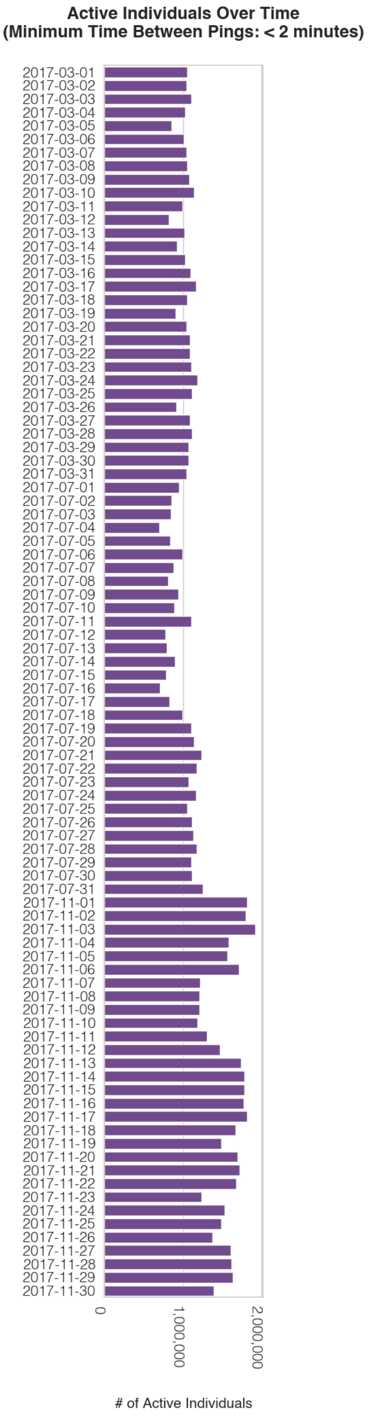

(a)

Supplementary Figure S40: Active individuals over time (exposure time threshold: 2 minutes). Number of active individuals (i.e. nodes in the network) over the study observation period. Activity is defined as one or more exposures occurring on a given day.

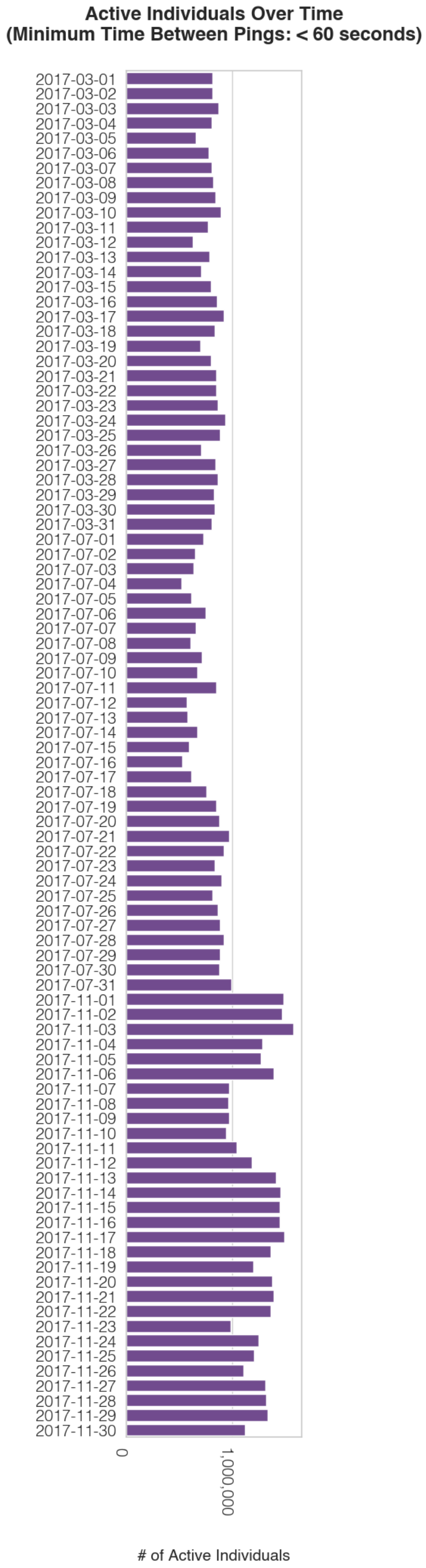

(a)

Supplementary Figure S41: Active individuals over time (exposure time threshold: 60 seconds). Number of active individuals (i.e. nodes in the network) over the study observation period. Activity is defined as one or more exposures occurring on a given day.

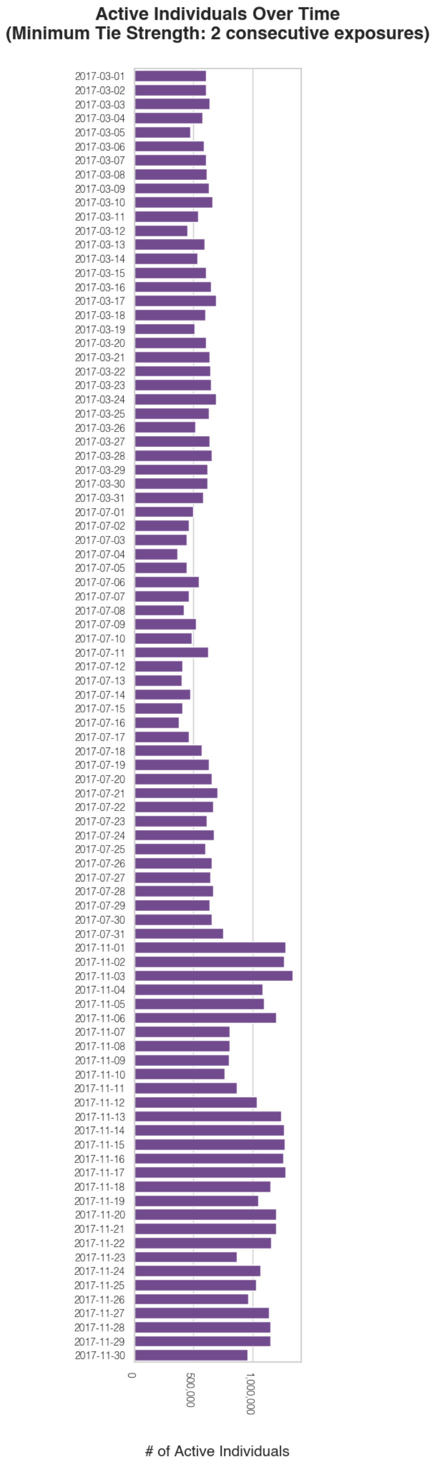

(a)

Supplementary Figure S42: Active individuals over time (exposure length threshold: 2 consecutive exposures over five minute intervals). Number of active individuals (i.e. nodes in the network) over the study observation period. Activity is defined as one or more exposures occurring on a given day.

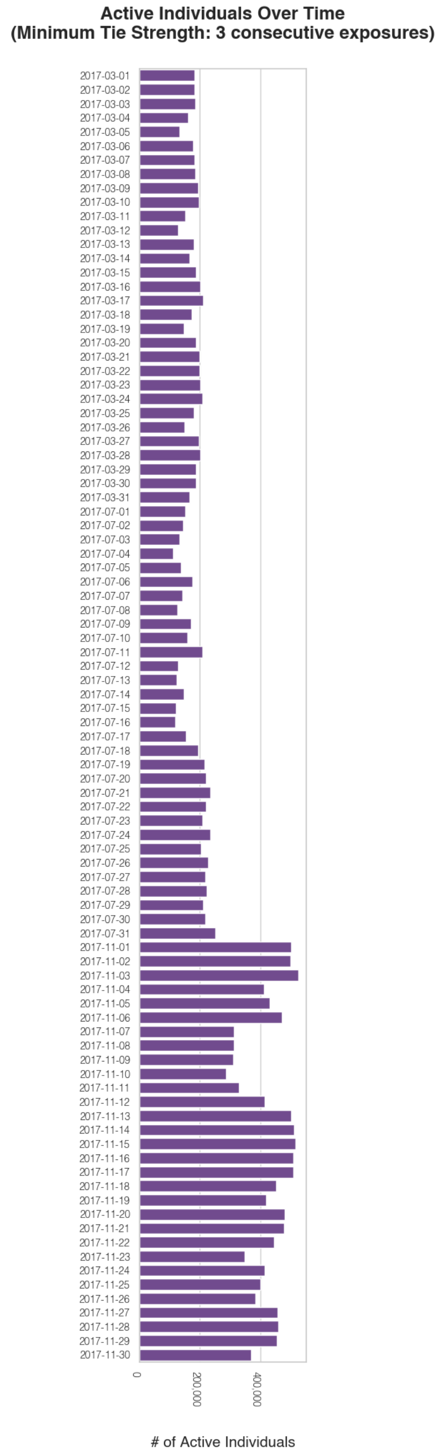

(a)

Supplementary Figure S43: Active individuals over time (exposure length threshold: 3 consecutive exposures over five minute intervals). Number of active individuals (i.e. nodes in the network) over the study observation period. Activity is defined as one or more exposures occurring on a given day.

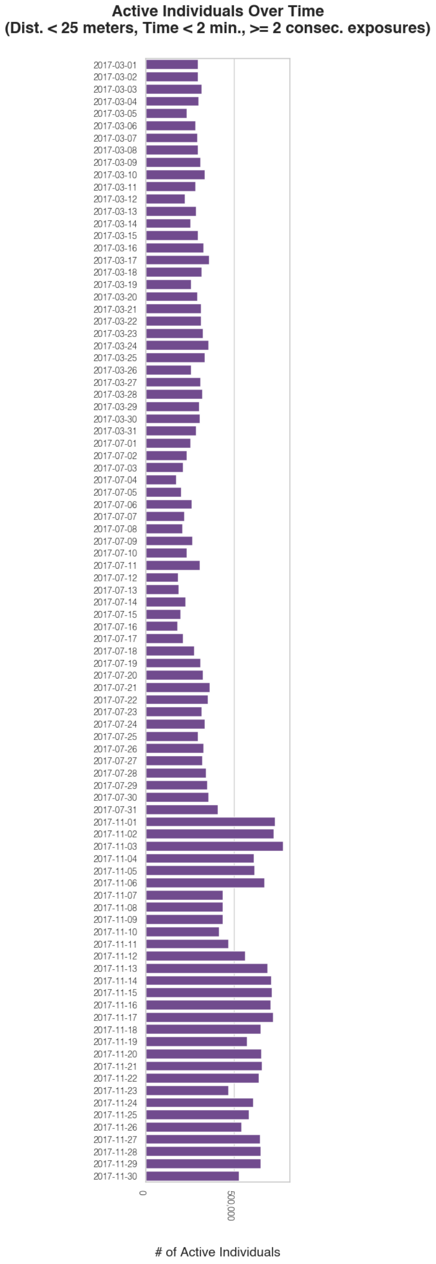

(a)

Supplementary Figure S44: Active individuals over time (distance threshold: 25 meters, time threshold: 2 minutes, length threshold: 2 consecutive exposures of five minute intervals). Number of active individuals (i.e. nodes in the network) over the study observation period. Activity is defined as one or more exposures occurring on a given day.

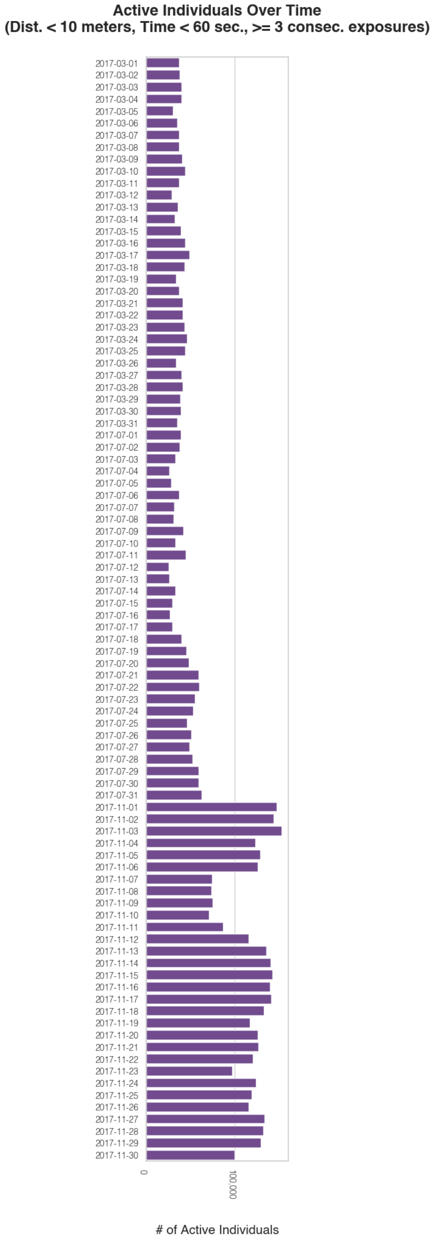

(a)

Supplementary Figure S45: Active individuals over time (distance threshold: 10 meters, time threshold: 60 seconds, length threshold: 3 consecutive exposures of five minute intervals). Number of active individuals (i.e. nodes in the network) over the study observation period. Activity is defined as one or more exposures occurring on a given day.

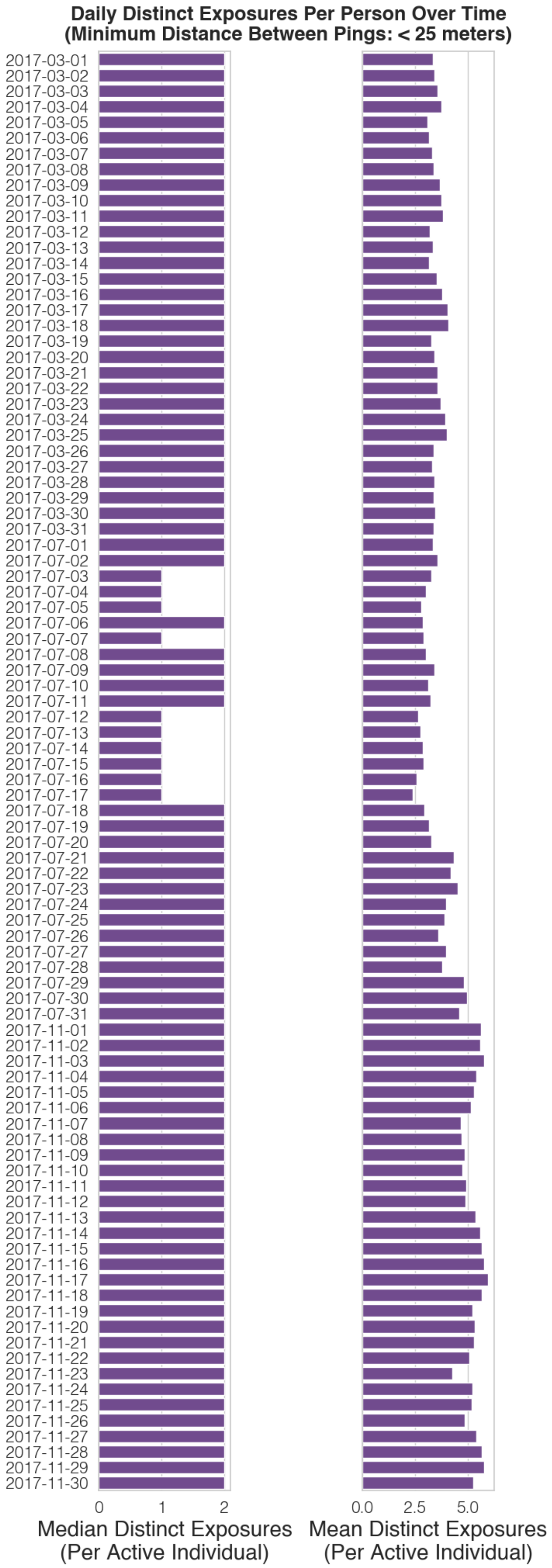

Supplementary Figure S46: Average number of distinct exposures over time (distance threshold: 25 meters). Mean/median distinct exposures per active individuals (i.e. nodes in the network) over the study observation period. Activity is defined as one or more exposures occurring on a given day.

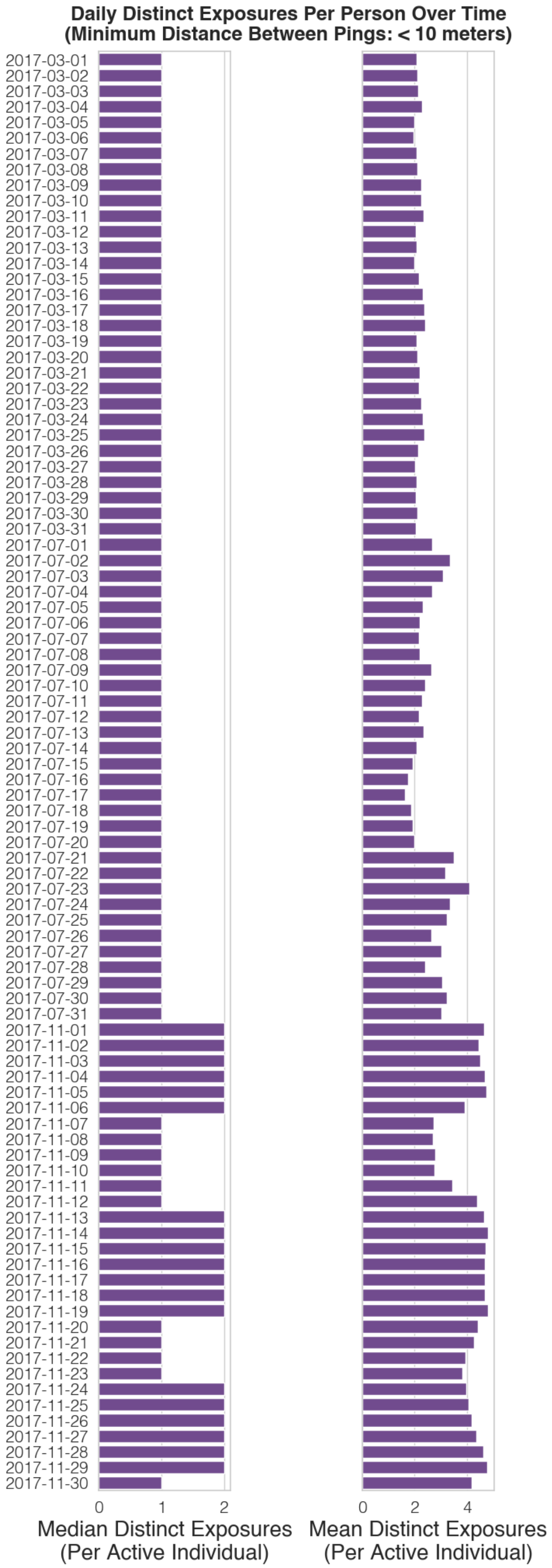

Supplementary Figure S47: Average number of distinct exposures over time (distance threshold: 10 meters). Mean/median distinct exposures per active individuals (i.e. nodes in the network) over the study observation period. Activity is defined as one or more exposures occurring on a given day.

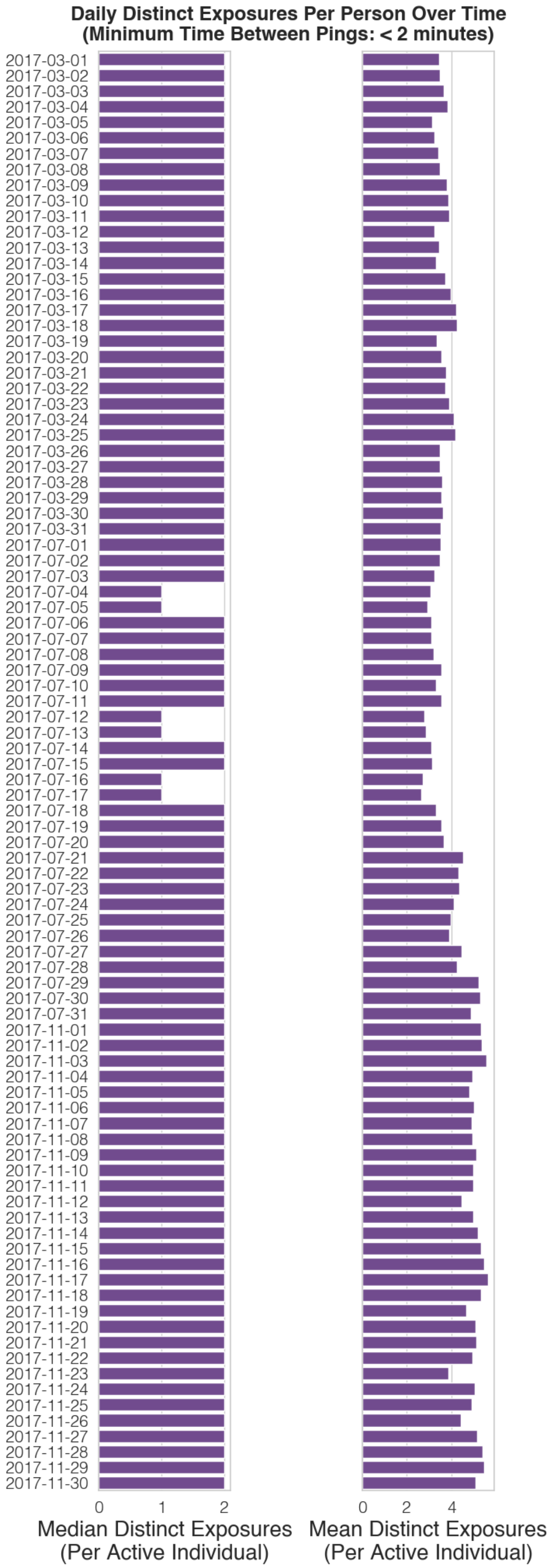

Supplementary Figure S48: Average number of distinct exposures over time (time threshold: 2 minutes). Mean/-median distinct exposures per active individuals (i.e. nodes in the network) over the study observation period. Activity is defined as one or more exposures occurring on a given day.

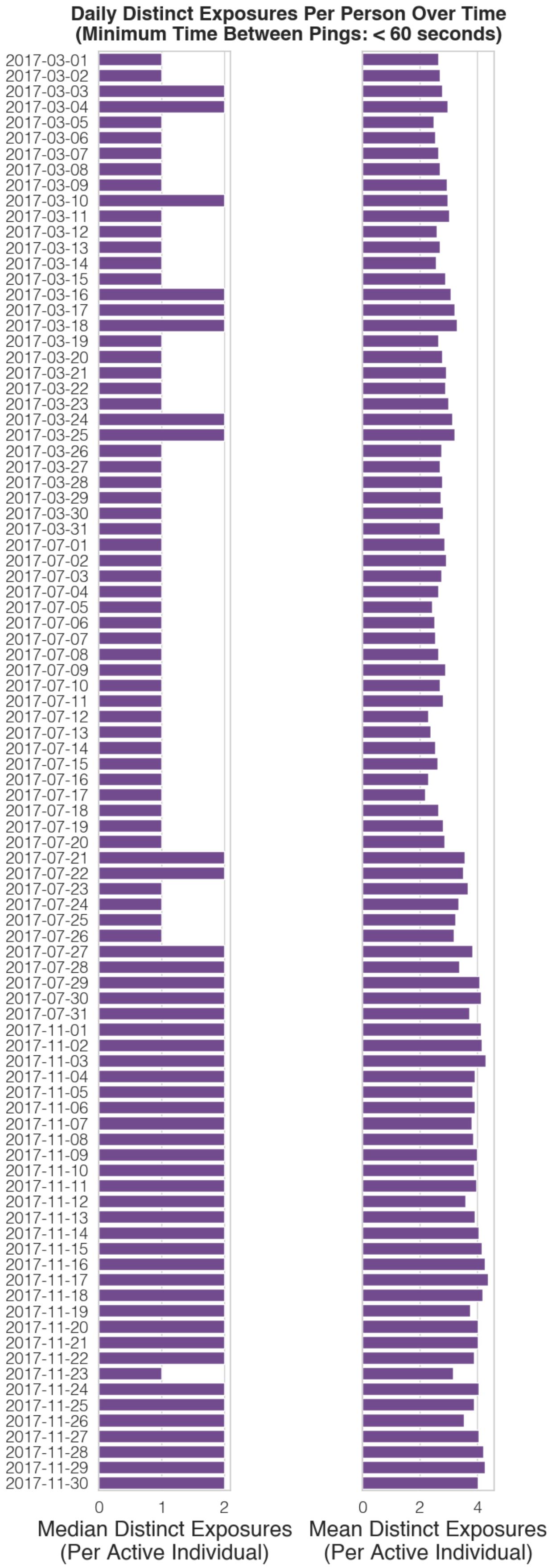

Supplementary Figure S49: Average number of distinct exposures over time (time threshold: 60 seconds). Mean/median distinct exposures per active individuals (i.e. nodes in the network) over the study observation period. Activity is defined as one or more exposures occurring on a given day.

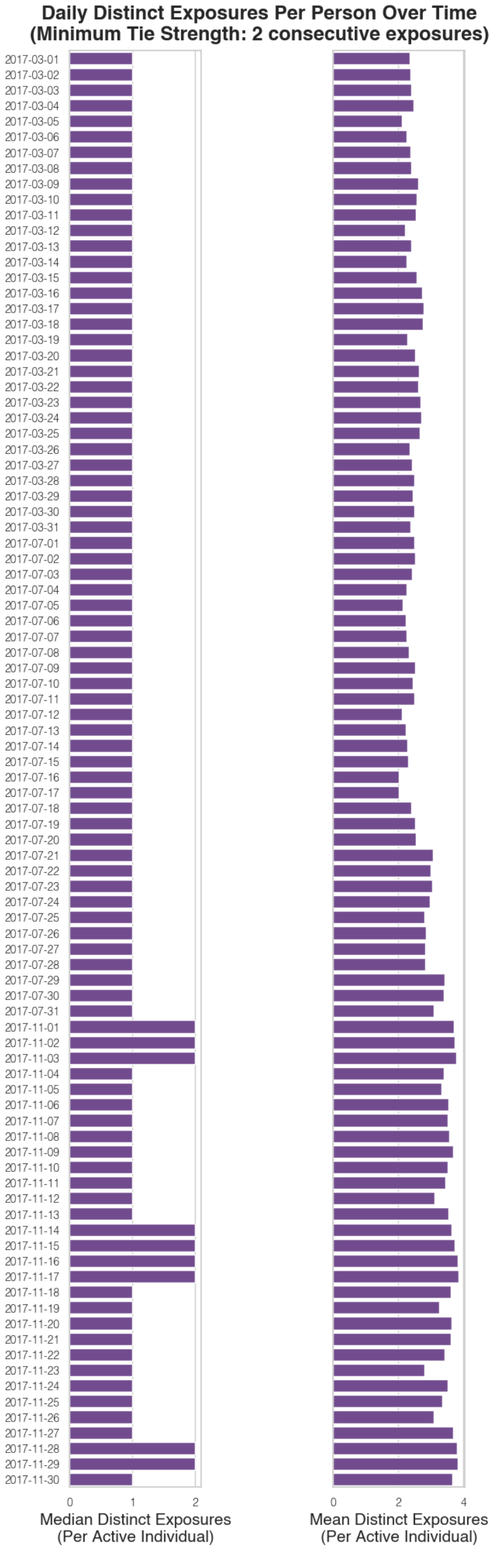

Supplementary Figure S50: Average number of distinct exposures over time (exposure length threshold: 2 consecutive exposures over five minute intervals). Mean/median distinct exposures per active individuals (i.e. nodes in the network) over the study observation period. Activity is defined as one or more exposures occurring on a given day.

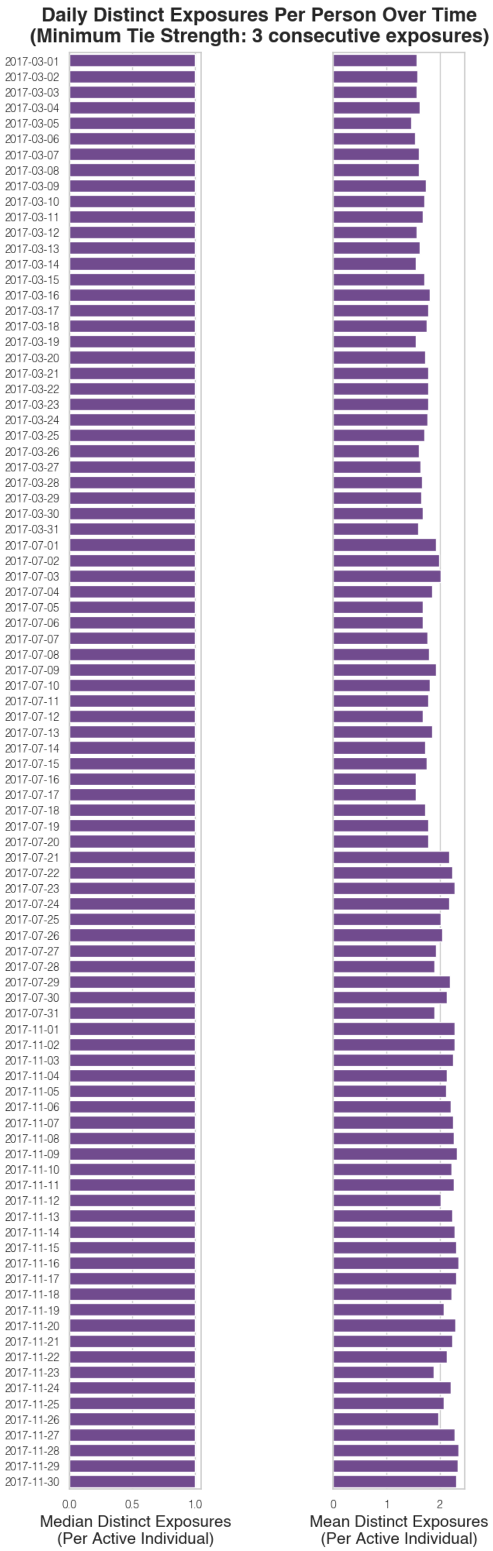

Supplementary Figure S51: Average number of distinct exposures over time (exposure length threshold: 3 consecutive exposures over five minute intervals). Mean/median distinct exposures per active individuals (i.e. nodes in the network) over the study observation period. Activity is defined as one or more exposures occurring on a given day.

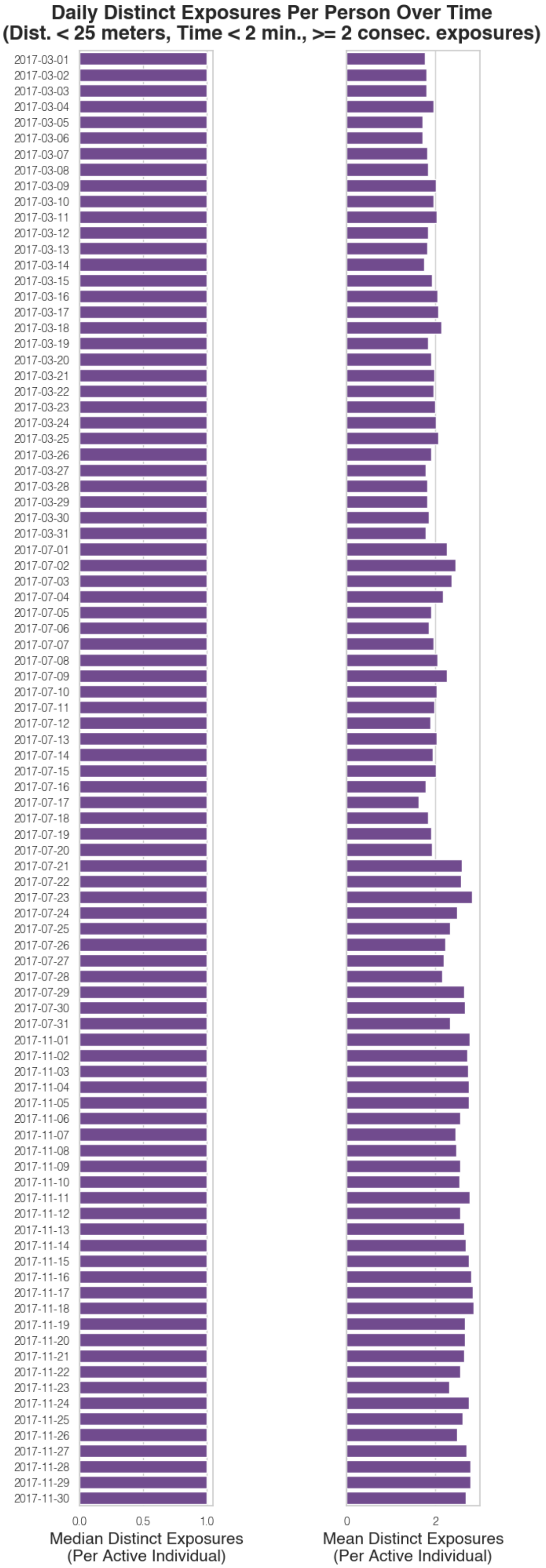

Supplementary Figure S52: Average number of distinct exposures over time (distance threshold: 25 meters, time threshold: 2 minutes, length threshold: 2 consecutive exposures of five minute intervals). Mean/median distinct exposures per active individuals (i.e. nodes in the network) over the study observation period. Activity is defined as one or more exposures occurring on a given day.

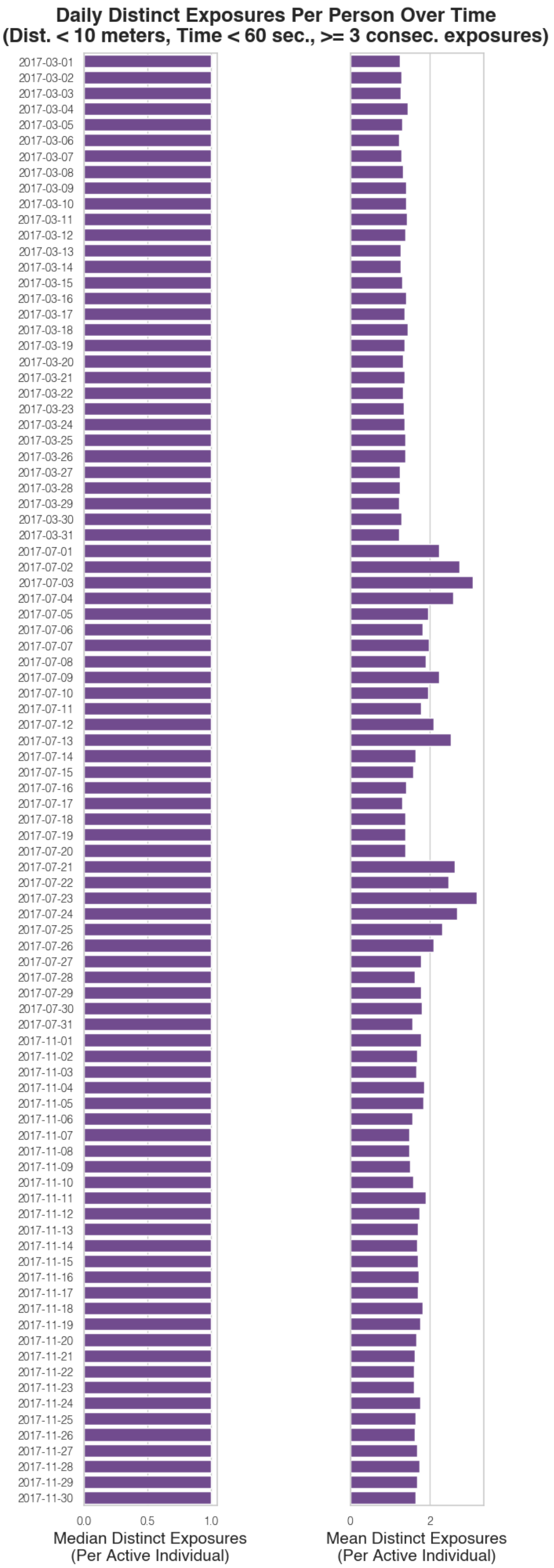

Supplementary Figure S53: Average number of distinct exposures over time (distance threshold: 10 meters, time threshold: 60 seconds, length threshold: 3 consecutive exposures of five minute intervals). Mean/median distinct exposures per active individuals (i.e. nodes in the network) over the study observation period. Activity is defined as one or more exposures occurring on a given day.

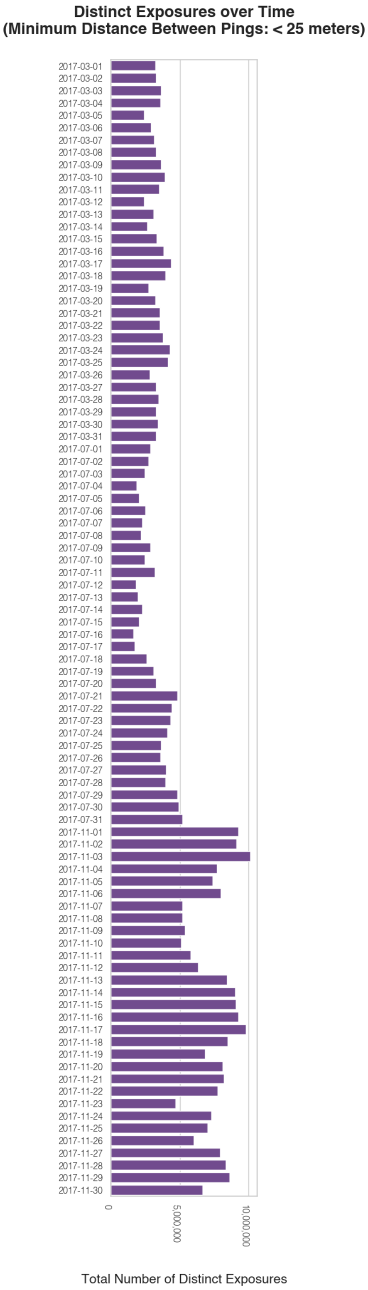

Supplementary Figure S54: Number of distinct exposures (distance threshold: 25 meters) over time across all individuals residing in the 382 MSAs.

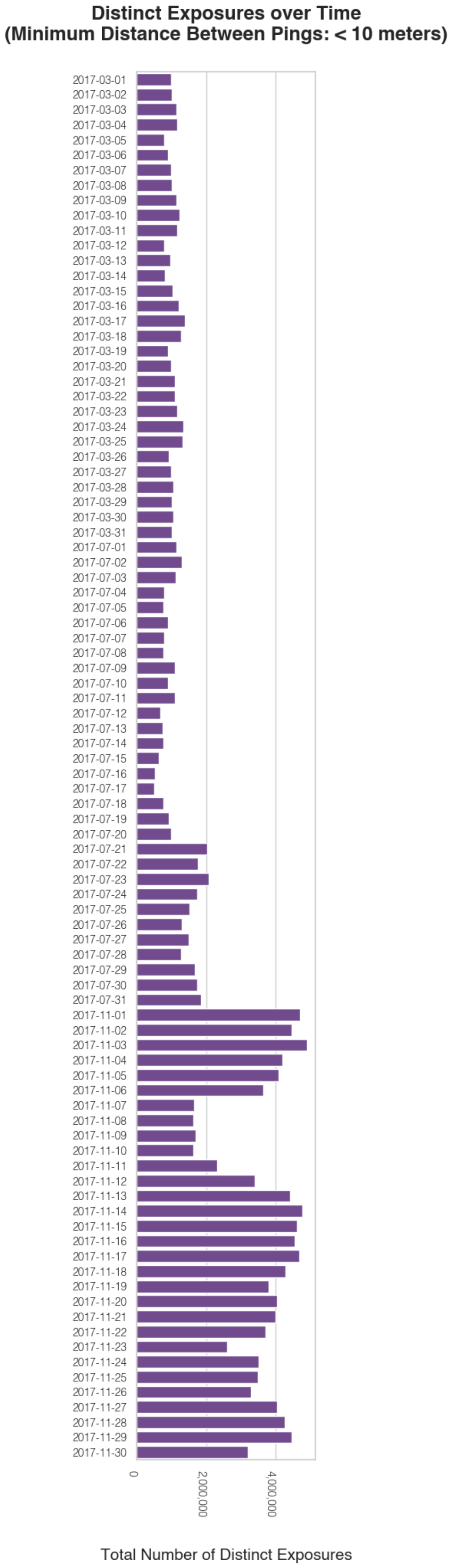

Supplementary Figure S55: Number of distinct exposures (distance threshold: 10 meters) over time across all individuals residing in the 382 MSAs.

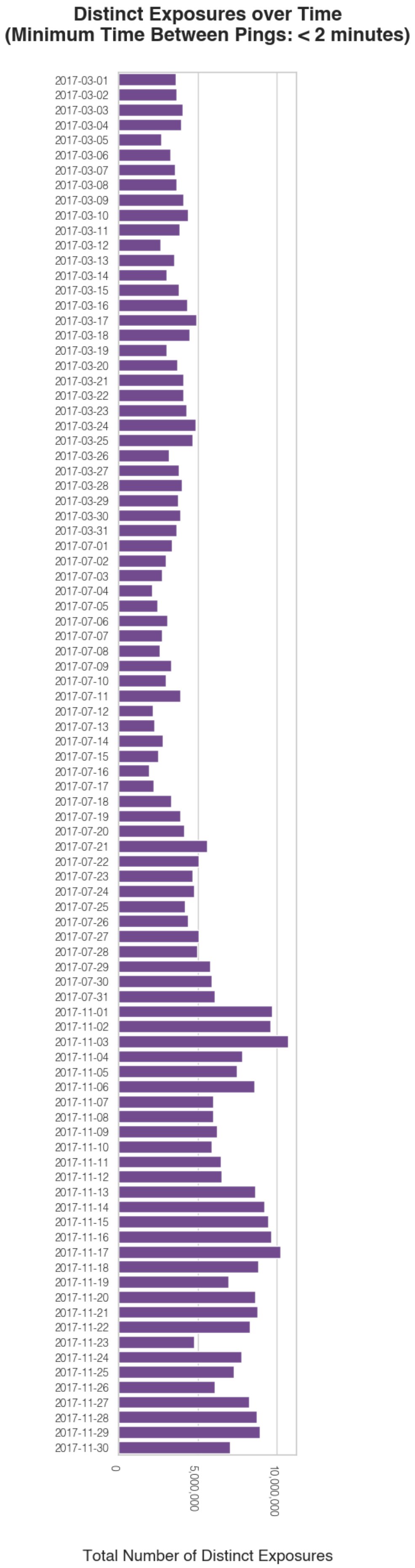

Supplementary Figure S56: Number of distinct exposures (time threshold: 2 minutes) over time across all individuals residing in the 382 MSAs.

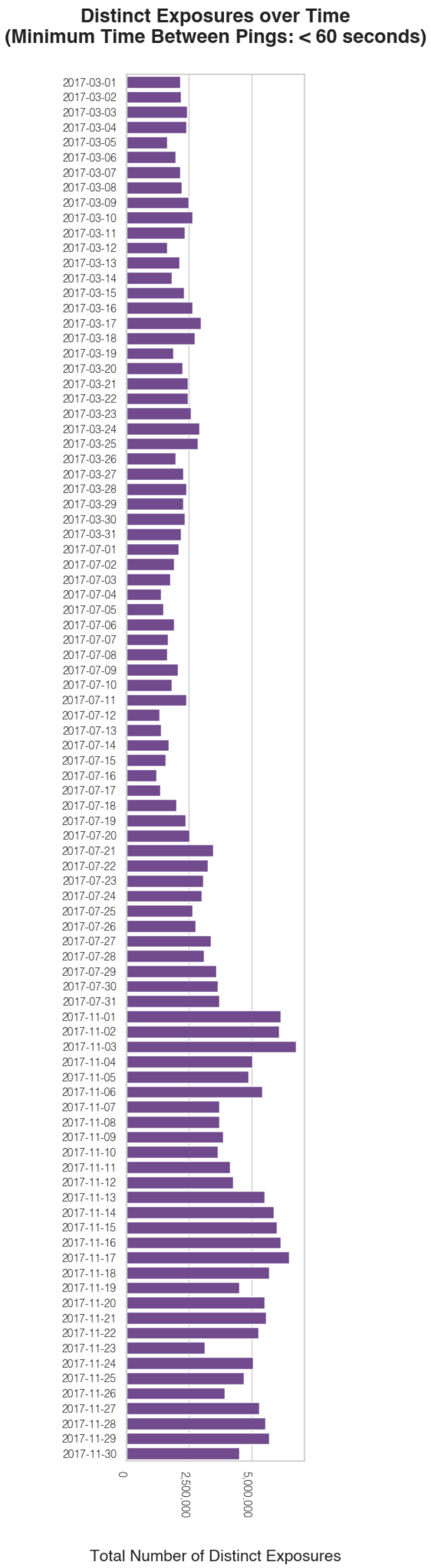

Supplementary Figure S57: Number of distinct exposures (time threshold: 60 seconds) over time across all individuals residing in the 382 MSAs.

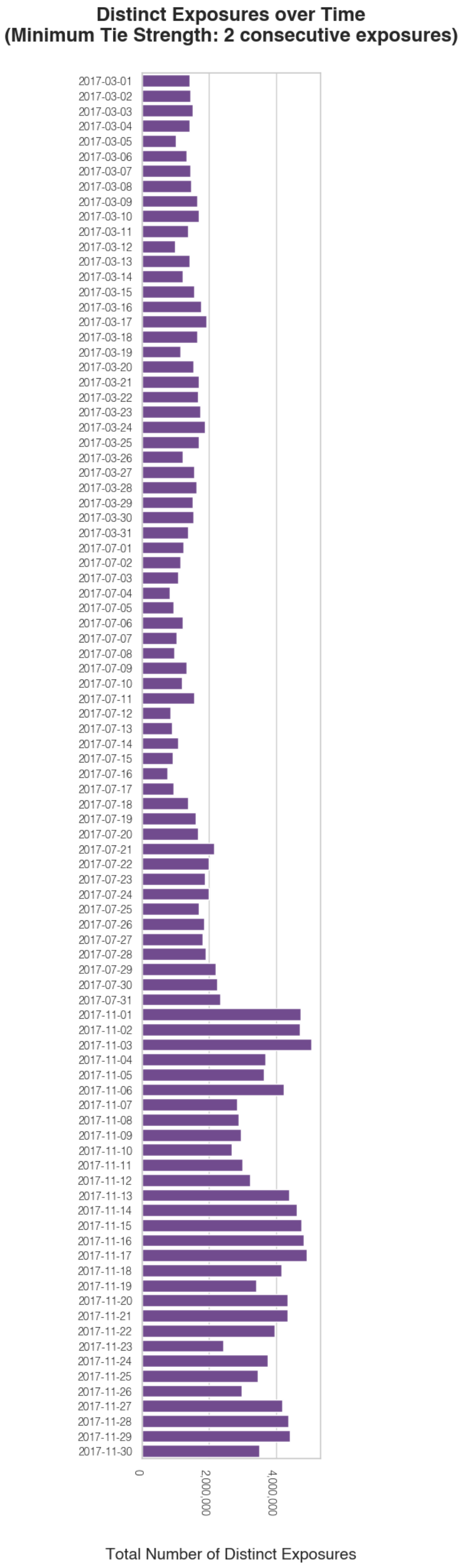

Supplementary Figure S58: Number of distinct exposures (exposure length threshold: 2 consecutive exposures over five minute intervals) over time across all individuals residing in the 382 MSAs.

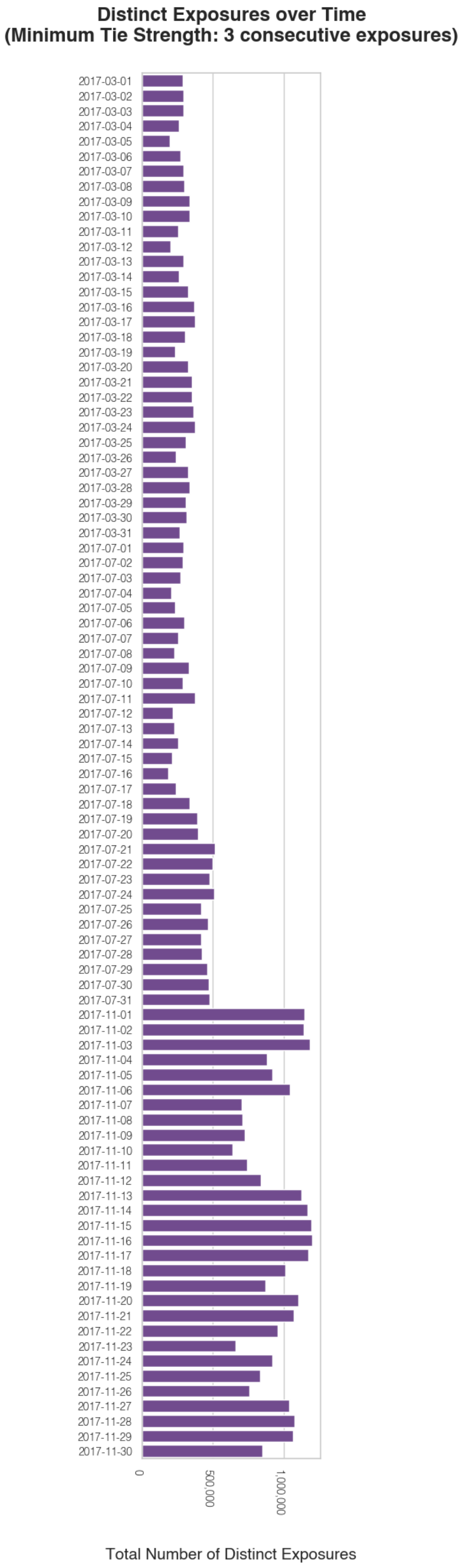

Supplementary Figure S59: Number of distinct exposures (exposure length threshold: 3 consecutive exposures over five minute intervals) over time across all individuals residing in the 382 MSAs.

Supplementary Table S21: Exposure Segregation and related variables (i.e. # exposures, mean SES, neighborhood sorting index, Gini index, population size, and bridging index) by MSA

MSA	Exposure Segregation	# Exposures	Mean SES	Neighborhood Sorting Index	Gini	Pop. Size	Bridging Index
Abilene, TX	0.44	561,896.00	1,245.47	0.63	0.21	170,516.00	0.79
Akron, OH	0.55	2,211,810.00	1,338.21	0.71	0.27	704,367.00	0.65
Albany, GA	0.40	355,999.00	1,077.89	0.47	0.26	151,293.00	0.79
Albany, OR	0.27	153,057.00	1,425.95	0.40	0.11	124,977.00	0.95
Albany-Schenectady-Troy, NY	0.40	2,058,079.00	1,651.16	0.62	0.18	882,130.00	0.81
Albuquerque, NM	0.35	1,979,325.00	1,316.79	0.59	0.19	912,897.00	0.78
Alexandria, LA	0.37	267,228.00	1,011.98	0.52	0.23	153,604.00	0.88
Allentown-Bethlehem-Easton, PA-NJ	0.44	2,629,311.00	1,647.92	0.62	0.19	838,081.00	0.79
Altoona, PA	0.14	199,365.00	799.18	0.59	0.08	123,175.00	0.83
Amarillo, TX	0.45	2,027,531.00	1,358.46	0.62	0.26	264,955.00	0.82
Ames, IA	0.22	178,625.00	1,255.36	0.37	0.19	97,260.00	0.97
Anchorage, AK	0.37	1,189,861.00	1,921.88	0.65	0.17	400,647.00	0.84
Ann Arbor, MI	0.42	984,188.00	2,087.55	0.69	0.19	369,208.00	0.76
Anniston-Oxford-Jacksonville, AL	0.27	498,621.00	949.79	0.43	0.19	114,664.00	0.96
Appleton, WI	0.28	798,727.00	1,201.77	0.60	0.10	236,058.00	0.94
Asheville, NC	0.36	1,449,906.00	1,634.66	0.44	0.18	455,255.00	0.88
Athens-Clarke County, GA	0.26	431,582.00	1,427.27	0.46	0.19	208,997.00	0.83
Atlanta-Sandy Springs-Roswell, GA	0.50	41,054,246.00	1,805.49	0.70	0.22	5,874,249.00	0.62
Atlantic City-Hammonton, NJ	0.55	788,439.00	1,993.59	0.79	0.27	266,328.00	0.59
Auburn-Opelika, AL	0.35	415,339.00	1,409.80	0.44	0.19	161,641.00	0.86
Augusta-Richmond County, GA-SC	0.39	1,698,887.00	1,257.25	0.57	0.21	600,006.00	0.79
Austin-Round Rock, TX	0.53	13,378,670.00	1,954.85	0.70	0.19	2,115,230.00	0.66
Bakersfield, CA	0.29	2,168,162.00	1,399.40	0.69	0.18	888,988.00	0.81
Baltimore-Columbia-Towson, MD	0.61	15,120,132.00	1,898.23	0.80	0.17	2,798,587.00	0.60
Bangor, ME	0.21	56,124.00	1,264.12	0.65	0.17	151,190.00	0.91
Barnstable Town, MA	0.32	556,457.00	2,483.81	0.43	0.18	213,482.00	0.90
Baton Rouge, LA	0.45	3,449,982.00	1,374.01	0.70	0.14	831,182.00	0.81
Battle Creek, MI	0.37	329,581.00	1,013.54	0.66	0.13	134,358.00	0.84
Bay City, MI	0.23	204,294.00	904.87	0.48	0.11	104,189.00	0.97
Beaumont-Port Arthur, TX	0.41	1,935,665.00	1,276.93	0.63	0.16	412,616.00	0.83
Beckley, WV	0.22	61,971.00	988.00	0.41	0.19	118,639.00	0.94
Bellingham, WA	0.18	300,129.00	1,946.31	0.35	0.16	221,650.00	0.95
Bend-Redmond, OR	0.38	257,079.00	1,983.21	0.53	0.17	186,807.00	0.92
Billings, MT	0.36	316,679.00	1,370.83	0.56	0.18	170,740.00	0.91
Binghamton, NY	0.21	325,247.00	1,125.73	0.56	0.16	241,609.00	0.90
Birmingham-Hoover, AL	0.56	7,522,699.00	1,392.29	0.73	0.26	1,149,685.00	0.63
Bismarck, ND	0.18	341,245.00	1,397.18	0.48	0.13	132,418.00	0.95
Blacksburg-Christiansburg-Radford, VA	0.33	260,509.00	1,340.74	0.57	0.17	182,692.00	0.82
Bloomington, IL	0.34	599,796.00	1,245.47	0.62	0.18	188,754.00	0.89
Bloomington, IN	0.35	405,186.00	1,517.20	0.47	0.22	167,513.00	0.91
Bloomsburg-Berwick, PA	0.19	80,784.00	994.00	0.64	0.14	83,924.00	0.94
Boise City, ID	0.41	1,299,521.00	1,584.28	0.60	0.16	710,080.00	0.81
Boston-Cambridge-Newton, MA-NH	0.38	22,163,903.00	2,655.52	0.63	0.16	4,844,597.00	0.70
Boulder, CO	0.36	744,121.00	2,570.91	0.57	0.21	324,073.00	0.84
Bowling Green, KY	0.43	541,630.00	1,227.13	0.59	0.22	174,962.00	0.93
Bremerton-Silverdale, WA	0.40	636,416.00	2,044.31	0.60	0.16	266,550.00	0.77
Bridgeport-Stamford-Norwalk, CT	0.51	3,009,778.00	2,840.03	0.79	0.32	943,457.00	0.53
Brownsville-Harlingen, TX	0.33	1,204,081.00	1,106.57	0.49	0.17	423,181.00	0.88
Brunswick, GA	0.55	345,233.00	1,939.59	0.65	0.33	117,728.00	0.56
Buffalo-Cheektowaga-Niagara Falls, NY	0.41	3,152,570.00	1,285.13	0.68	0.20	1,129,660.00	0.69
Burlington, NC	0.30	489,581.00	1,237.01	0.40	0.21	163,529.00	0.89
Burlington-South Burlington, VT	0.43	140,807.00	1,990.17	0.33	0.16	218,881.00	0.86
California-Lexington Park, MD	0.19	442,775.00	1,739.01	0.35	0.12	112,413.00	0.98
Canton-Massillon, OH	0.41	1,037,327.00	1,235.25	0.38	0.29	399,418.00	0.81
Cape Coral-Fort Myers, FL	0.39	6,067,007.00	1,990.09	0.57	0.27	739,506.00	0.74
Cape Girardeau, MO-IL	0.24	174,129.00	1,013.11	0.36	0.18	96,873.00	0.99
Carbondale-Marion, IL	0.21	182,895.00	855.65	0.37	0.13	125,065.00	0.99
Carson City, NV	0.33	124,126.00	1,700.73	0.59	0.18	54,608.00	0.98
Casper, WY	0.18	103,682.00	1,377.58	0.30	0.21	79,556.00	0.97
Cedar Rapids, IA	0.33	1,010,446.00	1,200.65	0.47	0.14	270,594.00	0.96
Chambersburg-Waynesboro, PA	0.26	158,476.00	1,080.40	0.48	0.10	154,487.00	0.96
Champaign-Urbana, IL	0.32	799,317.00	1,229.14	0.67	0.19	239,877.00	0.88
Charleston, WV	0.25	365,352.00	988.62	0.44	0.21	214,398.00	0.93
Charleston-North Charleston, SC	0.47	4,062,901.00	2,014.52	0.69	0.24	775,089.00	0.66
Charlotte-Concord-Gastonia, NC-SC	0.50	12,750,805.00	1,699.80	0.68	0.24	2,524,863.00	0.64
Charlottesville, VA	0.31	510,779.00	1,840.79	0.50	0.21	233,586.00	0.95
Chattanooga, TN-GA	0.46	2,432,138.00	1,376.30	0.62	0.18	556,081.00	0.86
Cheyenne, WY	0.33	234,335.00	1,450.51	0.67	0.15	98,460.00	0.96
Chicago-Naperville-Elgin, IL-IN-WI	0.44	61,552,971.00	1,943.77	0.66	0.21	9,520,784.00	0.68
Chico, CA	0.29	324,613.00	1,772.67	0.55	0.17	229,207.00	0.92
Cincinnati, OH-KY-IN	0.47	10,110,144.00	1,533.13	0.66	0.22	2,179,858.00	0.76
Clarksville, TN-KY	0.30	989,270.00	1,100.48	0.56	0.16	285,691.00	0.83
Cleveland, TN	0.23	421,419.00	1,072.17	0.33	0.15	122,082.00	0.92
Cleveland-Elyria, OH	0.54	6,830,481.00	1,385.15	0.68	0.25	2,058,549.00	0.63
Coeur d'Alene, ID	0.13	243,473.00	1,680.34	0.32	0.15	157,485.00	0.97
College Station-Bryan, TX	0.40	1,243,139.00	1,430.02	0.59	0.18	258,825.00	0.87
Colorado Springs, CO	0.42	2,666,493.00	1,758.07	0.64	0.16	725,438.00	0.72
Columbia, MO	0.36	425,486.00	1,194.35	0.50	0.20	178,523.00	0.86
Columbia, SC	0.42	3,047,549.00	1,390.00	0.59	0.21	825,110.00	0.81
Columbus, GA-AL	0.47	724,780.00	1,143.38	0.72	0.24	303,436.00	0.74
Columbus, IN	0.41	250,666.00	1,411.07	0.55	0.22	82,429.00	0.94
Columbus, OH	0.55	9,849,191.00	1,623.39	0.71	0.23	2,082,475.00	0.69

Continued on next page

Supplementary Table S21: (cont'd) Exposure Segregation and related variables (i.e. # exposures, mean SES, neighborhood sorting index, Gini index, population size, and bridging index by MSA

MSA	Exposure Segregation	# Exposures	Mean SES	Neighborhood Sorting Index	Gini	Pop. Size	Bridging Index
Corpus Christi, TX	0.50	2,288,424.00	1,487.67	0.72	0.16	453,684.00	0.79
Corvallis, OR	0.24	104,179.00	1,936.53	0.48	0.15	91,567.00	0.97
Crestview-Fort Walton Beach-Destin, FL	0.43	1,868,711.00	1,852.07	0.53	0.24	271,959.00	0.76
Cumberland, MD-WV	0.26	30,347.00	936.53	0.51	0.14	98,566.00	0.98
Dallas-Fort Worth-Arlington, TX	0.51	48,228,424.00	1,996.27	0.73	0.22	7,407,944.00	0.62
Dalton, GA	0.14	235,701.00	876.66	0.89	0.14	143,872.00	0.77
Danville, IL	0.24	70,716.00	693.62	0.73	0.05	77,776.00	0.96
Daphne-Fairhope-Foley, AL	0.34	1,066,095.00	1,602.47	0.41	0.16	212,619.00	0.96
Davenport-Moline-Rock Island, IA-IL	0.45	945,921.00	1,287.37	0.70	0.22	381,854.00	0.76
Dayton, OH	0.49	2,617,342.00	1,278.37	0.70	0.25	803,713.00	0.73
Decatur, AL	0.29	403,755.00	981.61	0.42	0.12	151,888.00	0.94
Decatur, IL	0.35	233,031.00	919.63	0.76	0.15	105,533.00	0.86
Deltona-Daytona Beach-Ormond Beach, FL	0.33	5,092,950.00	1,686.87	0.46	0.18	648,117.00	0.82
Denver-Aurora-Lakewood, CO	0.35	11,589,449.00	2,312.40	0.67	0.17	2,892,979.00	0.77
Des Moines-West Des Moines, IA	0.46	1,966,610.00	1,577.32	0.53	0.22	645,100.00	0.76
Detroit-Warren-Dearborn, MI	0.57	15,495,989.00	1,554.43	0.77	0.26	4,321,704.00	0.49
Dothan, AL	0.33	466,788.00	1,142.03	0.45	0.23	147,923.00	0.89
Dover, DE	0.26	462,113.00	1,471.35	0.34	0.13	176,445.00	0.93
Dubuque, IA	0.36	201,954.00	1,313.10	0.57	0.18	97,009.00	0.86
Duluth, MN-WI	0.44	458,229.00	1,373.15	0.67	0.21	278,659.00	0.74
Durham-Chapel Hill, NC	0.44	2,019,082.00	1,713.50	0.57	0.19	566,491.00	0.83
East Stroudsburg, PA	0.24	452,426.00	1,526.37	0.39	0.12	168,089.00	0.95
Eau Claire, WI	0.18	377,072.00	1,114.77	0.41	0.11	167,436.00	0.95
El Centro, CA	0.18	271,797.00	1,340.14	0.69	0.12	181,574.00	0.96
El Paso, TX	0.32	1,667,796.00	1,157.84	0.60	0.17	845,145.00	0.76
Elizabethtown-Fort Knox, KY	0.19	245,608.00	1,241.61	0.24	0.22	150,531.00	0.91
Elkhart-Goshen, IN	0.37	567,181.00	1,186.01	0.41	0.17	204,310.00	0.89
Elmira, NY	0.36	121,786.00	1,173.73	0.58	0.20	84,874.00	0.78
Enid, OK	0.38	320,003.00	1,102.25	0.59	0.24	61,492.00	0.89
Erie, PA	0.34	571,583.00	1,129.58	0.61	0.21	273,892.00	0.85
Eugene, OR	0.29	514,167.00	1,600.88	0.49	0.15	375,617.00	0.93
Evansville, IN-KY	0.47	751,625.00	1,240.07	0.60	0.26	314,960.00	0.80
Fairbanks, AK	0.16	60,754.00	1,619.53	0.36	0.12	99,725.00	0.99
Fargo, ND-MN	0.30	816,028.00	1,333.79	0.52	0.12	241,619.00	0.92
Farmington, NM	0.28	67,876.00	1,265.57	0.52	0.17	126,902.00	0.98
Fayetteville, NC	0.27	1,253,869.00	1,082.27	0.48	0.19	385,380.00	0.90
Fayetteville-Springdale-Rogers, AR-MO	0.43	1,854,285.00	1,371.30	0.60	0.18	538,412.00	0.91
Flagstaff, AZ	0.31	184,120.00	2,012.92	0.42	0.15	141,107.00	0.96
Flint, MI	0.50	981,799.00	1,063.23	0.72	0.22	407,673.00	0.58
Florence, SC	0.37	312,804.00	1,335.06	0.49	0.21	205,546.00	0.93
Florence-Muscle Shoals, AL	0.22	331,068.00	912.14	0.50	0.14	147,100.00	0.97
Fond du Lac, WI	0.19	220,305.00	873.95	0.47	0.09	102,371.00	0.92
Fort Collins, CO	0.28	926,669.00	1,916.57	0.43	0.13	343,993.00	0.91
Fort Smith, AR-OK	0.32	638,594.00	887.85	0.60	0.16	281,990.00	0.93
Fort Wayne, IN	0.50	1,475,260.00	1,342.98	0.69	0.25	434,001.00	0.66
Fresno, CA	0.35	2,137,796.00	1,471.73	0.69	0.18	986,542.00	0.73
Gadsden, AL	0.30	465,122.00	894.00	0.59	0.19	102,937.00	0.89
Gainesville, FL	0.35	1,165,180.00	1,593.03	0.56	0.22	284,685.00	0.82
Gainesville, GA	0.34	758,361.00	1,756.64	0.32	0.22	199,439.00	0.85
Gettysburg, PA	0.22	205,160.00	1,410.01	0.34	0.10	102,367.00	0.99
Glens Falls, NY	0.27	131,519.00	1,486.70	0.59	0.21	125,917.00	0.84
Goldsboro, NC	0.27	275,443.00	1,088.76	0.28	0.18	123,257.00	0.87
Grand Forks, ND-MN	0.33	184,078.00	1,228.24	0.57	0.21	102,277.00	0.98
Grand Island, NE	0.25	236,404.00	1,138.90	0.40	0.14	84,862.00	1.00
Grand Junction, CO	0.32	248,844.00	1,410.36	0.59	0.16	151,406.00	0.88
Grand Rapids-Wyoming, MI	0.40	2,808,054.00	1,540.98	0.65	0.16	1,060,326.00	0.71
Grants Pass, OR	0.19	84,482.00	1,601.76	0.38	0.14	86,653.00	1.00
Great Falls, MT	0.27	156,642.00	1,210.84	0.51	0.13	81,604.00	1.00
Greeley, CO	0.44	749,425.00	1,912.90	0.57	0.14	305,274.00	0.79
Green Bay, WI	0.44	1,141,954.00	1,416.76	0.71	0.21	319,786.00	0.86
Greensboro-High Point, NC	0.48	2,269,305.00	1,226.69	0.67	0.26	763,486.00	0.69
Greenville, NC	0.36	702,454.00	1,259.95	0.34	0.22	178,617.00	0.94
Greenville-Anderson-Mauldin, SC	0.46	2,888,574.00	1,383.12	0.56	0.21	895,422.00	0.82
Gulfport-Biloxi-Pascagoula, MS	0.35	1,349,098.00	1,223.72	0.49	0.18	394,322.00	0.92
Hagerstown-Martinsburg, MD-WV	0.32	599,583.00	1,349.39	0.54	0.16	265,295.00	0.96
Hammond, LA	0.31	354,092.00	1,182.56	0.38	0.14	132,322.00	0.94
Hanford-Corcoran, CA	0.22	253,730.00	1,343.94	0.62	0.15	149,696.00	0.96
Harrisburg-Carlisle, PA	0.39	1,555,132.00	1,467.19	0.54	0.19	571,101.00	0.78
Harrisonburg, VA	0.26	290,643.00	1,278.25	0.42	0.16	134,220.00	0.95
Hartford-West Hartford-East Hartford, CT	0.43	2,241,050.00	1,710.35	0.66	0.18	1,206,719.00	0.77
Hattiesburg, MS	0.37	311,274.00	1,156.01	0.70	0.16	148,719.00	0.83
Hickory-Lenoir-Morganton, NC	0.41	640,647.00	1,244.75	0.47	0.18	367,004.00	0.91
Hilton Head Island-Bluffton-Beaufort, SC	0.39	732,993.00	2,115.09	0.53	0.21	214,890.00	0.82
Hinesville, GA	0.20	138,926.00	1,134.33	0.29	0.13	80,518.00	0.97
Homosassa Springs, FL	0.30	528,334.00	1,417.05	0.46	0.21	145,512.00	0.94
Hot Springs, AR	0.34	274,972.00	1,162.56	0.39	0.23	98,444.00	0.89
Houma-Thibodaux, LA	0.29	511,209.00	1,159.51	0.65	0.12	209,893.00	0.83
Houston-The Woodlands-Sugar Land, TX	0.47	63,151,024.00	1,866.72	0.72	0.22	6,905,695.00	0.66
Huntington-Ashland, WV-KY-OH	0.34	860,612.00	1,069.95	0.49	0.18	355,582.00	0.87
Huntsville, AL	0.45	1,623,341.00	1,313.85	0.66	0.20	455,741.00	0.81
Idaho Falls, ID	0.25	212,821.00	1,219.23	0.54	0.14	145,792.00	0.95
Indianapolis-Carmel-Anderson, IN	0.52	10,182,520.00	1,466.08	0.68	0.24	2,026,723.00	0.64

Continued on next page

Supplementary Table S21: (cont'd) Exposure Segregation and related variables (i.e. # exposures, mean SES, neighborhood sorting index, Gini index, population size, and bridging index by MSA

MSA	Exposure Segregation	# Exposures	Mean SES	Neighborhood Sorting Index	Gini	Pop. Size	Bridging Index
Iowa City, IA	0.36	473,387.00	1,499.89	0.44	0.20	171,470.00	0.97
Ithaca, NY	0.23	130,902.00	1,607.18	0.45	0.14	102,678.00	0.95
Jackson, MI	0.40	294,167.00	1,049.19	0.67	0.15	158,690.00	0.96
Jackson, MS	0.58	1,725,331.00	1,409.20	0.72	0.24	581,552.00	0.68
Jackson, TN	0.40	324,665.00	1,075.77	0.74	0.16	129,186.00	0.82
Jacksonville, FL	0.49	10,861,594.00	1,742.93	0.61	0.24	1,504,841.00	0.64
Jacksonville, NC	0.30	544,185.00	1,162.30	0.45	0.17	194,838.00	0.90
Janesville-Beloit, WI	0.25	328,187.00	1,002.54	0.65	0.10	162,320.00	0.76
Jefferson City, MO	0.24	349,139.00	1,010.56	0.45	0.16	151,298.00	0.96
Johnson City, TN	0.33	357,946.00	1,075.54	0.55	0.17	201,844.00	0.84
Johnstown, PA	0.20	232,779.00	741.42	0.62	0.11	133,054.00	0.98
Jonesboro, AR	0.36	308,755.00	1,106.28	0.64	0.17	131,158.00	0.94
Joplin, MO	0.21	356,202.00	864.53	0.41	0.12	178,330.00	0.97
Kahului-Wailuku-Lahaina, HI	0.22	246,346.00	3,043.60	0.37	0.16	166,491.00	0.97
Kalamazoo-Portage, MI	0.37	910,106.00	1,412.34	0.58	0.14	338,347.00	0.88
Kankakee, IL	0.30	374,176.00	1,249.33	0.70	0.10	110,544.00	0.81
Kansas City, MO-KS	0.54	8,835,941.00	1,541.78	0.75	0.25	2,127,259.00	0.64
Kennewick-Richland, WA	0.37	373,182.00	1,575.53	0.61	0.15	290,570.00	0.88
Killeen-Temple, TX	0.37	1,587,760.00	1,116.71	0.58	0.16	443,653.00	0.87
Kingsport-Bristol-Bristol, TN-VA	0.29	373,431.00	1,097.78	0.46	0.17	306,253.00	0.93
Kingston, NY	0.31	290,633.00	1,750.29	0.47	0.13	178,723.00	0.94
Knoxville, TN	0.43	2,704,521.00	1,467.11	0.60	0.23	875,797.00	0.76
Kokomo, IN	0.31	288,583.00	944.72	0.55	0.16	82,311.00	0.88
La Crosse-Onalaska, WI-MN	0.17	200,045.00	1,144.24	0.54	0.09	136,778.00	0.97
Lafayette, LA	0.39	1,650,845.00	1,126.34	0.67	0.16	490,107.00	0.76
Lafayette-West Lafayette, IN	0.33	768,405.00	1,226.26	0.57	0.16	220,337.00	0.88
Lake Charles, LA	0.27	207,541.00	1,286.99	0.62	0.13	209,256.00	0.88
Lake Havasu City-Kingman, AZ	0.30	321,719.00	1,255.77	0.59	0.18	207,114.00	0.83
Lakeland-Winter Haven, FL	0.31	4,246,971.00	1,435.22	0.43	0.18	685,830.00	0.90
Lancaster, PA	0.28	1,111,168.00	1,304.06	0.54	0.11	541,054.00	0.86
Lansing-East Lansing, MI	0.42	1,146,004.00	1,249.29	0.65	0.19	480,353.00	0.80
Laredo, TX	0.55	682,291.00	1,308.82	0.76	0.18	273,982.00	0.72
Las Cruces, NM	0.27	340,688.00	1,194.16	0.44	0.18	216,186.00	0.97
Las Vegas-Henderson-Paradise, NV	0.27	10,258,483.00	1,703.98	0.51	0.20	2,183,310.00	0.82
Lawrence, KS	0.19	423,826.00	1,417.50	0.47	0.20	120,629.00	0.94
Lawton, OK	0.30	337,308.00	933.32	0.58	0.21	127,589.00	0.88
Lebanon, PA	0.43	276,347.00	1,155.40	0.70	0.13	139,566.00	0.88
Lewiston, ID-WA	0.20	43,087.00	1,309.23	0.23	0.13	62,881.00	0.99
Lewiston-Auburn, ME	0.21	110,078.00	1,111.21	0.53	0.07	107,569.00	0.98
Lexington-Fayette, KY	0.42	2,391,314.00	1,321.56	0.62	0.21	512,732.00	0.85
Lima, OH	0.35	228,949.00	983.20	0.60	0.16	103,069.00	0.95
Lincoln, NE	0.35	1,808,658.00	1,395.51	0.57	0.16	331,179.00	0.87
Little Rock-North Little Rock-Conway, AR	0.51	3,070,717.00	1,192.95	0.72	0.20	737,991.00	0.77
Logan, UT-ID	0.20	154,902.00	1,210.21	0.60	0.10	138,052.00	0.92
Longview, TX	0.35	262,120.00	1,243.45	0.61	0.18	218,594.00	0.86
Longview, WA	0.38	173,605.00	1,459.50	0.52	0.18	106,900.00	1.00
Los Angeles-Long Beach-Anaheim, CA	0.44	110,526,499.00	2,970.24	0.75	0.20	13,298,709.00	0.66
Louisville/Jefferson County, KY-IN	0.51	4,567,106.00	1,436.51	0.67	0.23	1,292,809.00	0.71
Lubbock, TX	0.45	1,549,243.00	1,381.12	0.55	0.22	316,588.00	0.83
Lynchburg, VA	0.31	496,432.00	1,201.66	0.58	0.18	261,954.00	0.84
Macon-Bibb County, GA	0.46	616,989.00	1,232.02	0.58	0.26	229,081.00	0.81
Madera, CA	0.30	249,720.00	1,396.67	0.47	0.14	155,904.00	0.96
Madison, WI	0.37	1,737,217.00	1,628.86	0.60	0.17	654,577.00	0.89
Manchester-Nashua, NH	0.46	929,901.00	2,027.43	0.69	0.18	413,157.00	0.88
Manhattan, KS	0.31	268,899.00	1,285.52	0.38	0.19	97,954.00	0.99
Mankato-North Mankato, MN	0.26	274,393.00	1,407.12	0.35	0.13	100,945.00	0.97
Mansfield, OH	0.30	203,318.00	872.54	0.50	0.12	120,543.00	0.88
McAllen-Edinburg-Mission, TX	0.36	2,672,266.00	1,165.08	0.45	0.22	858,323.00	0.87
Medford, OR	0.32	250,898.00	1,584.27	0.48	0.14	216,761.00	0.93
Memphis, TN-MS-AR	0.56	5,217,305.00	1,409.82	0.74	0.27	1,347,576.00	0.58
Merced, CA	0.24	471,172.00	1,489.37	0.57	0.13	271,340.00	0.92
Miami-Fort Lauderdale-West Palm Beach, FL	0.44	147,998,127.00	2,642.33	0.67	0.28	6,149,687.00	0.70
Michigan City-La Porte, IN	0.31	262,677.00	1,150.07	0.58	0.16	109,911.00	0.93
Midland, MI	0.35	156,234.00	1,161.45	0.63	0.18	83,245.00	0.96
Midland, TX	0.33	821,156.00	2,759.87	0.61	0.19	170,948.00	0.91
Milwaukee-Waukesha-West Allis, WI	0.60	5,452,737.00	1,428.80	0.77	0.24	1,575,151.00	0.63
Minneapolis-St. Paul-Bloomington, MN-WI	0.41	17,181,042.00	1,970.38	0.56	0.18	3,592,669.00	0.78
Missoula, MT	0.20	144,440.00	1,486.18	0.49	0.17	117,863.00	0.96
Mobile, AL	0.28	1,700,477.00	1,102.96	0.52	0.16	414,515.00	0.85
Modesto, CA	0.20	1,396,841.00	1,673.04	0.53	0.12	545,267.00	0.91
Monroe, LA	0.39	420,225.00	1,057.93	0.56	0.25	178,211.00	0.81
Monroe, MI	0.20	298,001.00	1,123.01	0.45	0.08	149,592.00	0.99
Montgomery, AL	0.47	933,055.00	1,116.61	0.73	0.18	374,042.00	0.71
Morgantown, WV	0.28	144,020.00	1,375.31	0.53	0.22	139,739.00	0.99
Morristown, TN	0.35	127,639.00	1,123.38	0.35	0.16	117,843.00	0.95
Mount Vernon-Anacortes, WA	0.29	164,737.00	1,848.65	0.46	0.13	126,026.00	0.99
Muncie, IN	0.34	325,604.00	951.96	0.58	0.19	115,389.00	0.91
Muskegon, MI	0.32	338,931.00	1,059.59	0.50	0.15	173,656.00	0.86
Myrtle Beach-Conway-North Myrtle Beach, SC-NC	0.24	1,937,451.00	1,614.84	0.27	0.22	463,386.00	0.87
Napa, CA	0.19	813,681.00	3,152.52	0.45	0.18	140,386.00	0.95
Naples-Immokalee-Marco Island, FL	0.45	3,239,165.00	3,865.02	0.70	0.36	372,345.00	0.77
Nashville-Davidson–Murfreesboro–Franklin, TN	0.50	10,766,763.00	1,845.97	0.74	0.22	1,900,584.00	0.62

Continued on next page

Supplementary Table S21: (cont'd) Exposure Segregation and related variables (i.e. # exposures, mean SES, neighborhood sorting index, Gini index, population size, and bridging index by MSA

MSA	Exposure Segregation	# Exposures	Mean SES	Neighborhood Sorting Index	Gini	Pop. Size	Bridging Index
New Bern, NC	0.32	309,000.00	1,270.00	0.47	0.19	125,010.00	0.89
New Haven-Milford, CT	0.37	2,382,587.00	1,669.24	0.53	0.19	857,794.00	0.76
New Orleans-Metairie, LA	0.40	6,489,654.00	1,556.45	0.68	0.16	1,270,465.00	0.81
New York-Newark-Jersey City, NY-NJ-PA	0.40	168,755,438.00	2,597.55	0.69	0.21	19,998,951.00	0.57
Niles-Benton Harbor, MI	0.51	301,883.00	1,248.76	0.78	0.19	154,362.00	0.80
North Port-Sarasota-Bradenton, FL	0.40	6,601,216.00	2,284.53	0.61	0.27	805,139.00	0.73
Norwich-New London, CT	0.27	286,633.00	1,478.91	0.60	0.15	267,826.00	0.87
Ocala, FL	0.30	1,669,292.00	1,370.81	0.39	0.21	353,717.00	0.90
Odessa, TX	0.28	687,081.00	2,187.43	0.54	0.13	157,173.00	0.88
Ogden-Clearfield, UT	0.38	1,513,318.00	1,609.07	0.63	0.15	664,589.00	0.84
Oklahoma City, OK	0.46	11,029,080.00	1,417.61	0.63	0.27	1,383,249.00	0.74
Olympia-Tumwater, WA	0.19	644,378.00	1,873.77	0.37	0.14	280,289.00	0.98
Omaha-Council Bluffs, NE-IA	0.49	3,944,289.00	1,583.10	0.64	0.22	932,217.00	0.69
Orlando-Kissimmee-Sanford, FL	0.42	25,094,242.00	1,870.76	0.57	0.21	2,512,917.00	0.72
Oshkosh-Neenah, WI	0.36	624,252.00	1,196.65	0.58	0.20	170,375.00	0.90
Owensboro, KY	0.36	203,069.00	1,064.78	0.48	0.21	118,543.00	0.95
Oxnard-Thousand Oaks-Ventura, CA	0.34	3,449,664.00	3,029.11	0.67	0.17	850,802.00	0.81
Palm Bay-Melbourne-Titusville, FL	0.43	4,331,998.00	1,826.84	0.52	0.20	588,265.00	0.77
Panama City, FL	0.28	1,089,950.00	2,071.06	0.40	0.20	200,168.00	0.93
Parkersburg-Vienna, WV	0.25	140,256.00	1,096.22	0.37	0.18	90,873.00	0.95
Pensacola-Ferry Pass-Brent, FL	0.42	3,672,000.00	1,405.47	0.55	0.21	487,327.00	0.78
Peoria, IL	0.46	914,882.00	1,178.67	0.63	0.22	371,810.00	0.80
Philadelphia-Camden-Wilmington, PA-NJ-DE-MD	0.53	24,822,104.00	1,802.57	0.74	0.20	6,078,451.00	0.61
Phoenix-Mesa-Scottsdale, AZ	0.48	17,152,709.00	1,746.59	0.75	0.19	4,761,694.00	0.61
Pine Bluff, AR	0.15	171,555.00	759.22	0.52	0.10	90,923.00	0.95
Pittsburgh, PA	0.47	7,756,479.00	1,348.23	0.70	0.24	2,330,283.00	0.72
Pittsfield, MA	0.37	128,317.00	1,477.61	0.52	0.15	126,485.00	0.86
Pocatello, ID	0.28	116,547.00	1,158.04	0.60	0.18	85,641.00	0.97
Port St. Lucie, FL	0.44	4,896,670.00	2,082.29	0.62	0.23	473,192.00	0.72
Portland-South Portland, ME	0.30	493,445.00	1,896.31	0.52	0.16	532,280.00	0.80
Portland-Vancouver-Hillsboro, OR-WA	0.34	6,614,427.00	2,016.66	0.58	0.16	2,456,462.00	0.86
Prescott, AZ	0.38	384,045.00	1,627.89	0.60	0.17	228,055.00	0.88
Providence-Warwick, RI-MA	0.40	5,763,343.00	1,744.07	0.63	0.16	1,617,057.00	0.79
Provo-Orem, UT	0.32	1,219,235.00	1,546.11	0.66	0.13	617,751.00	0.84
Pueblo, CO	0.36	443,458.00	1,270.67	0.65	0.18	166,426.00	0.85
Punta Gorda, FL	0.34	1,184,701.00	1,829.41	0.60	0.21	181,537.00	0.84
Racine, WI	0.42	548,195.00	1,383.27	0.63	0.16	195,949.00	0.79
Raleigh, NC	0.46	8,986,021.00	1,697.93	0.62	0.16	1,334,342.00	0.79
Rapid City, SD	0.32	250,301.00	1,441.12	0.41	0.20	146,869.00	0.88
Reading, PA	0.49	1,036,022.00	1,344.18	0.68	0.18	417,524.00	0.71
Redding, CA	0.23	258,289.00	1,573.52	0.43	0.19	179,539.00	0.99
Reno, NV	0.47	1,550,552.00	1,953.99	0.63	0.18	461,336.00	0.70
Richmond, VA	0.49	4,524,531.00	1,626.59	0.71	0.20	1,292,911.00	0.68
Riverside-San Bernardino-Ontario, CA	0.43	19,908,134.00	2,103.34	0.70	0.17	4,570,427.00	0.68
Roanoke, VA	0.38	811,899.00	1,291.22	0.58	0.20	313,488.00	0.80
Rochester, MN	0.33	648,812.00	1,540.01	0.58	0.16	217,828.00	0.94
Rochester, NY	0.45	2,870,914.00	1,536.09	0.66	0.19	1,071,589.00	0.77
Rockford, IL	0.40	946,786.00	1,225.98	0.67	0.18	338,252.00	0.72
Rocky Mount, NC	0.33	366,999.00	977.64	0.55	0.16	146,769.00	0.81
Rome, GA	0.36	264,276.00	1,078.32	0.39	0.16	97,427.00	0.99
Sacramento–Roseville–Arden-Arcade, CA	0.39	7,101,248.00	2,048.69	0.66	0.15	2,320,381.00	0.70
Saginaw, MI	0.37	355,021.00	927.43	0.70	0.18	191,996.00	0.77
Salem, OR	0.27	670,775.00	1,603.26	0.45	0.12	424,968.00	0.94
Salinas, CA	0.36	952,169.00	2,642.69	0.65	0.17	435,477.00	0.77
Salisbury, MD-DE	0.48	875,922.00	1,462.24	0.68	0.14	404,067.00	0.78
Salt Lake City, UT	0.33	3,468,862.00	1,763.34	0.60	0.15	1,205,238.00	0.77
San Angelo, TX	0.34	380,590.00	1,321.06	0.66	0.14	119,200.00	0.83
San Antonio-New Braunfels, TX	0.53	14,354,046.00	1,596.19	0.70	0.20	2,474,274.00	0.64
San Diego-Carlsbad, CA	0.42	13,807,983.00	2,854.26	0.73	0.20	3,325,468.00	0.71
San Francisco-Oakland-Hayward, CA	0.41	37,492,367.00	3,925.90	0.71	0.21	4,710,693.00	0.68
San Jose-Sunnyvale-Santa Clara, CA	0.37	8,012,471.00	3,766.32	0.78	0.16	1,993,582.00	0.68
San Luis Obispo-Paso Robles-Arroyo Grande, CA	0.22	656,686.00	2,601.39	0.47	0.13	282,838.00	0.94
Santa Cruz-Watsonville, CA	0.27	611,337.00	3,306.01	0.52	0.13	275,105.00	0.85
Santa Fe, NM	0.45	182,572.00	2,075.86	0.61	0.23	149,617.00	0.91
Santa Maria-Santa Barbara, CA	0.52	1,124,975.00	3,039.10	0.75	0.28	445,606.00	0.59
Santa Rosa, CA	0.22	1,144,462.00	2,793.94	0.54	0.11	503,246.00	0.92
Savannah, GA	0.39	1,601,410.00	1,599.94	0.57	0.19	386,337.00	0.83
Scranton–Wilkes-Barre–Hazleton, PA	0.30	1,064,769.00	1,077.93	0.62	0.16	555,645.00	0.90
Seattle-Tacoma-Bellevue, WA	0.44	18,136,495.00	2,474.85	0.64	0.20	3,884,469.00	0.73
Sebastian-Vero Beach, FL	0.52	1,148,601.00	2,259.56	0.71	0.31	154,314.00	0.78
Sebring, FL	0.26	352,266.00	1,291.88	0.31	0.21	104,060.00	0.98
Sheboygan, WI	0.35	220,669.00	1,204.87	0.58	0.10	115,235.00	0.86
Sherman-Denison, TX	0.39	648,078.00	1,321.20	0.46	0.14	131,214.00	0.95
Shreveport-Bossier City, LA	0.50	1,110,198.00	1,247.36	0.65	0.26	439,631.00	0.79
Sierra Vista-Douglas, AZ	0.21	153,651.00	992.99	0.70	0.14	124,990.00	0.93
Sioux City, IA-NE-SD	0.24	383,415.00	1,137.14	0.41	0.14	168,218.00	0.97
Sioux Falls, SD	0.27	733,324.00	1,239.36	0.68	0.13	260,521.00	0.98
South Bend-Mishawaka, IN-MI	0.48	1,021,877.00	1,289.97	0.67	0.26	321,447.00	0.86
Spartanburg, SC	0.39	913,944.00	1,295.96	0.46	0.23	334,130.00	0.93
Spokane-Spokane Valley, WA	0.34	944,039.00	1,472.98	0.58	0.16	563,958.00	0.88
Springfield, IL	0.40	797,160.00	1,152.17	0.79	0.17	209,175.00	0.87
Springfield, MA	0.45	1,422,143.00	1,632.08	0.69	0.16	629,506.00	0.82

Continued on next page

Supplementary Table S21: (cont'd) Exposure Segregation and related variables (i.e. # exposures, mean SES, neighborhood sorting index, Gini index, population size, and bridging index by MSA

MSA	Exposure Segregation	# Exposures	Mean SES	Neighborhood Sorting Index	Gini	Pop. Size	Bridging Index
Springfield, MO	0.39	1,300,346.00	1,100.41	0.59	0.23	462,300.00	0.85
Springfield, OH	0.39	361,791.00	895.90	0.61	0.18	134,649.00	0.89
St. Cloud, MN	0.23	518,337.00	1,273.69	0.40	0.14	198,106.00	0.95
St. George, UT	0.27	243,300.00	1,715.91	0.35	0.17	165,859.00	0.94
St. Joseph, MO-KS	0.27	258,535.00	916.56	0.69	0.14	126,598.00	0.88
St. Louis, MO-IL	0.51	11,016,511.00	1,413.67	0.72	0.25	2,805,850.00	0.62
State College, PA	0.35	215,274.00	1,600.27	0.54	0.18	162,250.00	0.89
Staunton-Waynesboro, VA	0.24	282,626.00	1,324.76	0.28	0.17	121,984.00	0.94
Stockton-Lodi, CA	0.42	2,116,634.00	1,865.80	0.73	0.15	742,516.00	0.72
Sumter, SC	0.35	237,013.00	1,050.69	0.46	0.21	106,514.00	0.92
Syracuse, NY	0.37	1,932,194.00	1,483.28	0.63	0.19	651,048.00	0.82
Tallahassee, FL	0.45	1,973,991.00	1,428.85	0.71	0.22	383,467.00	0.75
Tampa-St. Petersburg-Clearwater, FL	0.45	24,564,393.00	1,805.89	0.62	0.22	3,091,225.00	0.73
Terre Haute, IN	0.32	369,258.00	930.48	0.48	0.18	170,022.00	0.94
Texarkana, TX-AR	0.30	240,478.00	956.50	0.61	0.12	150,254.00	0.89
The Villages, FL	0.39	786,309.00	1,711.00	0.69	0.12	124,933.00	0.90
Toledo, OH	0.53	1,775,752.00	1,217.56	0.69	0.25	603,830.00	0.63
Topeka, KS	0.43	1,205,672.00	1,084.47	0.64	0.20	233,153.00	0.93
Trenton, NJ	0.64	1,240,113.00	2,005.70	0.85	0.21	368,602.00	0.54
Tucson, AZ	0.39	2,564,383.00	1,362.93	0.73	0.17	1,027,502.00	0.72
Tulsa, OK	0.49	5,223,272.00	1,242.38	0.72	0.20	991,610.00	0.77
Tuscaloosa, AL	0.38	1,468,839.00	1,332.43	0.60	0.18	242,700.00	0.91
Twin Falls, ID	0.27	182,971.00	1,187.85	0.41	0.13	109,037.00	0.98
Tyler, TX	0.27	593,804.00	1,432.30	0.41	0.19	227,460.00	0.87
Urban Honolulu, HI	0.33	1,368,021.00	2,616.52	0.62	0.19	986,429.00	0.85
Utica-Rome, NY	0.31	374,846.00	1,123.98	0.66	0.17	292,336.00	0.80
Valdosta, GA	0.32	380,832.00	1,136.00	0.52	0.23	145,403.00	0.94
Vallejo-Fairfield, CA	0.18	1,878,258.00	2,372.07	0.67	0.11	443,877.00	0.85
Victoria, TX	0.37	374,597.00	1,529.97	0.55	0.20	99,651.00	0.94
Vineland-Bridgeton, NJ	0.37	354,594.00	1,371.71	0.63	0.09	151,748.00	0.90
VirGinia Beach-Norfolk-Newport News, VA-NC	0.45	6,944,774.00	1,666.43	0.62	0.20	1,724,876.00	0.75
Visalia-Porterville, CA	0.25	854,866.00	1,309.53	0.48	0.16	463,097.00	0.95
Waco, TX	0.41	1,245,450.00	1,334.52	0.55	0.20	268,550.00	0.89
Walla Walla, WA	0.22	53,561.00	1,448.66	0.39	0.13	64,675.00	1.00
Warner Robins, GA	0.40	600,913.00	1,271.38	0.54	0.20	191,227.00	0.86
Washington-Arlington-Alexandria, DC-VA-MD-WV	0.46	127,482,444.00	2,461.96	0.70	0.18	6,200,001.00	0.71
Waterloo-Cedar Falls, IA	0.38	326,152.00	1,111.17	0.62	0.18	169,553.00	0.84
Watertown-Fort Drum, NY	0.24	113,127.00	1,273.60	0.32	0.15	113,063.00	0.97
Wausau, WI	0.24	299,543.00	1,069.93	0.49	0.12	135,415.00	0.93
Weirton-Steubenville, WV-OH	0.24	235,799.00	823.09	0.52	0.12	118,181.00	0.94
Wenatchee, WA	0.21	126,600.00	1,748.88	0.31	0.14	118,646.00	1.00
Wheeling, WV-OH	0.26	102,043.00	1,100.65	0.53	0.15	141,228.00	0.87
Wichita Falls, TX	0.41	729,999.00	1,253.35	0.53	0.26	151,180.00	0.93
Wichita, KS	0.46	3,213,430.00	1,206.35	0.64	0.23	644,949.00	0.78
Williamsport, PA	0.23	148,520.00	1,025.19	0.38	0.14	113,930.00	0.99
Wilmington, NC	0.46	1,684,931.00	1,859.12	0.58	0.26	289,425.00	0.77
Winchester, VA-WV	0.33	296,124.00	1,560.81	0.50	0.16	138,107.00	0.95
Winston-Salem, NC	0.44	1,872,668.00	1,256.11	0.57	0.22	666,746.00	0.79
Worcester, MA-CT	0.48	2,650,313.00	1,777.27	0.71	0.17	942,303.00	0.74
Yakima, WA	0.33	222,165.00	1,236.55	0.54	0.15	250,377.00	0.88
York-Hanover, PA	0.41	1,303,401.00	1,430.52	0.56	0.18	445,722.00	0.85
Youngstown-Warren-Boardman, OH-PA	0.34	1,336,389.00	951.78	0.64	0.19	541,875.00	0.71
Yuba City, CA	0.29	309,905.00	1,574.77	0.57	0.13	173,213.00	0.89
Yuma, AZ	0.22	271,294.00	1,079.08	0.55	0.17	209,756.00	1.00

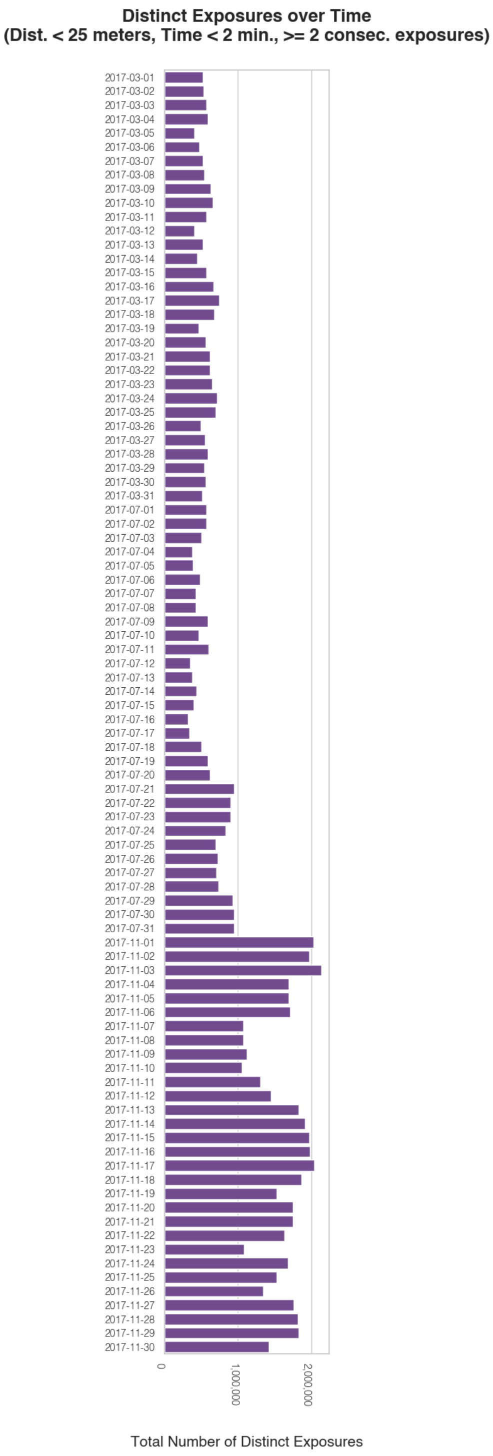

Supplementary Figure S60: Number of distinct exposures (distance threshold: 25 meters, time threshold: 2 minutes, length threshold: 2 consecutive exposures of five minute intervals) over time across all individuals residing in the 382 MSAs.

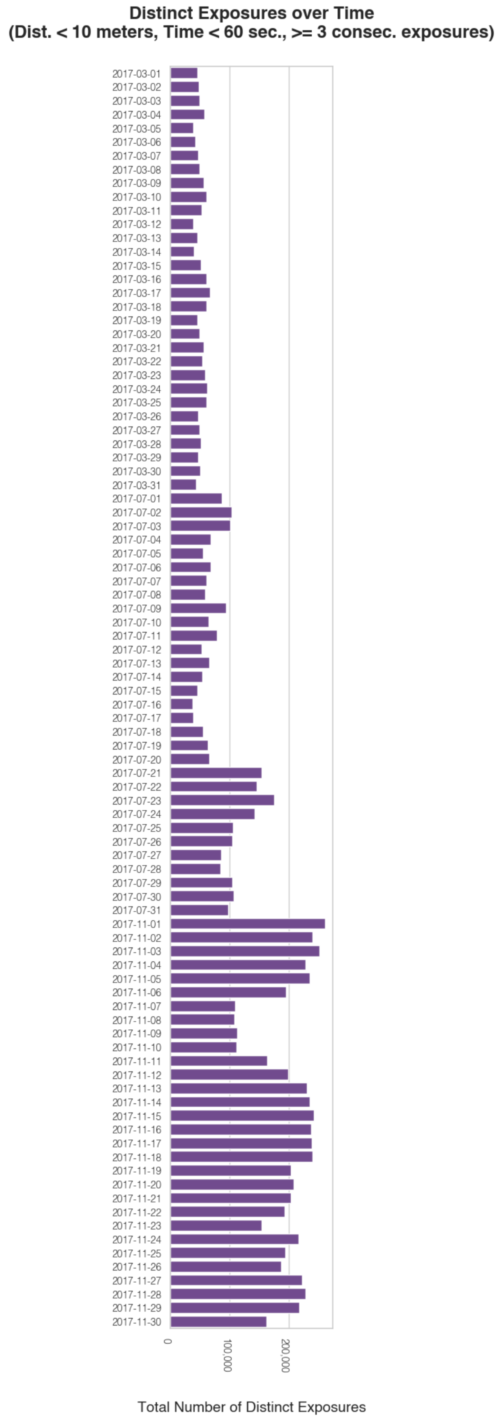

Supplementary Figure S61: Number of distinct exposures (distance threshold: 10 meters, time threshold: 60 seconds, length threshold: 3 consecutive exposures of five minute intervals) over time across all individuals residing in the 382 MSAs.

	Dependent variable:				Exposure Segregation	
	(1)	(2)	(3)	(4)	(5)	(6)
Intercept	0.355***	0.355***	0.355***	0.356***	0.355***	0.355***
	(0.004)	(0.004)	(0.004)	(0.003)	(0.003)	(0.003)
log(POI Density)	0.055***		0.035***	0.039***	0.020***	0.022***
	(0.004)		(0.004)	(0.005)	(0.004)	(0.004)
Gini Index (Estimated Rent)		0.064***	0.051***	0.052***	0.046***	0.047***
		(0.004)	(0.004)	(0.004)	(0.003)	(0.003)
Political Alignment (% Democrat in 2016 Election)				0.004		0.003
				(0.005)		(0.004)
Racial Demographics (% non-Hispanic White)				-0.003		0.002
				(0.004)		(0.003)
Mean SES (Estimated Rent)				-0.015***		-0.006
				(0.004)		(0.004)
Walkability (Walkscore)					0.003	0.003
					(0.003)	(0.004)
Commutability (% Commute to Work)					-0.010***	-0.010**
					(0.004)	(0.004)
Conventional Segregation (Neighborhood Sorting Index)					0.045***	0.043***
					(0.003)	(0.003)
Observations	382	382	382	376	382	376
R^2	0.307	0.419	0.526	0.540	0.690	0.688
Adjusted R^2	0.305	0.417	0.524	0.534	0.685	0.681

*p<0.1; **p<0.05; ***p<0.01

Supplementary Table S22: POI density is significantly associated with exposure segregation, after controlling for MSA income inequality (Gini index), political alignment (% Democrat in 2016 election), racial demographics (% non-Hispanic White), mean SES, walkability (Walkscore[9]), commutability (% of residents commuting to work), and residential segregation (neighborhood sorting index). This table is from an analogous regression to the regression shown in Extended Data Table 1, using density of POIs (average number of POIs within 10km of a resident) instead of population size (we look at each separately due to co-linearity between population size and POI density). Here we show the coefficients (after normalizing via z-scoring to have mean 0 and variance 1) from the primary specifications estimating the effect of POI density on exposure segregation across all MSAs. Standard errors are displayed in parentheses beneath each regression coefficient (*$p < 0.1$; **$p < 0.05$; ***$p < 0.01$; Two-sided Student's t-test; Methods 'Hypothesis testing'). Columns (1-5) are models specified with different subsets of covariates; Column 6 shows model specification with all covariates. Differences between sample size in models is due to missing data for several covariates in a small number of MSAs (Walkscores were not available for all MSAs)

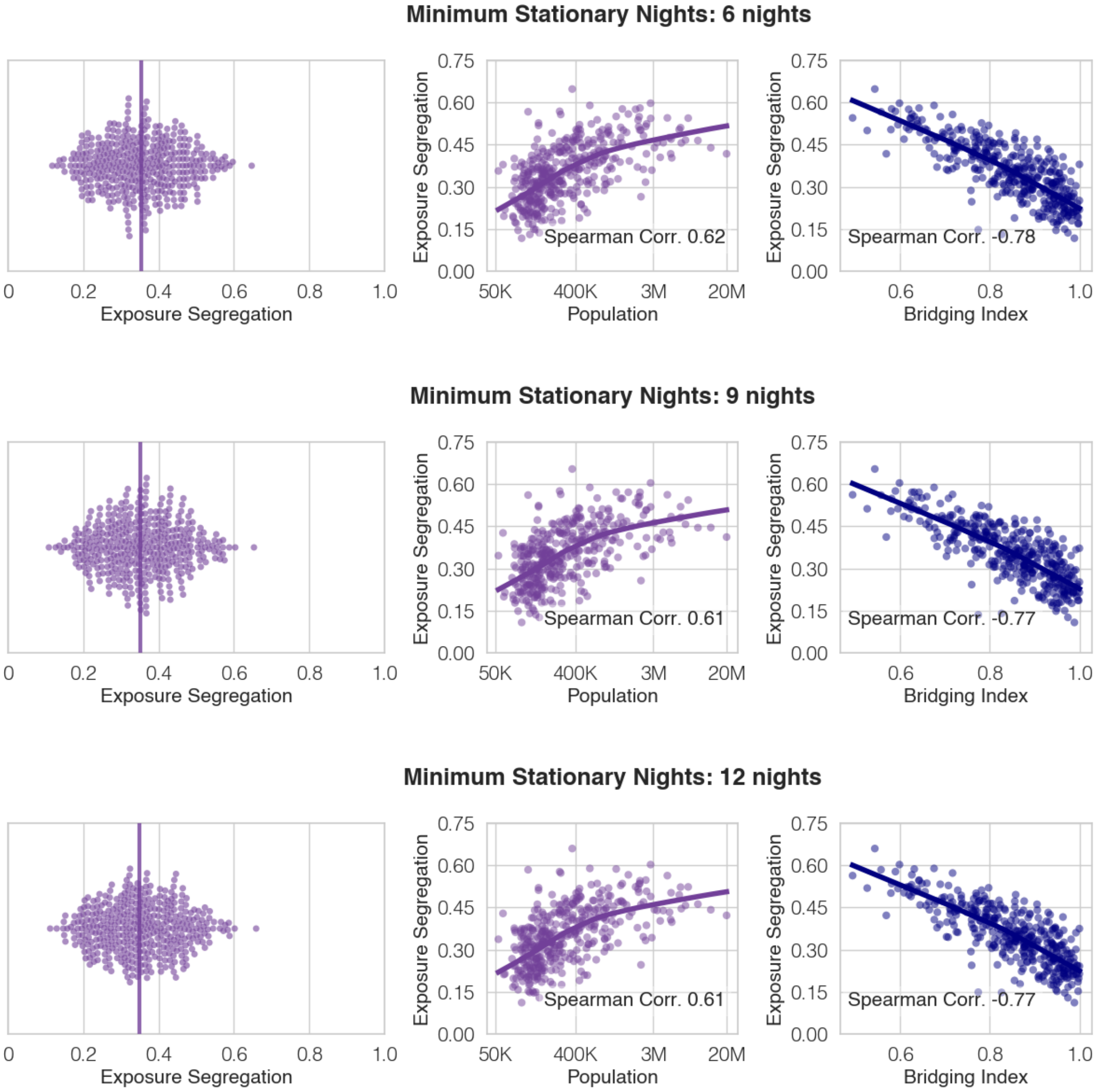

Supplementary Figure S62: Varying minimum stationary nights. We find that our primary study findings that, (1) large, dense cities are more segregated and (2) hub locations accessible to diverse individuals may mitigate segregation, are robust to varying thresholds of minimum stationary nights required, when identifying individual home locations. We increase the threshold from 3 (primary measure) to 6, 9, and 12 nights and find that are primary results remain unchanged.

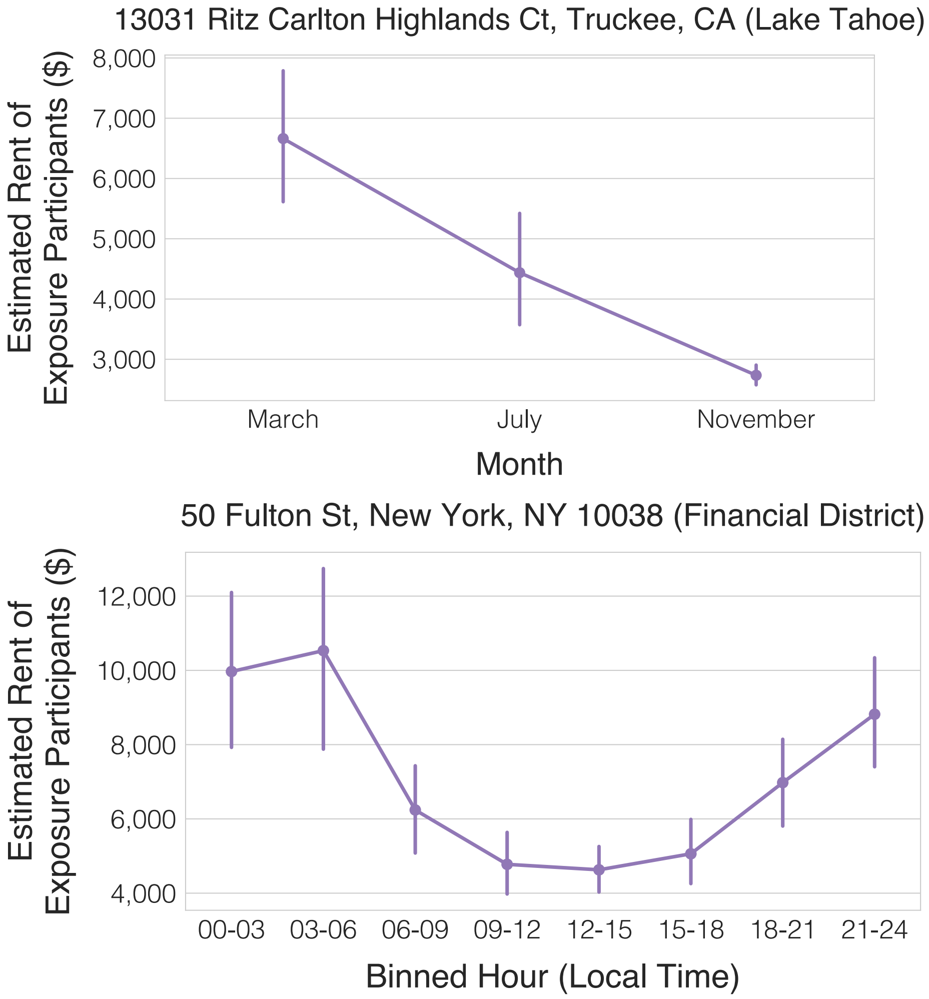

Supplementary Figure S63: Temporal heterogeneity in exposure participants at the same location: illustrative examples. SES of exposure participants is plotted as a function of calendar month (top) and hour of day (bottom). Y axis corresponds to mean SES values; error bars correspond to 95% bootstrap confidence intervals (Methods 'Hypothesis testing'). Top: a high-end hotel in the Lake Tahoe region of California. SES is highest during March due to high hotel demand (March is prime ski season in North Lake Tahoe). SES is lowest in November (weather is cold, but ski resorts are either closed or have little snow). N = 1093, 710, 651, 375 across March, July, and November respectively. Bottom: A multi-story building in the Manhattan Financial District. SES is lowest during daytime due to out-of-neighborhood visitors (there is a fast food restaurant, Papaya Dog, on the ground floor). SES is highest during nighttime hours due to exposures between residents (the cost of living in the Financial District is extremely high). N = 58, 35, 114, 167, 282, 175, 136, 98 across 00-03, 03-06, 06-09, 09-12, 12-15, 15-18, 18-21, 21-24 hours respectively.

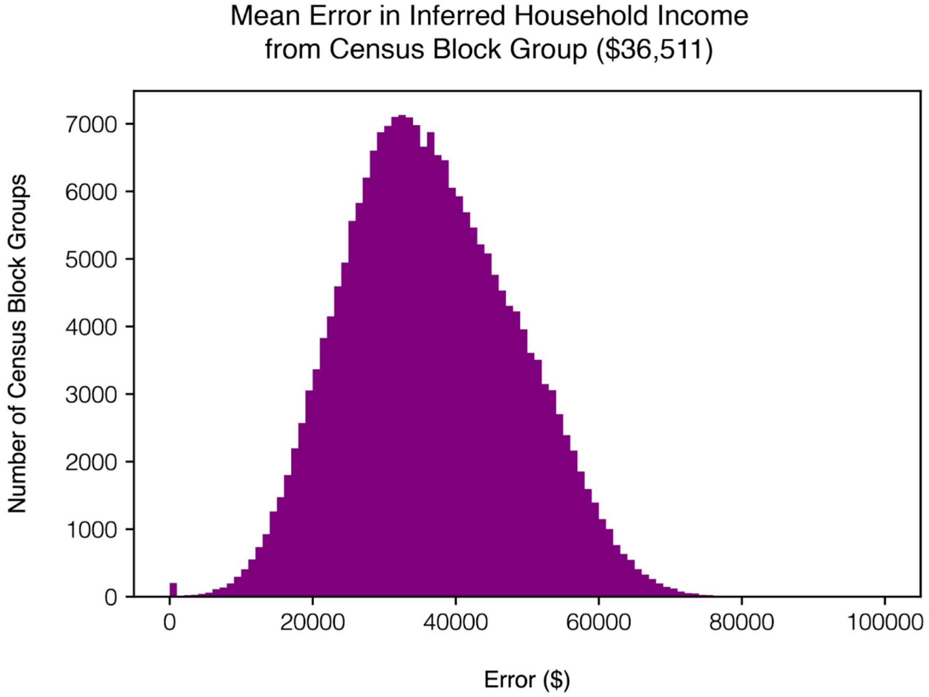

Supplementary Figure S64: Error from using census block group (CBG) income as a proxy for SES. A histogram of the estimated error from a inferring each individual's SES (operationalized as household income) from the mean value of their census block group (CBG). To compute the error, we use the counts of household income by block group provided by the US census. Consistent with prior work[15,16], we find that there is significant heterogeneity within each CBG, resulting in a mean error of $36,511.

	Dependent variable: Log(FB Social Connectedness)		
	(1)	(2)	(3)
const	11.123***	11.123***	11.123***
	(0.003)	(0.002)	(0.002)
Log(Distance km)	-1.163***		-0.253***
	(0.003)		(0.003)
Log(Exposure Network Social Connectness)		1.383***	1.190***
		(0.002)	(0.003)
Observations	118,559	118,559	118,559
R^2	0.539	0.763	0.773
Adjusted R^2	0.539	0.763	0.773

*p<0.1; **p<0.05; ***p<0.01

Supplementary Table S23: Our exposure network strongly predicts friendship formation (between counties). Here we show the coefficients (after normalizing via z-scoring to have mean 0 and variance 1) and R^2 from predicting FB network friendship strengths between counties. Standard errors are displayed in parentheses beneath each regression coefficient (*p < 0.1; **p < 0.05; ***p < 0.01; Two-sided Student's t-test; Methods 'Hypothesis testing'). Column (1) uses only county distance, Column (2) uses only exposure network social connectedness, and Column (3) uses the combination of distance and the exposure network. We find that our exposure network alone explains 76.3% of the variance in friendship formation between counties and is a stronger predictor of friendship formation than distance ($p < 10^{-4}$;Two-sided Steiger's's Z-test; Methods 'Hypothesis testing').

	Dependent variable: Log(FB Social Connectedness)		
	(1)	(2)	(3)
const	12.372***	12.372***	12.372***
	(0.001)	(0.001)	(0.001)
Log(Distance km)	-0.965***		-0.175***
	(0.001)		(0.002)
Log(Exposure Network Social Connectness)		1.183***	1.051***
		(0.001)	(0.002)
Observations	1,038,424	1,038,424	1,038,424
R^2	0.335	0.503	0.508
Adjusted R^2	0.335	0.503	0.508

*p<0.1; **p<0.05; ***p<0.01

Supplementary Table S24: Our exposure network strongly predicts friendship formation (between zip codes). Here we show the coefficients (after normalizing via z-scoring to have mean 0 and variance 1) and R^2 from predicting FB network friendship strengths between counties. Standard errors are displayed in parentheses beneath each regression coefficient (*p < 0.1; **p < 0.05; ***p < 0.01; Two-sided Student's t-test; Methods 'Hypothesis testing'). Column (1) uses only zip code distance, Column (2) uses only exposure network social connectedness, and Column (3) uses the combination of distance and the exposure network. We find that our exposure network alone explains 50.3% of the variance in friendship formation between zip codes and is a stronger predictor of friendship formation than distance ($p < 10^{-4}$; Two-sided Steiger's's Z-test; Methods 'Hypothesis testing').

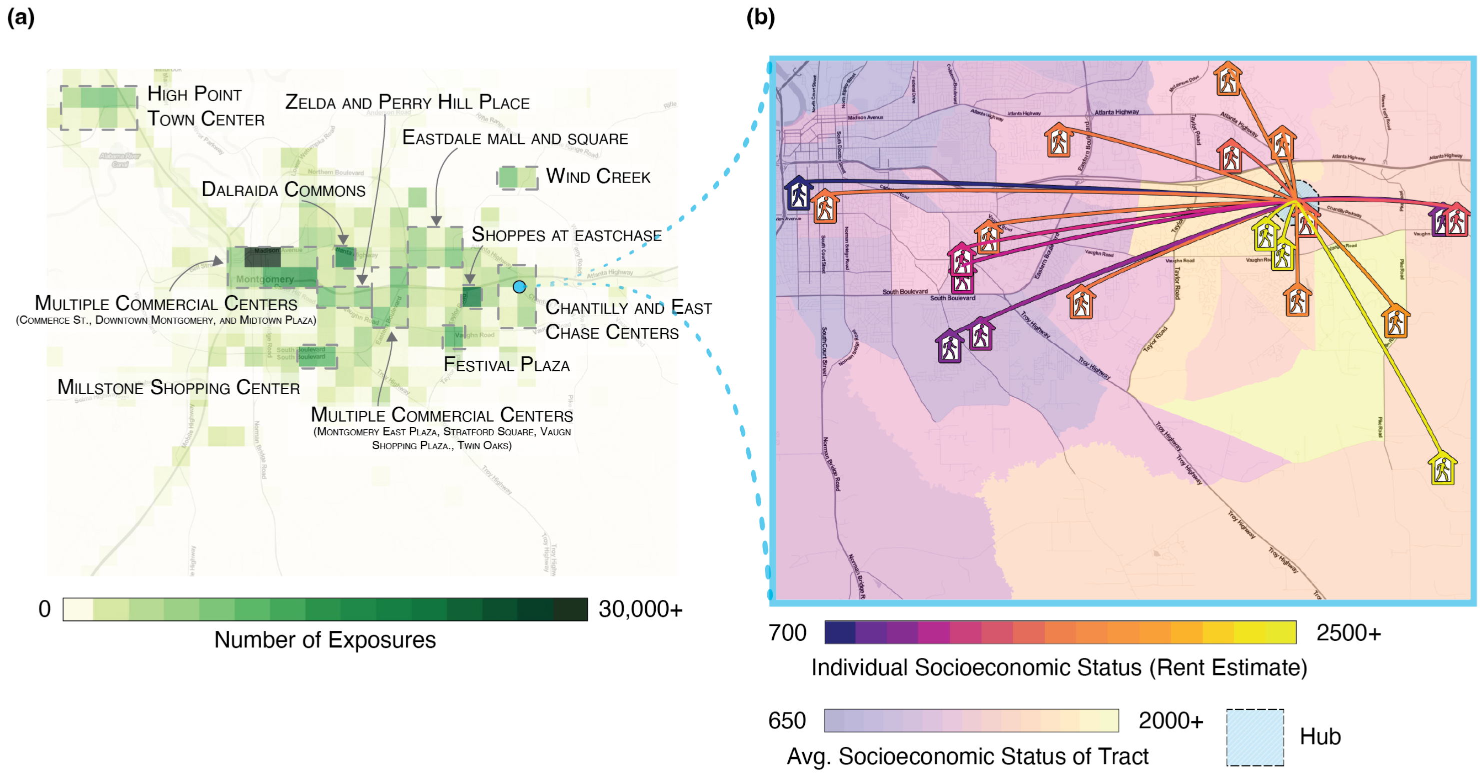

Supplementary Figure S65: Montgomery, AL. We conduct an analogous analysis to Figure 3c,d but for Montgomery, AL, which has nearly identical population (374K vs 385K residents) and income inequality (55th vs 60th percentile Gini index) to Fayetteville, NC but is 74% more segregated (88th percentile vs. 21st percentile exposure segregation). We find that the difference in segregation is explained by Montgomery, AL having a significantly higher bridging index compared to Fayetteville, NC (65th vs. 13th percentile). In Montgomery, AL hubs (i.e. commercial centers) are differentiated by SES which results in high-SES individuals and low-SES individuals visiting separate hubs and prevents them from engaging in cross-SES exposures. (a) shows that, as with all MSAs, commercial centers (e.g. shopping malls, plazas, etc.) are hubs of exposure. We illustrate that in Montgomery, AL all visually discernible hubs are associated with one or more commercial centers. (b) In Montgomery, AL, hubs are located in different locations which cater separate to high and low SES residents, leading to segregated exposures. As an illustrative example, we show a zoomed-in map of one hub (Chantilly Center) in Montgomery, AL, and display a random sample of 10 exposures occurring inside of it. Chantilly Center in Montgomery, AL is located accessibly for high SES individuals but is far apart from low-SES tracts. As a result, the sample shows that the majority of exposures are middle-upper SES, and only a few low-SES individuals visit Chantilly Center and cross paths with these high-SES individuals. Home icons demarcate individual home location (up to 100 meters of random noise added to preserve anonymity); home colors denote individual SES; arcs indicate an exposure inside of the hub; background colors indicate mean census tract SES.

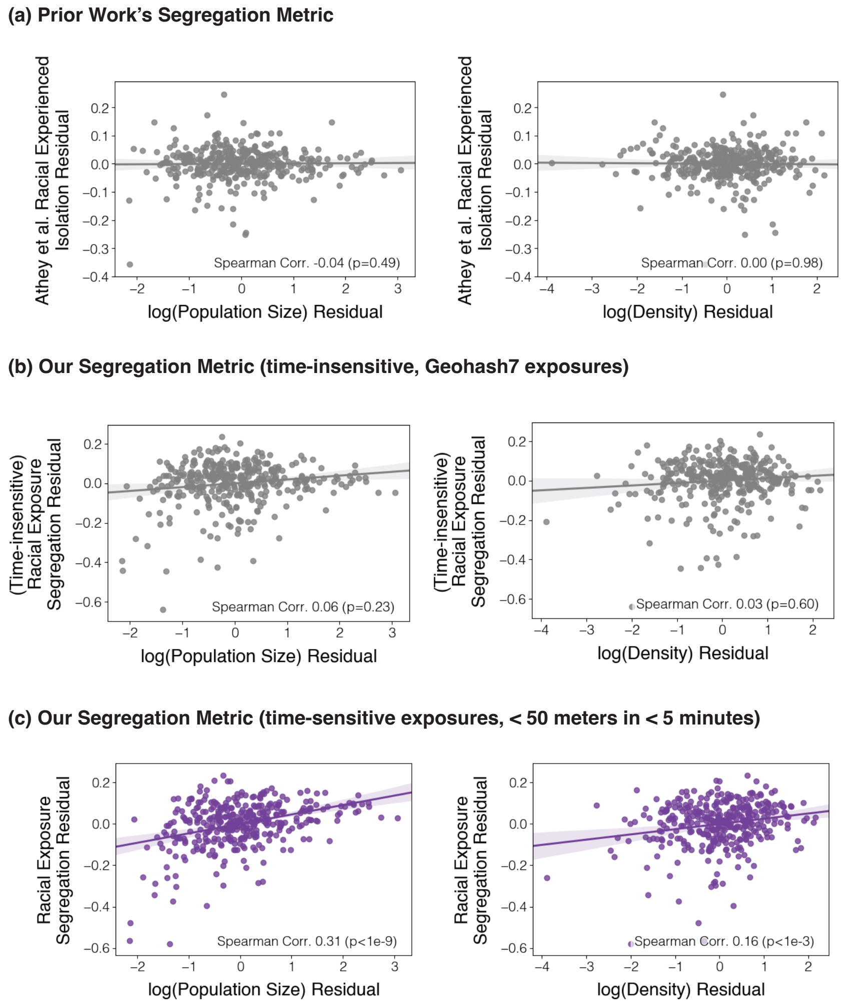

Supplementary Figure S66: Our time-sensitive and high-resolution measure of exposure enables us to reject the cosmopolitan mixing hypothesis. We analyze the consequences of different measures of exposure by reproducing Athey et al.'s assessment of the cosmopolitan mixing hypothesis[1]. Athey et al. estimate the correlation between population density and their racial segregation metric ("experienced isolation"), after controlling for residential (racial) segregation. They do so by correlating the residuals of their racial segregation metric with the residuals of population density, where the residuals are derived from regression on (racial) residential segregation controls. We extend this analysis to include two additional segregation metrics, revealing that a time-sensitive measure of exposure is necessary to reject the cosmopolitan mixing hypothesis. Lines show show best linear fit along with shaded 95% bootstrap confidence intervals. **(a)** We first consider Athey et al.'s original racial segregation metric ("experienced isolation", which considers two people exposed to each other if they ever visited the same 153 x 153m Geohash7 grid cell within 4 months). Under Athey et al.'s metric, there is no significant correlation between population size and racial segregation (Spearman Corr. -0.04, $p = 0.49$; Two-sided Student's t-test; Methods 'Hypothesis testing'), nor between population density and experienced segregation (Spearman Corr. 0.00, $p = 0.98$; Two-sided Student's t-test; Methods 'Hypothesis testing'). Athey et al.'s robustness checks further corroborate this point, showing no significant association between population density and segregation when MSAs are unweighted by population size (Athey et al. Supplementary Table S8). **(b)** We then consider a time-insensitive variant of our own racial exposure segregation metric (Supplementary Figure S34), which uses Athey et. al's definition of exposure (any two people who visited the same 153 x 153m Geohash7 grid cell are considered exposed). Here, we similarly find that there is no significant correlation between population size and racial segregation (Spearman Corr. 0.06, $p = 0.23$; Two-sided Student's t-test; Methods 'Hypothesis testing'), nor between population density and experienced segregation (Spearman Corr. 0.03, $p = 0.60$; Two-sided Student's t-test; Methods 'Hypothesis testing') **(c)** Finally, when we use our own time-sensitive and high-resolution racial exposure segregation metric, we find positive and statistically significant relationships between racial exposure segregation and population size (Spearman Corr. 0.31, $p < 1 \times 10^{-9}$; Two-sided Student's t-test; Methods 'Hypothesis testing') as well as population density (Spearman Corr. 0.16, $p < 0.001$; Two-sided Student's t-test; Methods 'Hypothesis testing'). This comparison shows that precisely measuring exposure is necessary to reject the cosmopolitan mixing hypothesis.

Supplementary Information References

1. Athey, S., Ferguson, B., Gentzkow, M. & Schmidt, T. Estimating experienced racial segregation in us cities using large-scale gps data. *Proceedings of the National Academy of Sciences* **118**, e2026160118 (2021).

2. Brown, J. R., Enos, R. D., Feigenbaum, J. & Mazumder, S. Childhood cross-ethnic exposure predicts political behavior seven decades later: Evidence from linked administrative data. *Science Advances* **7**, eabe8432 (2021).

3. Merry, K. & Bettinger, P. Smartphone gps accuracy study in an urban environment. *PloS one* **14**, e0219890 (2019).

4. Menard, T., Miller, J., Nowak, M. & Norris, D. Comparing the gps capabilities of the samsung galaxy s, motorola droid x, and the apple iphone for vehicle tracking using freesim_mobile. In *2011 14th International IEEE Conference on Intelligent Transportation Systems (ITSC)*, 985–990 (IEEE, 2011).

5. Crandall, D. J. *et al.* Inferring social ties from geographic coincidences. *Proceedings of the National Academy of Sciences* **107**, 22436–22441 (2010).

6. Barabási, A.-L. Network science. *Philosophical Transactions of the Royal Society A: Mathematical, Physical and Engineering Sciences* **371**, 20120375 (2013).

7. Maslov, S. & Sneppen, K. Specificity and stability in topology of protein networks. *Science* **296**, 910–913 (2002).

8. Dunbar, R. *How many friends does one person need?: Dunbar's number and other evolutionary quirks* (Faber & Faber, 2010).

9. Walkscore. Walkscore walkability estimates (2020). Accessed August 30, 2020.

10. Granovetter, M. S. The strength of weak ties. *American journal of sociology* **78**, 1360–1380 (1973).

11. Rajkumar, K., Saint-Jacques, G., Bojinov, I., Brynjolfsson, E. & Aral, S. A causal test of the strength of weak ties. *Science* **377**, 1304–1310 (2022).

12. Ripley, A. The geography of partisan prejudice (2021).

13. Cromartie, J. Rural-urban commuting area codes (2005).

14. Bailey, M., Cao, R., Kuchler, T., Stroebel, J. & Wong, A. Social connectedness: Measurement, determinants, and effects. *Journal of Economic Perspectives* **32**, 259–80 (2018).

15. Soobader, M.-j., LeClere, F. B., Hadden, W. & Maury, B. Using aggregate geographic data to proxy individual socioeconomic status: does size matter? *American Journal of Public Health* **91**, 632 (2001).

16. Geronimus, A. T. & Bound, J. Use of census-based aggregate variables to proxy for socioeconomic group: evidence from national samples. *American journal of epidemiology* **148**, 475–486 (1998).
