# Supplementary material for: Human mobility networks reveal increased segregation in large cities
Source: Nature. 2023 Nov 29;624(7992):586–92. doi: 10.1038/s41586-023-06757-3 (PMC10733138; doi:10.1038/s41586-023-06757-3)
Supplement: Supplementary file 2 — Reporting Summary [file 41586_2023_6757_MOESM2_ESM.pdf]

nature portfolio

Corresponding author(s): Jure Leskovec

Last updated by author(s): Aug 22, 2023

Reporting Summary

Nature Portfolio wishes to improve the reproducibility of the work that we publish. This form provides structure for consistency and transparency in reporting. For further information on Nature Portfolio policies, see our Editorial Policies and the Editorial Policy Checklist.

Statistics

For all statistical analyses, confirm that the following items are present in the figure legend, table legend, main text, or Methods section.

n/a	Confirmed	
☐	☒	The exact sample size (*n*) for each experimental group/condition, given as a discrete number and unit of measurement
☐	☒	A statement on whether measurements were taken from distinct samples or whether the same sample was measured repeatedly
☐	☒	The statistical test(s) used AND whether they are one- or two-sided<br>*Only common tests should be described solely by name; describe more complex techniques in the Methods section.*
☐	☒	A description of all covariates tested
☐	☒	A description of any assumptions or corrections, such as tests of normality and adjustment for multiple comparisons
☐	☒	A full description of the statistical parameters including central tendency (e.g. means) or other basic estimates (e.g. regression coefficient) AND variation (e.g. standard deviation) or associated estimates of uncertainty (e.g. confidence intervals)
☐	☒	For null hypothesis testing, the test statistic (e.g. *F*, *t*, *r*) with confidence intervals, effect sizes, degrees of freedom and *P* value noted<br>*Give P values as exact values whenever suitable.*
☒	☐	For Bayesian analysis, information on the choice of priors and Markov chain Monte Carlo settings
☒	☐	For hierarchical and complex designs, identification of the appropriate level for tests and full reporting of outcomes
☐	☒	Estimates of effect sizes (e.g. Cohen's *d*, Pearson's *r*), indicating how they were calculated

Our web collection on statistics for biologists contains articles on many of the points above.

Software and code

Policy information about availability of computer code

Data collection	No data collection was performed for this study; all analysis relied on previously collected datasets, as described in the Data section below.
Data analysis	Primary data analysis was performed using Python version 3.9.5 and standard libraries. Ping interpolation was performed with SciPy version 1.4.0. Exposure segregation was estimated using R version 3.6.3 and the lme4 library version 1.1-21. Code is available at https://github.com/snap-stanford/exposure-segregation

For manuscripts utilizing custom algorithms or software that are central to the research but not yet described in published literature, software must be made available to editors and reviewers. We strongly encourage code deposition in a community repository (e.g. GitHub). See the Nature Portfolio guidelines for submitting code & software for further information.

Data

Policy information about availability of data

All manuscripts must include a data availability statement. This statement should provide the following information, where applicable:

- Accession codes, unique identifiers, or web links for publicly available datasets
- A description of any restrictions on data availability
- For clinical datasets or third party data, please ensure that the statement adheres to our policy

Nationwide exposure segregation and bridging index measures are available at http://segregation.stanford.edu. Census (https://www.census.gov/programs-surveys/acs), Zillow (https://www.zillow.com/howto/api/APIOverview.htm), and TIGER (https://www.census.gov/geographies/mapping-files/time-series/geo/tiger-

geodatabase-file.html) data are publicly available. CoreLogic database is commercially available and may be requested for research use (https://www.corelogic.com/contact/). Individual cell phone mobility data are not publicly available to preserve privacy, while mobility data aggregated to the Census block group (CBG level) and SafeGraph places data are commercially available and may be requested for research use (https://www.safegraph.com/contact-us).

Human research participants

Policy information about studies involving human research participants and Sex and Gender in Research.

Reporting on sex and gender	Sex and gender not collected.
Population characteristics	Our primary analysis sample was constructed from previously collected, de-identified mobility data provided by the company SafeGraph (https://www.safegraph.com/). We filtered individuals to those with at least a total of 500 pings across three evenly spaced the months in 2017: March, July, and November. We further excluded individuals that shared 80% or more identical pings with another individual, did not have any pings with < 100 meters accuracy, or for whom the Zillow API did not return an estimated rent value. Our final analysis sample consisted of 9,567,559 cell phones.
Recruitment	See above. As described by in public documentation, SafeGraph data is collected by: "partner[ing] with mobile applications that obtain opt-in consent from its users to collect anonymous location data." SafeGraph ensures that its mobile application partners obtain consent for data to be used for commercial and research purposes, including academic publication. SafeGraph users are able to opt-out of data collection at any time. Prior work has investigated biases in the SafeGraph dataset (https://www.safegraph.com/blog/what-about-bias-in-the-safegraph-dataset)
Ethics oversight	Stanford University IRB

Note that full information on the approval of the study protocol must also be provided in the manuscript.

Field-specific reporting

Please select the one below that is the best fit for your research. If you are not sure, read the appropriate sections before making your selection.

☐ Life sciences ☒ Behavioural & social sciences ☐ Ecological, evolutionary & environmental sciences

For a reference copy of the document with all sections, see nature.com/documents/nr-reporting-summary-flat.pdf

Behavioural & social sciences study design

All studies must disclose on these points even when the disclosure is negative.

Study description	This is a quantitative, retrospective observational study.
Research sample	We study previously collected, de-identified mobility data from provided by the company SafeGraph. As described by SafeGraph in public documentation, SafeGraph data is collected by: "partner[ing] with mobile applications that obtain opt-in consent from its users to collect anonymous location data. This data is not associated with any name or email address. This data includes the latitude and longitude of a device at a given point in time." SafeGraph ensures that its mobile application partners obtain consent for data to be used for commercial and research purposes, including academic publication. SafeGraph users are able to opt-out of data collection at any time. While SafeGraph data is not a random sample, it is geographically well-balanced (i.e., an approximately unbiased sample of different census tracts within each State), and well-balanced along the dimensions of race, income, and education. SafeGraph data was chosen as the study sample because of its scale, geographical breadth, and as it is a widely used standard in previous studies of human mobility. This data was joined with Census (demographics), Zillow (estimated rent), SafeGraph Places (POIs), CoreLogic (addresses), and TIGER (roads and railways) data.
Sampling strategy	We did not perform sampling, sample size (N=9,567,559) was determined by the size of the SafeGraph database after filtering (see below). SafeGraph anonymized cell phone data has been shown to be geographically representative across on many key racial, economic, and demographic variables (https://www.safegraph.com/blog/what-about-bias-in-the-safegraph-dataset).
Data collection	We did not perform data collection, but relied on previously collected, anonymized geolocation data by the company SafeGraph.
Timing	Three (evenly space) months from 2017: March, July, and November.
Data exclusions	We apply several filters to improve reliability of the SafeGraph data: all participants logged at least 500 pings, had ping locations with an accuracy < 100 meters, and were at least 20% distinct from other participants (de-duplication).
Non-participation	SafeGraph users are able to opt-out of data collection at any time (by changing application settings). Data provided by SafeGraph does not distinguish between user inactivity and opting out from data collection.
Randomization	Observational study, participants were not randomized. Key findings are robust to controlling (via regression covariates) for MSA-

Randomization	level income inequality, political demographics, racial demographics, mean economic standing, walkability, commutability and residential segregation.

Reporting for specific materials, systems and methods

We require information from authors about some types of materials, experimental systems and methods used in many studies. Here, indicate whether each material, system or method listed is relevant to your study. If you are not sure if a list item applies to your research, read the appropriate section before selecting a response.

Materials & experimental systems

n/a	Involved in the study	
☒	☐	Antibodies
☒	☐	Eukaryotic cell lines
☒	☐	Palaeontology and archaeology
☒	☐	Animals and other organisms
☒	☐	Clinical data
☒	☐	Dual use research of concern

Methods

n/a	Involved in the study	
☒	☐	ChIP-seq
☒	☐	Flow cytometry
☒	☐	MRI-based neuroimaging
